# Supplementary material for: The effects of genital myiasis on the diversity of the vaginal microbiota in female Bactrian camels
Source: BMC Vet Res. 2022 Mar 5;18:87. doi: 10.1186/s12917-022-03189-5 (PMC8897907; doi:10.1186/s12917-022-03189-5)
Supplement: Supplementary file 5 — Additional file 5. [file 12917_2022_3189_MOESM5_ESM.zip › MPL201709200_16s_yy/Treat1/report.html]

菌群多样性组成谱研究报告


## 菌群多样性组成谱研究报告

隐藏目录全部展开

- + 项目概况及整体流程介绍

- 项目概况
- 分析项目
- 实验流程
- 分析流程

- + 原始测序数据的筛查、处理和质量控制

- 原始双端测序数据的处理
- 疑问序列的剔除及序列数统计
- 序列长度分布统计

- + OTU划分和分类地位鉴定

- OTU划分
- OTU分类地位鉴定
- OTU精简和分类鉴定结果统计
- 共有OTU分析

- + Alpha多样性分析

- Rarefaction稀疏曲线
- Specaccum物种累积曲线- 丰度等级曲线
  - Alpha多样性指数计算

- + 分类学组成分析

- 各分类水平的微生物类群数统计
- 各分类水平的分类学组成分析
- 样本（组）间分类学组成的差异分析
- 系统发育树和分类等级树的构建
- 热图分析

- + Beta多样性分析

- PCA主成分分析
- 基于UniFrac距离的PCoA主坐标分析
- 基于UniFrac距离的NMDS非度量多维尺度分析
- 基于UniFrac距离的样本聚类分析
- UniFrac距离组间/组内差异比较分析

- + 菌群比较分析和关键物种筛选

- PLS-DA偏最小二乘法判别分析
- Adonis/PERMANOVA多元方差分析和置换检验
- ANOSIM相似度分析
- 随机森林分析
- RDA冗余分析

- + 关联网络分析

- 优势物种互作Spearman关联网络分析

- + 菌群代谢功能预测
- 参考文献
- 附录：文档结构

项目名称：菌群多样性组成谱研究

委托单位：北京微生太科技有限公司

制定日期：
2017年09月19日

---

## 项目概况及整体流程介绍

### I 项目概况

|  |  |
| --- | --- |
| ﻿项目编号 | YF106-M201709014 |
| 开题单号 | MPL201709200 |
| 项目类型 | 菌群多样性组成谱研究 |
| 样本数目 | 23 |
| 测序平台 | Illumina Miseq |
| 分析项目 | 标准生物信息分析 |
| 分析者工号 | P20150099 |
| 完成日期 | 2017-09-19 |

---

### II 分析项目

|  |  |  |  |
| --- | --- | --- | --- |
| ﻿分析项目 | 类别 | 分析要求 | 目录 |
| **原始测序数据的质控** |  |  |  |
| - 原始测序数据的处理 | A |  | A01\_rawdata |
| - 疑问序列的剔除 | A |  | A01\_rawdata |
| - 高质量序列长度分布统计 | A |  | A02\_sequences |
| **序列归并和OTU划分** |  |  |  |
| - OTU划分 | B |  | B01\_OTU |
| - OTU分类地位鉴定 | B |  | B01\_OTU |
| - OTU精简和分类鉴定结果统计 | B |  | B01\_OTU |
| - 多样本共有OTU的Venn图分析 | B | 样本（组）≥ 2且≤ 5 | B01\_OTU/venn |
| **Alpha多样性分析** |  |  |  |
| - Rarefaction稀疏曲线 | B |  | B02\_arare |
| - Specaccum物种累积曲线 | B | 样本≥ 10 | B03\_specaccum |
| - 丰度等级曲线 | B |  | B04\_rabund |
| - Alpha多样性指数计算 | B |  | B05\_index |
| **分类组成分析** |  |  |  |
| - 各分类水平的微生物类群数统计 | B |  | B06\_taxa |
| - 各分类水平的分类学组成分析 | B |  | B07\_taxa\_summary |
| - Metastats分析 | C | 样本（组）≥ 2 | C01\_diff/metastats |
| - LEfSe分析 | C | 分组≥ 2 | C01\_diff/lefse |
| **菌群组成交互式可视化** |  |  |  |
| - 系统发育树构建 | B |  | B08\_megan |
| - MEGAN可视化展示 | B |  | B08\_megan |
| - GraPhlAn可视化展示 | B |  | B09\_graphlan |
| - Krona交互式展示 | B |  | B10\_krona |
| - 热图分析 | B | 样本≥ 3 | B11\_heatmap |
| **Beta多样性分析** |  |  |  |
| - PCA分析 | B | 样本≥ 3 | B12\_pca |
| - UniFrac-PCoA分析 | B | 样本≥ 3 | B13\_bdiv |
| - UniFrac-MDS分析 | B | 样本≥ 5 | B13\_bdiv |
| - UniFrac-UPGMA聚类分析 | B | 样本≥ 3 | B13\_bdiv |
| - UniFrac距离组间/组内差异比较分析 | B | 分组≥ 2 | B13\_bdiv |
| **菌群比较分析和关键物种筛选** |  |  |  |
| - PLS-DA分析 | C | 分组≥ 2 | C02\_plsda |
| - Adonis/PERMANOVA分析 | C | 样本≥ 3，并提供影响因素数值；或分组≥ 2 | C03\_adonis |
| - ANOSIM分析 | C | 分组≥ 2 | C04\_anosim |
| - 随机森林分析 | C | 分组≥ 2 | C05\_random\_forests |
| - RDA分析 | B | 样本≥ 3，并提供影响因素数值；或分组≥ 2 | 22\_rda |
| **关联网络分析** |  |  |  |
| - 优势物种互作Spearman关联网络分析 | C | 样本≥ 3 | C06\_network |
| **菌群代谢功能预测** |  |  |  |
| - PICRUSt功能预测分析 | C | 仅限16S rRNA基因数据 | C07\_picrust |
| - 功能类群分布柱状图分析 | C | 无分组 | C07\_picrust |
| - 功能类群分布小提琴图分析 | C | 分组≥ 2 | C07\_picrust |
| - 共有功能类群的Venn图分析 | C | 样本（组）≥ 2且≤ 5 | C07\_picrust |
| - 结合聚类分析的功能类群热图分析 | C | 样本≥ 3 | C07\_picrust |

A：基本信息分析；B：常规信息分析；C：高级信息分析；D：深度信息分析

---

### III 实验流程

从DNA提取到上机测序，需要严谨、可靠的样本检测、质控流程，在每一环节都对样本质量严格控制，才能确保测序数据的真实可信。

### 1. 微生物组总DNA提取

对于各种不同来源的微生物组样本，根据过往项目经验，选用最合适的微生物组总DNA提取方法，并通过0.8%琼脂糖凝胶电泳检测DNA提取质量，同时采用紫外分光光度计对DNA进行定量。

### 2. 目标片段PCR扩增

通常以微生物核糖体RNA等能够反映菌群组成和多样性的目标序列为靶点，根据序列中的保守区域设计相应引物，并添加样本特异性Barcode序列，进而对rRNA基因可变区（单个或连续的多个）或特定基因片段进行PCR扩增。

PCR扩增采用NEB公司的Q5高保真DNA聚合酶，并严格控制扩增循环数，使循环数尽可能低的同时，也保证同一批样本的扩增条件一致。

### 3. 扩增产物回收纯化

PCR扩增产物通过2%琼脂糖凝胶电泳进行检测，并对目标片段进行切胶回收，回收采用AXYGEN公司的凝胶回收试剂盒。

### 4. 扩增产物荧光定量

参照电泳初步定量结果，将PCR扩增回收产物进行荧光定量，荧光试剂为Quant-iT PicoGreen dsDNA Assay Kit，定量仪器为Microplate reader（BioTek，FLx800）。根据荧光定量结果，按照每个样本的测序量需求，对各样本按相应比例进行混合。

### 5. 测序文库制备

以Illumina MiSeq测序为例，采用Illumina公司的TruSeq Nano DNA LT Library Prep Kit制备测序文库。

1) 首先对上述扩增产物进行序列末端修复，通过试剂盒中的End Repair Mix2切除DNA序列5’端的突出碱基，同时添加一个磷酸基团、补齐3’端的缺失碱基；

2) 在DNA序列的3’端添加A碱基以防止DNA片段自连，同时保证目标序列能与测序接头相连（测序接头3’端有一个突出的T碱基）；

3) 在序列5’端添加含有文库特异性标签（即Index序列）的测序接头，使DNA分子能被固定在Flow Cell上；

4) 采用BECKMAN AMPure XP Beads，通过磁珠筛选去除接头自连片段，纯化添加接头后的文库体系；

5) 对上述连上接头的DNA片段进行PCR扩增，从而富集测序文库模板，并采用BECKMAN AMPure XP Beads再次纯化文库富集产物；

6) 通过2%琼脂糖凝胶电泳，对文库做最终的片段选择与纯化。

### 6. 上机进行高通量测序

1) 上机测序前，需要先对文库在Agilent Bioanalyzer上进行质检，采用Agilent High Sensitivity DNA Kit。合格的文库有且只有单一的峰，且无接头。

2) 之后，采用Quant-iT PicoGreen dsDNA Assay Kit在Promega QuantiFluor荧光定量系统上对文库进行定量，合格的文库浓度应在2nM以上。

3) 将合格的各上机测序文库（Index序列不可重复）梯度稀释后，根据所需测序量按相应比例混合，并经NaOH变性为单链进行上机测序；

4) 使用MiSeq测序仪进行2×300bp的双端测序，相应试剂为MiSeq Reagent Kit V3 (600 cycles)。

由于MiSeq测序读长较短的特性，同时也为了保证测序质量，建议目标片段的最佳测序长度为200~450 bp。

核糖体RNA含有多个保守区和高度可变区，通常我们利用保守区域设计引物来扩增 rRNA基因的单个或多个可变区，然后测序分析微生物多样性。由于MiSeq测序读长的限制，同时也为了保证测序质量，最佳测序的插入片段范围是200-450bp。

---

### IV 分析流程

1. 首先对高通量测序的原始下机数据根据序列质量进行初步筛查；

2. 通过质量初筛的序列按照引物和Barcode信息，识别分配入对应样本，并去除嵌合体等疑问序列；

3. 对获得的序列进行OTU归并划分，每个OTU的代表序列用于分类地位鉴定以及系统发育学分析；

4. 根据OTU在不同样本中的丰度分布，评估每个样本的多样性水平，并通过稀疏曲线反映测序深度是否达标；

5. 对各样本（组）在不同分类水平的具体组成进行分析（并检验组间是否具有统计学差异）；

6. 通过多种多变量统计学分析工具，进一步衡量不同样本（组）间的菌群结构差异及与差异相关的物种；

7. 根据物种在各样本中的组成分布，构建互作关联网络；

8. 根据16S rRNA基因测序结果，还可预测各样本的菌群代谢功能。

在以上结果的基础上，使用多种数据可视化和交互式工具，绘制具备论文发表水准的二维/三维图表，全方位、客观地呈现以上分析结果。

---

## 原始数据整理、过滤及质量评估

### 原始双端测序数据的处理

本项目采用Illumina MiSeq平台对群落DNA片段进行双端（Paired-end）测序。测序原始数据以FASTQ格式保存（R1.fastq和R2.fastq，Read 1和Read 2序列一一配对）。FASTQ是一种存储序列信息的特定文本格式，推荐用Notepad++等文本编辑器打开，每四行对应一条测序Read：第一行以符号“@”起始，对应于序列ID和相应的描述信息；第二行为实际测得的碱基序列；第三行以符号“+”起始；而第四行的字符串则记录了第二行序列中，每个碱基所对应的测序质量（详见https://en.wikipedia.org/wiki/Fastq）。

@M00200:111:000000000-A6VNV:1:1101:15594:1337 1:N:0:6

ACGCGGGTATCTAATCCTGTTTGCTCCCCACGCTTTCGCGCCTCAGTGTCAGTTAC

+

ABABADBBDFFFGGGFGGGFGGHGBGHGGHGGGGGGHGGGGGGGHHGGFBGEGGEG

**FASTQ格式示例**

#### 分析步骤

为了整合原始双端测序数据，首先采用滑动窗口法对FASTQ格式的双端序列逐一作质量筛查：窗口大小为10 bp，步长为1 bp，从5’端第一个碱基位置开始移动，要求窗口中碱基平均质量≥ Q20（即碱基平均测序准确率≥ 99%），从第一个平均质量值低于Q20的窗口处截断序列，并要求截断后的序列长度≥ 150 bp，且不允许存在模糊碱基（Ambiguous base）N。随后，利用FLASH软件（v1.2.7，http://ccb.jhu.edu/software/FLASH/）(Magoc and Salzberg, 2011)，对通过质量初筛的双端序列根据重叠碱基进行配对连接：要求Read 1和Read 2两条序列的重叠碱基长度≥ 10 bp，且不允许碱基错配。最后，根据每个样本所对应的Index信息（即Barcode序列，为序列起始处用于识别样本的一小段碱基序列），将连接后的序列识别分配入对应样本（要求Index序列完全匹配），从而获得每个样本的有效序列。


#### 结果目录: A01\_rawdata

---

### 疑问序列的剔除及序列数统计

高通量测序过程中，可能产生一系列错误或有疑问的序列。比如，PCR扩增可能产生嵌合体序列（Chimera sequence），而测序本身也可能产生碱基替换、插入删除等错误。为了保证分析结果的可靠准确，必须对上述提取获得的有效序列的质量作进一步的评估，从而获得可用于后续分析的序列。

#### 分析步骤

首先运用QIIME软件（Quantitative Insights Into Microbial Ecology，v1.8.0，http://qiime.org/）(Caporaso et al., 2010)识别疑问序列。除了要求序列长度≥ 150 bp，且不允许存在模糊碱基N之外，我们还将剔除：1) 5’端引物错配碱基数> 1的序列；2) 含有连续相同碱基数>8的序列。

随后，通过QIIME软件（v1.8.0，http://qiime.org/）调用USEARCH（v5.2.236, http://www.drive5.com/usearch/）检查并剔除嵌合体序列。

具体统计结果见下表。

#### 结果说明

通过上述步骤剔除疑问序列后，对每个样本的测序量进行统计，结果如下表所示。

**每样本测序量统计表**

| 样本名 | 测序量 |
| --- | --- |
| A01 | 63272 |
| A02 | 63484 |
| A03 | 68654 |
| A04 | 65000 |
| A05 | 63978 |
| A06 | 63857 |
| A07 | 64172 |
| A08 | 64708 |
| A09 | 58044 |
| A10 | 74996 |
| A11 | 75810 |
| A12 | 101891 |
| A13 | 71818 |
| B01 | 59110 |
| B02 | 55475 |
| B03 | 85133 |
| B04 | 83513 |
| B05 | 70419 |
| B06 | 70813 |
| B07 | 73599 |
| B08 | 78563 |
| B09 | 76369 |
| B10 | 91461 |
| total | 1644139 |

注：表中第一列为样本名；第二列为通过质量筛查、且Index完全匹配的有效序列量。

---

### 序列长度分布统计

#### 方法步骤 在R软件中编写脚本，对全部样本所包含的序列的长度分布进行统计。 结果说明 **序列长度分布图** 注：横坐标为全部样本中序列的长度分布，纵坐标则为各长度值所对应的序列总数。 结果目录: A02\_sequences --- OTU划分和分类地位鉴定 OTU划分 在微生物生态学领域，OTU（Operational Taxonomic Unit，可操作分类单元）(Blaxter et al., 2005)通常是指根据某一人为设定的序列相似度阈值，将来自一个或多个样本的序列进行归并，彼此间相似度高于该阈值的序列都将归并为一个OTU。通过序列归并和OTU划分（Demarcation），不仅可以简化数据结构，也更有利于在某一确定的分类水平，对不同来源的微生物群落样本进行互相比较。目前大多数基于16S rRNA基因的菌群结构多样性研究中，通常都以97%的序列相似度作为OTU划分阈值，该阈值大致相当于分类学中物种（Species）水平的序列差异。 方法步骤 使用QIIME软件，调用UCLUST这一序列比对工具(Edgar, 2010)，对前述获得的序列按97%的序列相似度进行归并和OTU划分，并选取每个OTU中丰度最高的序列作为该OTU的代表序列。随后，根据每个OTU在每个样本中所包含的序列数，构建OTU在各样本中丰度的矩阵文件（即OTU table），该矩阵文件可转换为“BIOM（Biological Observation Matrix）”这一更便于传输、储存并兼容于其它分析工具的文件格式。 OTU分类地位鉴定 方法步骤 对于每个OTU的代表序列，在QIIME软件中使用默认参数，通过将OTU代表序列与对应数据库的模板序列相比对，获取每个OTU所对应的分类学信息。对于不同类别的序列，分别采用各自特定的数据库作为OTU分类地位鉴定的模板序列： - a) 针对细菌和古菌的16S rRNA基因数据库： 默认采用Greengenes数据库（Release 13.8，http://greengenes.secondgenome.com/）(DeSantis et al., 2006)，也可按需选择RDP（Ribosomal Database Project）数据库（Release 11.1，http://rdp.cme.msu.edu/）(Cole et al., 2009)或Silva数据库（Release115，http://www.arb-silva.de）(Quast et al., 2013)； - b) 针对真菌的18S rRNA基因数据库： 采用Silva数据库；（Release115，http://www.arb-silva.de）(Quast et al., 2013)； - c) 针对真菌的ITS序列的数据库： 采用UNITE数据库（Release 5.0，https://unite.ut.ee/）(Koljalg et al., 2013)。 值得注意的是，虽然理论上所有的微生物序列都应当能在种甚至菌株水平得到鉴定，但由于微生物种类繁多，目前上述常用数据库还很难包罗万象；加之测序读长的限制，因此，在实际分析过程中，并非所有OTU代表序列都能获得属或种水平的分类学信息（即在对应的分类学水平尚且属于“Unclassified”）。也总是有可能遇到某些较为新奇、尚未被充分研究的微生物，此为正常现象。 OTU精简和分类鉴定结果统计 **对于菌群数据，划分OTU获得的原始OTU丰度矩阵中，很可能包含大量丰度极低的OTU，这些OTU也往往仅在少数样本中偶尔出现（即出现频率低）；而高丰度的占优势的OTU则相对少得多。这一菌群组成分布的“两极分化”现象已被越来越多的研究所发现，而这些丰度和出现频率都非常低的“稀有”OTU相当于菌群数据中的“背景噪音”，极大地增加了数据分析的复杂度。通常情况下，去除这些稀有OTU对于解析整体菌群的影响微乎其微，却能显著改善菌群数据分析的效率（如果对稀有OTU感兴趣，建议对目标微生物单独进行富集，从而对它们的丰度和出现频率进行更精准的研究）。因此，为了保证分析结果的可靠准确，我们建议去除原始OTU丰度矩阵中包含的稀有OTU。** 方法步骤 将丰度值低于全体样本测序总量0.001%（十万分之一）的OTU去除(Bokulich et al., 2013)，并将去除了稀有OTU的此丰度矩阵用于后续的一系列分析。首先进行OTU划分并对分类地位鉴定结果进行统计，结果见下表；同时，也使用R软件将上述表中各样本在各分类水平的鉴定结果绘制成柱状图，以直观地比较不同样本的OTU数和分类地位鉴定结果的差异，如下图所示。 结果说明 **OTU划分和分类地位鉴定结果统计表** | Sample | Phylum | Class | Order | Family | Genus | Species | Unclassified | | --- | --- | --- | --- | --- | --- | --- | --- | | A01 | 260 | 260 | 256 | 240 | 145 | 22 | 1 | | A02 | 148 | 148 | 148 | 141 | 96 | 11 | 1 | | A03 | 471 | 469 | 460 | 399 | 228 | 31 | 3 | | A04 | 367 | 365 | 360 | 315 | 195 | 33 | 2 | | A05 | 154 | 154 | 153 | 142 | 94 | 13 | 1 | | A06 | 345 | 344 | 336 | 308 | 173 | 23 | 2 | | A07 | 302 | 302 | 300 | 259 | 156 | 22 | 0 | | A08 | 512 | 512 | 508 | 431 | 183 | 24 | 1 | | A09 | 271 | 270 | 267 | 234 | 151 | 24 | 0 | | A10 | 452 | 451 | 446 | 401 | 184 | 27 | 1 | | A11 | 542 | 540 | 536 | 465 | 217 | 28 | 2 | | A12 | 540 | 540 | 534 | 458 | 209 | 38 | 1 | | A13 | 218 | 216 | 214 | 199 | 142 | 19 | 1 | | B01 | 444 | 442 | 437 | 385 | 202 | 36 | 2 | | B02 | 338 | 338 | 331 | 283 | 175 | 32 | 1 | | B03 | 600 | 599 | 590 | 494 | 241 | 46 | 3 | | B04 | 390 | 389 | 382 | 352 | 201 | 36 | 1 | | B05 | 321 | 320 | 316 | 278 | 178 | 25 | 3 | | B06 | 204 | 204 | 199 | 180 | 119 | 21 | 0 | | B07 | 647 | 646 | 637 | 543 | 276 | 42 | 2 | | B08 | 945 | 944 | 908 | 695 | 274 | 42 | 1 | | B09 | 608 | 608 | 586 | 471 | 223 | 47 | 1 | | B10 | 288 | 288 | 286 | 263 | 159 | 19 | 0 | 注：“Phylum”、“Class”、“Order”、“Family”、“Genus”、“Species”分别对应各样本中能分类至门、纲、目、科、属、种的OTU数，而“Unclassified”指未能归属到任何已知分类单元的OTU数。 **OTU划分和分类地位鉴定结果统计图** 注：横坐标依据样本名排列，纵坐标为各样本中能分类至门、纲、目、科、属、种各分类水平的OTU数。 共有OTU分析 方法步骤 根据获得的OTU丰度矩阵，使用R软件计算各样本（组）共有OTU的数量，并通过Venn图（https://en.wikipedia.org/wiki/Venn\_diagram）直观地呈现各样本（组）所共有和独有OTU所占的比例。 结果说明 venn.illness-normal.png **共有OTU的Venn图** 注：每个椭圆代表一个（组）样本，椭圆间的重叠区域表明样本（组）间的共有OTU，每个区块的数字表明该区块所包含的样本（组）的共有或独有OTU数量。 **说明：我们默认对于样本/分组数≤5的情况绘制Venn图。这是因为，随着样本/分组数的增加，Venn图的复杂度也急剧增大，当样本/分组数=5时，考虑到5组样本间所有可能的共有和独有关系，这些关系可能在Venn图上形成超过30块分区，进一步增加样本/分组数将使得图片非常拥挤，复杂度急剧增加，而可读性大大降低。同时，纵观文献中报道发表的Venn图，也几乎没有超过5组样本绘制图形的情况。因此，我们不建议在样本/分组数≥5时绘制Venn图；如果样本/分组数较多，可以考虑挑选重要的组别进行绘制，或者通过绘制多张Venn图的方式实现相互比较。** 结果目录: B01\_OTU --- Alpha多样性分析 Rarefaction稀疏曲线 获得OTU丰度矩阵之后，可以进行一系列的分析，比如计算每个样本群落的多样性（即Alpha多样性）。首先，可以绘制稀疏曲线（Rarefaction curve），以此评判每个样本的当前测序深度是否足以反映该群落样本所包含的微生物多样性。稀疏曲线是生态学领域的一种常用方法，通过从每个样本中随机抽取一定数量的序列（即在不超过现有样本测序量的某个深度下进行重抽样），可以预测样本在一系列给定的测序深度下，所可能包含的物种总数及其中每个物种的相对丰度(Heck et al., 1975; Kemp and Aller, 2004)。因此，通过绘制稀疏曲线，还可以在相同的测序深度下，比较不同样本中OTU数的多少，从而在一定程度上衡量每个样本的多样性高低。 方法步骤 使用QIIME软件，对OTU丰度矩阵中每个样本的序列总数在不同深度下随机抽样，以每个深度下抽取到的序列数及其对应的OTU数绘制稀疏曲线。 结果说明 **OTU数的稀疏曲线图** 注：横坐标代表每个样本中随机抽取的序列总数；纵坐标代表在相应深度下观测到的OTU数。曲线的长短反映了样本测序量的高低，曲线越长，测序深度越高，观测到更高多样性的可能性更大。曲线的平缓程度反映了测序深度对于观测样本多样性的影响大小，曲线越平缓，表明测序结果已足够反映当前样本所包含的多样性，继续增加测序深度已无法检测到大量的尚未发现的新OTU；反之，则表明多样性尚未接近饱和，继续增加测序深度将有助于观测到更多的新OTU。 本报告对应稀疏曲线点击本链接 结果目录: B02\_arare Specaccum物种累积曲线 物种累积曲线（Species accumulation curves）与稀疏曲线类似，用于衡量和预测群落中物种丰富度随样本量扩大而增加的幅度，被广泛用于判断样本量是否足够并估计群落丰富度(Chao and Shen, 2004)。通过物种累积曲线，不仅可以估计样本量是否足以反映不同群落间的多样性差异，在样本量足够多的前提下，也可以大致估计群落多样性的上限（一般在样本量> 10个时进行分析）。 方法步骤 使用R软件，对OTU丰度矩阵中每个样本所对应的OTU总数绘制Specaccum物种累积曲线。 结果说明 **Specaccum物种累积曲线图** 注：横坐标代表样本量，纵坐标代表被检测的物种数，蓝色阴影反映了曲线的置信区间。结果反映了在对样本总体抽样的过程中，持续扩大样本量时所观察到的新物种的增加速率。一般而言，在样本量较少时，随着新样本的加入，将有较大可能性发现大量的新物种（如OTU），此时曲线将呈现急剧上升的形态；当样本量已经较大时，此时群落中的OTU总数将不再随着新样本的加入而显著增加，曲线也将趋于平缓。因此，可以利用物种累积曲线判断样本量是否足够大，曲线急剧上升表明样本量不足，需要扩大采样规模；反之，则表明样本量已足以反映群落的丰富度。 结果目录: B03\_specaccum 丰度等级曲线 与稀疏曲线不同，丰度等级曲线（Rank abundance curve）将每个样本中的OTU按其丰度大小沿横坐标依次排列，并以各自的丰度值为纵坐标，用折线或曲线将各OTU互相连接，从而反映各样本中OTU丰度的分布规律（详见https://en.wikipedia.org/wiki/Rank\_abundance\_curve）。对于微生物群落样本，该曲线可以直观地反映群落中高丰度和稀有OTU的数量。 方法步骤 将每个样本的OTU按其丰度从大到小沿横坐标依次排列后，将丰度值经Log2对数转换作为纵坐标，在R软件中编写脚本绘制各样本的丰度等级曲线。 结果说明 **丰度等级曲线图** 注：横坐标为按丰度大小排列的OTU，纵坐标代表每个OTU在该样本中的丰度。每条折线代表一个样本的OTU丰度分布，折线在横轴上的长度反映了该样本中OTU数的多少，代表了群落的丰富度（Richness），折线越长，该样本中的OTU数越多；折线的平缓程度则反映了群落组成的均匀度（Evenness），折线越平缓，群落组成的均匀度越高，折线越陡峭，则群落中各OTU间的丰度差异越大，均匀度越低。 结果目录: B04\_rabund Alpha多样性指数计算 对于微生物群落而言，有多种指数来反映其Alpha多样性。不同的指数对于衡量群落多样性的侧重点各不相同，有些更侧重于体现群落的丰富度（即群落中微生物成员如OTU的数量），有些更倾向于反映群落的均匀度（即各成员间的丰度差异大小），也有一些多样性指数则综合考虑了以上两方面的因素。 常用的度量指数主要包括侧重于体现群落丰富度Chao1指数和ACE指数，以及兼顾群落均匀度的Shannon指数和Simpson指数。 Chao1丰富度估计指数（The Chao1 estimator，http://scikit-bio.org/docs/latest/generated/generated/skbio.diversity.alpha.chao1.html）由Chao首先提出(Chao, 1984)，通过计算群落中只检测到1次和2次的OTU数（即“Singleton”和“Doubleton”），估计群落中实际存在的物种数。 ACE 丰富度估计指数（The ACE estimator，http://scikit-bio.org/docs/latest/generated/generated/skbio.diversity.alpha.ace.html）的计算方法更复杂，默认将序列量10以下的OTU都计算在内，从而估计群落中实际存在的物种数，同样由Chao提出(Chao and Yang, 1993)。 一般而言，Chao1或ACE指数越大，表明群落的丰富度越高。 与Chao1和ACE指数不同，Shannon多样性指数（Shannon diversity index，或称为Shannon-Wiener、Shannon-Weaver指数，http://scikit-bio.org/docs/latest/generated/generated/skbio.diversity.alpha.shannon.html）（Shannon, 1948a, b）综合考虑了群落的丰富度和均匀度。Shannon指数值越高，表明群落的多样性越高。 Simpson 多样性指数（The Simpson index，http://scikit-bio.org/docs/latest/generated/generated/skbio.diversity.alpha.simpson.html）也是评价群落多样性的常用指数之一，由Edward Hugh Simpson提出(Simpson, 1949)。Simpson指数值越高，表明群落多样性越高。 一般而言，Shannon指数对群落的丰富度以及稀有OTU更敏感，而Simpson指数对均匀度和群落中的优势OTU更敏感。 方法步骤 **原始数据中，不同样本的测序量往往不一致，因此，在进行数据分析时，需要考虑不同样本测序量的差异。目前最常用的校正测序深度的方法是，对OTU丰度矩阵中的全体样本，根据最低测序深度统一进行随机重抽样，也就是所谓的“拉平处理”，从而在一致的测序深度获得稀疏化（Rarefied）OTU丰度矩阵。同根据测序量直接换算为相对丰度百分比的方法相比，拉平处理可以更好地避免测序深度导致的样本间差异，从而更为客观地反映不同样本间菌群的Alpha和Beta多样性差异。因此，我们在Alpha和Beta多样性分析时，都是对经过拉平处理的数据进行分析，从而最大程度上保证分析的一致性和可靠性。** **为了比较不同样本的多样性，首先对OTU丰度矩阵中的全体样本在90%的最低测序深度水平,统一进行随机重抽样（即“序列量拉平处理”），从而校正测序深度引起的多样性差异。随后，使用QIIME软件分别对每个样本计算上述四种多样性指数。** 结果说明 **菌群微生物多样性指数表** | | simpson | chao1 | ACE | shannon | | --- | --- | --- | --- | --- | | A01 | 0.91 | 261.77 | 265.49 | 4.38 | | A02 | 0.93 | 149.00 | 149.00 | 4.59 | | A03 | 0.95 | 482.75 | 480.80 | 5.90 | | A04 | 0.94 | 370.70 | 374.04 | 5.39 | | A05 | 0.87 | 155.00 | 155.00 | 3.85 | | A06 | 0.94 | 348.70 | 351.28 | 5.18 | | A07 | 0.96 | 303.36 | 307.88 | 5.24 | | A08 | 0.95 | 513.60 | 517.27 | 5.35 | | A09 | 0.93 | 271.00 | 271.00 | 4.92 | | A10 | 0.92 | 491.50 | 500.08 | 4.79 | | A11 | 0.96 | 562.14 | 559.02 | 5.92 | | A12 | 0.96 | 560.22 | 568.31 | 5.46 | | A13 | 0.94 | 231.69 | 236.25 | 4.89 | | B01 | 0.97 | 446.00 | 446.00 | 6.19 | | B02 | 0.93 | 339.00 | 339.00 | 5.31 | | B03 | 0.96 | 634.79 | 629.18 | 6.62 | | B04 | 0.93 | 402.48 | 406.38 | 5.20 | | B05 | 0.95 | 334.12 | 333.43 | 5.33 | | B06 | 0.90 | 206.40 | 206.96 | 4.27 | | B07 | 0.93 | 674.97 | 682.81 | 5.41 | | B08 | 0.98 | 970.67 | 956.33 | 7.43 | | B09 | 0.96 | 638.01 | 660.34 | 5.91 | | B10 | 0.84 | 303.92 | 312.85 | 3.85 | 注：表中第一列为样本名，之后各列分别对应每个样本在相同测序深度下的Chao1、ACE、Shannon、Simpson等多样性指数计算结果。 结果目录: B05\_index 分类学组成分析 各分类水平的微生物类群数统计 方法步骤 根据OTU划分和分类地位鉴定结果，可以获得每个样本在各分类水平的具体组成。由门、纲、目、科、属、种组成的不同分类水平，相当于以不同的分辨率查看群落组成结构。首先，可以比较不同样本在各分类水平所含有的微生物类群数量，如下表所示；也可以使用R软件将上述表中数据绘制成柱状图，以直观地比较不同样本在同一水平的分类单元数的差异。 结果说明 **各分类水平的微生物类群数统计表** | Sample | Phylum | Class | Order | Family | Genus | Species | | --- | --- | --- | --- | --- | --- | --- | | A01 | 13 | 24 | 36 | 74 | 89 | 22 | | A02 | 10 | 18 | 26 | 49 | 55 | 11 | | A03 | 25 | 50 | 70 | 103 | 118 | 29 | | A04 | 22 | 37 | 51 | 85 | 102 | 31 | | A05 | 11 | 21 | 31 | 56 | 60 | 13 | | A06 | 20 | 34 | 46 | 79 | 101 | 22 | | A07 | 16 | 29 | 44 | 71 | 86 | 21 | | A08 | 15 | 30 | 46 | 84 | 93 | 23 | | A09 | 16 | 31 | 44 | 77 | 93 | 24 | | A10 | 21 | 34 | 47 | 84 | 96 | 26 | | A11 | 22 | 38 | 58 | 93 | 109 | 26 | | A12 | 22 | 42 | 61 | 97 | 116 | 35 | | A13 | 12 | 23 | 33 | 67 | 77 | 19 | | B01 | 20 | 35 | 56 | 84 | 105 | 31 | | B02 | 19 | 38 | 56 | 84 | 98 | 30 | | B03 | 25 | 44 | 65 | 101 | 128 | 44 | | B04 | 19 | 34 | 52 | 90 | 113 | 31 | | B05 | 19 | 37 | 55 | 87 | 99 | 25 | | B06 | 14 | 25 | 37 | 67 | 70 | 18 | | B07 | 21 | 38 | 53 | 98 | 130 | 36 | | B08 | 26 | 61 | 89 | 128 | 136 | 40 | | B09 | 23 | 54 | 77 | 110 | 113 | 40 | | B10 | 19 | 31 | 46 | 71 | 81 | 19 | 注：“Phylum”、“Class”、“Order”、“Family”、“Genus”、“Species”分别对应各样本在门、纲、目、科、属、种各分类水平的微生物类群数。 **各分类水平的微生物类群数统计图** 注：横坐标依据样本名排列，纵坐标为门、纲、目、科、属、种六个分类水平各自含有的微生物类群数。 结果目录: B06\_taxa 各分类水平的分类学组成分析 方法步骤 使用QIIME软件，获取各样本在门、纲、目、科、属五个分类水平上的组成和丰度分布表，并通过饼图、柱状图或面积图呈现分析结果。根据研究对象是单个或多个群落样本，绘图结果可能会以不同方式进行展示。 结果说明 **各水平的群落分类学组成和丰度分布图** 注：横坐标依据样本名排列，每一个柱形图代表一个样本，并以颜色区分各分类单元，纵坐标代表各分类单元的相对丰度，柱子越长，该分类单元在对应样本中的相对丰度越高。 也可以使用R软件，对关注的特定样本在特定分类水平的组成绘制柱状图，如下图所示。 phylum.png class.png order.png family.png genus.png **菌群分类学组成和分布图** 注：横坐标依据样本名排列，每一个柱形图代表一个样本，并以颜色区分各分类单元，纵坐标代表各分类单元的相对丰度，柱子越长，该分类单元在对应样本中的相对丰度越高。 结果目录: B07\_taxa\_summary 样本（组）间分类学组成的差异分析 根据每个样本在各分类学水平的组成和序列分布，可以逐一比较每个分类单元在两个或多个样本（组）之间的丰度差异，并通过统计检验评价差异是否显著。 Metastats分析 方法步骤 使用Mothur软件，调用Metastats（http://metastats.cbcb.umd.edu/）(White et al., 2009)的统计学算法，对门和属水平的各个分类单元在样本（组）之间的序列量（即绝对丰度）差异进行两两比较检验。 结果说明 **样本（组）之间的Metastats两两比较检验结果统计表** | Group | Phylum | Genus | | --- | --- | --- | | illness-normal | 4 | 51 | 注：表中第一列为彼此两两比较的样本（组），“Phylum”和“Genus”分别对应每次比较发现的门和属水平有显著差异的分类单元个数。 每一次Metastats两两比较检验样本（组），都将获得对应的P值和Q值。一般而言，P值需< 0.05，P值越接近于零，则统计检验的效力越强，样本（组）之间具有统计学显著性的差异越大。而P值会因多重比较检验而产生误差（相当于显著性水平变宽松），应当使用错误发现率（False discovery rate，FDR）对P值进行校正，校正后的P值称为Q值，Q值越接近于零，假阳性的概率越低，统计检验的效果显著性越强，样本（组）之间真实的统计学差异越大。 我们可以进一步通过下图呈现的各分类单元在各样本（组）内的丰度分布，对上述显著性差异分析结果进行解读。 diff\_group\_phylum.png diff\_group\_genus.png **样本（组）间差异最显著的前20个分类单元的丰度分布图** 注：图中横坐标为差异最显著的前20个分类单元，纵坐标为各分类单元在各样本（组）内的序列量。样本无分组情况下，将以散点图的形式展示；样本有分组的情况下，将以小提琴图结合箱线图的形式展示：其中，小提琴图可以直观地显示数据的分布特征，“小提琴”的“胖瘦”反映了样本数据分布的密度高低（宽度越宽，表明该序列量下对应的样本越多）；箱线图边框代表上下四分位数间距（Interquartile range，IQR），横线代表中位值，上下触须分别代表上下四分位以外的1.5倍IQR范围，符号“•”表示超过范围的极端值。该图可配合Metastats差异显著性分析结果解读。 结果目录: C01\_diff/metastats LEfSe分析 LEfSe为最近出现的一种基于线性判别分析（Linear discriminant analysis，LDA）效应量（Effect size）的分析方法，其本质是将线性判别分析与非参数的Kruskal-Wallis以及Wilcoxon秩和检验相结合，从而筛选关键的生物标记物（也就是关键群落成员）(Segata et al., 2011)。该方法由美国哈佛大学的Curtis Huttenhower课题组开发，它的一大特点是，不仅局限于对不同样本分组中的群落组成差异进行分析，更可以深入到不同的子分组（Subgroup）中，挑取在不同子分组中表现一致的关键微生物类群，因而目前已获得了广泛的应用。 分析步骤 通过Galaxy在线分析平台（http://huttenhower.sph.harvard.edu/galaxy/），提交属水平的相对丰度矩阵进行LEfSe分析。LEfSe能自动对各分类水平的组成进行统计分析，并将分析结果可视化。 结果说明 **LEfSe统计检验结果列表** | | | | | | | --- | --- | --- | --- | --- | | Bacteria.Firmicutes.Clostridia.Clostridiales.Eubacteriaceae | 1.63917543713 | | | - | | Bacteria.Actinobacteria.Actinobacteria.Actinomycetales.Micromonosporaceae | 2.51393894227 | | | - | | Bacteria.Lentisphaerae.\_Lentisphaeria\_.Z20.R4\_45B | 1.83301703377 | | | - | | Bacteria.Proteobacteria.Alphaproteobacteria.Rhizobiales.Brucellaceae.Ochrobactrum | 4.8853589646 | | | - | | Bacteria.Proteobacteria.Betaproteobacteria.Burkholderiales.Comamonadaceae.Curvibacter | 1.90702952015 | | | - | | Bacteria.Firmicutes.Clostridia.Clostridiales.Lachnospiraceae.Anaerostipes | 1.77373317128 | | | - | | Bacteria.Actinobacteria.Actinobacteria.Actinomycetales.Micrococcaceae.Arthrobacter | 3.24421841862 | | | - | | Bacteria.Proteobacteria.Betaproteobacteria.Neisseriales | 3.67975256469 | | | - | | Bacteria.Acidobacteria.Sva0725 | 1.44893864089 | | | - | | Bacteria.Firmicutes.Bacilli.Bacillales.Bacillaceae.Geobacillus | 2.90650601384 | | | - | | Bacteria.Firmicutes.Clostridia.Clostridiales.Dehalobacteriaceae | 1.5680102267 | | | - | | Bacteria.Actinobacteria.Actinobacteria.Actinomycetales.Pseudonocardiaceae | 3.7964022464 | | | - | | Bacteria.Acidobacteria.Solibacteres.Solibacterales.Solibacteraceae.Candidatus\_Solibacter | 1.90308177035 | | | - | | Bacteria.Bacteroidetes.Bacteroidia.Bacteroidales.Bacteroidaceae.Bacteroides | 3.07298654335 | | | - | | Bacteria.Bacteroidetes.Flavobacteriia.Flavobacteriales.Flavobacteriaceae.Robiginitalea | 2.09787776616 | | | - | | Bacteria.Proteobacteria.Alphaproteobacteria.Rhizobiales.Phyllobacteriaceae | 3.33744083961 | | | - | | Bacteria.Firmicutes.Bacilli.Bacillales.Bacillaceae.Bacillus | 3.07183846228 | | | - | | Bacteria.Acidobacteria.Solibacteres | 2.88712709906 | | | - | | Bacteria.Proteobacteria.Alphaproteobacteria.Rhizobiales.Hyphomicrobiaceae.Rhodoplanes | 2.67006968962 | | | - | | Bacteria.Proteobacteria.Gammaproteobacteria.Enterobacteriales.Enterobacteriaceae.Edwardsiella | 1.81462785669 | | | - | | Bacteria.Bacteroidetes | 4.95890444927 | | | - | | Bacteria.Acidobacteria.Acidobacteria\_6.iii1\_15 | 2.93441751695 | | | - | | Bacteria.Nitrospirae.Nitrospira.Nitrospirales.Nitrospiraceae.Nitrospira | 1.8820247655 | | | - | | Bacteria.Proteobacteria.Deltaproteobacteria.Syntrophobacterales.Syntrophobacteraceae.Syntrophobacter | 1.72077045984 | | | - | | Bacteria.Firmicutes.Bacilli.Bacillales.Planococcaceae | 3.41037070124 | | | - | | Bacteria.Proteobacteria.Gammaproteobacteria.Pseudomonadales.Moraxellaceae | 4.30381410586 | | | - | | Bacteria.Firmicutes.Clostridia.Clostridiales.Ruminococcaceae | 4.32412979658 | | | - | | Bacteria.Firmicutes.Bacilli.Lactobacillales.Lactobacillaceae.Lactobacillus | 3.63994172475 | | | - | | Bacteria.Bacteroidetes.Flavobacteriia.Flavobacteriales.Flavobacteriaceae | 2.8378681582 | | | - | | Bacteria.Fusobacteria.Fusobacteriia.Fusobacteriales.Fusobacteriaceae.Cetobacterium | 3.56747895677 | | | - | | Bacteria.Bacteroidetes.Cytophagia.Cytophagales.Cytophagaceae | 3.06043501663 | | | - | | Bacteria.Firmicutes.Erysipelotrichi.Erysipelotrichales | 3.31548205546 | | | - | | Bacteria.Actinobacteria.Actinobacteria.Actinomycetales.Corynebacteriaceae | 4.61869972568 | | | - | | Bacteria.Proteobacteria.Epsilonproteobacteria.Campylobacterales.Campylobacteraceae | 5.02492739471 | | | - | | Bacteria.Gemmatimonadetes.Gemmatimonadetes.KD8\_87 | 1.3346441876 | | | - | | Bacteria.Firmicutes.Clostridia.Thermoanaerobacterales.Thermoanaerobacteraceae.Thermoanaerobacter | 1.69657050687 | | | - | | Bacteria.Proteobacteria.Betaproteobacteria.Burkholderiales.Comamonadaceae.Rhodoferax | 1.33540502856 | | | - | | Bacteria.Bacteroidetes.Sphingobacteriia.Sphingobacteriales.Sphingobacteriaceae.Sphingobacterium | 1.48851502859 | | | - | | Bacteria.Verrucomicrobia.Opitutae | 1.44858753991 | | | - | | Bacteria.OD1 | 2.06616536604 | | | - | | Bacteria.Proteobacteria.Gammaproteobacteria.Enterobacteriales | 4.15948209572 | | | - | | Bacteria.TM7.TM7\_3 | 2.75282848673 | | | - | | Bacteria.Proteobacteria.Gammaproteobacteria.Pseudomonadales.Moraxellaceae.Acinetobacter | 4.06703654393 | | | - | | Bacteria.Firmicutes.Clostridia.Clostridiales.\_Tissierellaceae\_.Anaerococcus | 4.14111300362 | | | - | | Bacteria.Firmicutes.Clostridia.Thermoanaerobacterales | 1.69657050687 | | | - | | Bacteria.Actinobacteria.Thermoleophilia.Solirubrobacterales.Solirubrobacteraceae | 1.48461464359 | | | - | | Bacteria.Firmicutes.Clostridia.Clostridiales.Ruminococcaceae.Oscillospira | 3.00089875354 | | | - | | Bacteria.Actinobacteria.Actinobacteria.Actinomycetales.Nocardiaceae.Rhodococcus | 3.39623400578 | | | - | | Bacteria.Proteobacteria.Alphaproteobacteria.Rhizobiales | 4.96859688534 | | | - | | Bacteria.Acidobacteria.Acidobacteria\_6 | 2.97903240527 | | | - | | Bacteria.Planctomycetes.Phycisphaerae | 2.91543423947 | | | - | | Bacteria.Proteobacteria.Alphaproteobacteria.Rhodospirillales.Acetobacteraceae | 2.82107611349 | | | - | | Bacteria.Actinobacteria.Actinobacteria.Actinomycetales.Bogoriellaceae | 1.57689434885 | | | - | | Bacteria.Proteobacteria.Deltaproteobacteria.Desulfovibrionales | 3.49864984251 | | | - | | Bacteria.Armatimonadetes.Chthonomonadetes.Chthonomonadales.Chthonomonadaceae | 2.04143196548 | | | - | | Bacteria.Actinobacteria.Actinobacteria.Actinomycetales.Actinosynnemataceae.Lentzea | 1.68385967415 | | | - | | Bacteria.WPS\_2 | 2.00095451173 | | | - | | Bacteria.\_Thermi\_.Deinococci | 2.87022518983 | | | - | | Bacteria.Proteobacteria.Gammaproteobacteria.Xanthomonadales.Xanthomonadaceae.Thermomonas | 1.54368097098 | | | - | | Bacteria.Firmicutes.Bacilli.Bacillales.Bacillaceae.Anoxybacillus | 2.60392856736 | | | - | | Bacteria.Proteobacteria.Epsilonproteobacteria | 5.02508479325 | | | - | | Bacteria.Chlamydiae.Chlamydiia.Chlamydiales.Rhabdochlamydiaceae | 2.173278372 | | | - | | Bacteria.Actinobacteria.Actinobacteria.Actinomycetales.Propionibacteriaceae.Brooklawnia | 1.90990194729 | | | - | | Bacteria.Armatimonadetes.\_Fimbriimonadia\_.\_Fimbriimonadales\_ | 2.06862190152 | | | - | | Bacteria.Cyanobacteria | 3.88353134976 | | | - | | Bacteria.Proteobacteria.Betaproteobacteria.Burkholderiales.Burkholderiaceae | 1.98971945885 | | | - | | Bacteria.Chlamydiae.Chlamydiia | 2.173278372 | | | - | | Bacteria.Proteobacteria.Gammaproteobacteria.Pasteurellales.Pasteurellaceae.Actinobacillus | 3.03661703564 | | | - | | Bacteria.Firmicutes.Bacilli.Lactobacillales.Aerococcaceae.Facklamia | 4.78803687037 | | | - | | Bacteria.Bacteroidetes.Bacteroidia.Bacteroidales.RF16 | 2.50456765866 | | | - | | Bacteria.Proteobacteria.Gammaproteobacteria.Enterobacteriales.Enterobacteriaceae.Serratia | 2.43541593402 | | | - | | Bacteria.Actinobacteria.Actinobacteria.Actinomycetales.Bogoriellaceae.Georgenia | 1.57689434885 | | | - | | Bacteria.Verrucomicrobia.Opitutae.\_Cerasicoccales\_.\_Cerasicoccaceae\_ | 1.44858753991 | | | - | | Bacteria.Actinobacteria.Actinobacteria | 4.87767770143 | | | - | | Bacteria.Deferribacteres.Deferribacteres.Deferribacterales.Deferribacteraceae | 1.76534317471 | | | - | | Bacteria.Actinobacteria.Actinobacteria.Actinomycetales.Intrasporangiaceae | 1.68115590039 | | | - | | Bacteria.\_Thermi\_.Deinococci.Deinococcales.Deinococcaceae | 2.83790793463 | | | - | | Bacteria.Actinobacteria.Actinobacteria.Actinomycetales.Micromonosporaceae.Dactylosporangium | 1.23773417459 | | | - | | Bacteria.Proteobacteria.Alphaproteobacteria.Rhodobacterales.Rhodobacteraceae.Rubellimicrobium | 1.5921446434 | | | - | | Bacteria.Firmicutes.Clostridia.Clostridiales.\_Tissierellaceae\_.Tissierella\_Soehngenia | 2.37853253993 | | | - | | Bacteria.Firmicutes.Clostridia.Clostridiales.Clostridiaceae.02d06 | 1.33540502856 | | | - | | Bacteria.Proteobacteria.Alphaproteobacteria.Rhizobiales.Methylobacteriaceae.Methylobacterium | 3.3686624778 | | | - | | Bacteria.Firmicutes.Erysipelotrichi.Erysipelotrichales.Erysipelotrichaceae.L7A\_E11 | 1.86911114875 | | | - | | Bacteria.Proteobacteria.Betaproteobacteria.MND1 | 2.45962742461 | | | - | | Bacteria.Proteobacteria.Alphaproteobacteria.Rhizobiales.Hyphomicrobiaceae.Devosia | 1.72118388175 | | | - | | Bacteria.Chloroflexi.Anaerolineae.SBR1031.oc28 | 1.93915884737 | | | - | | Bacteria.\_Thermi\_.Deinococci.Thermales.Thermaceae.Thermus | 1.93681648739 | | | - | | Bacteria.Lentisphaerae.\_Lentisphaeria\_.Victivallales.Victivallaceae | 3.05478775114 | | | - | | Bacteria.Proteobacteria.Alphaproteobacteria.Rhodobacterales.Rhodobacteraceae.Paracoccus | 2.02117686202 | | | - | | Bacteria.Bacteroidetes.Bacteroidia.Bacteroidales.S24\_7 | 3.27721553491 | | | - | | Bacteria.Cyanobacteria.Oscillatoriophycideae | 1.47673418135 | | | - | | Bacteria.Proteobacteria.Alphaproteobacteria.Sphingomonadales.Sphingomonadaceae.Sphingomonas | 4.04409855365 | | | - | | Bacteria.Fusobacteria.Fusobacteriia.Fusobacteriales.Fusobacteriaceae | 4.8565716163 | | | - | | Bacteria.Bacteroidetes.Bacteroidia.Bacteroidales.Rikenellaceae | 3.56198303376 | | | - | | Bacteria.Proteobacteria.Gammaproteobacteria.Pseudomonadales.Pseudomonadaceae | 4.29850168373 | | | - | | Bacteria.Nitrospirae.Nitrospira.Nitrospirales | 2.56911655074 | | | - | | Bacteria.Proteobacteria.Deltaproteobacteria.MIZ46 | 2.16128445099 | | | - | | Bacteria.Proteobacteria.Gammaproteobacteria.Pseudomonadales.Pseudomonadaceae.Pseudomonas | 3.1951219749 | | | - | | Bacteria.Proteobacteria.Alphaproteobacteria.Sphingomonadales | 4.14103807774 | | | - | | Bacteria.Chloroflexi.Chloroflexi | 2.11455755207 | | | - | | Bacteria.Actinobacteria.Actinobacteria.Actinomycetales.Actinosynnemataceae | 1.83271550336 | | | - | | Bacteria.Proteobacteria.Gammaproteobacteria.Xanthomonadales.Xanthomonadaceae.Lysobacter | 2.16225896396 | | | - | | Bacteria.Proteobacteria.Betaproteobacteria.Neisseriales.Neisseriaceae | 3.67975256469 | | | - | | Bacteria.Proteobacteria.Alphaproteobacteria.Rhizobiales.Bradyrhizobiaceae.Balneimonas | 2.56011820942 | | | - | | Bacteria.Chloroflexi.Chloroflexi.\_Roseiflexales\_.\_Kouleothrixaceae\_ | 2.00807141689 | | | - | | Bacteria.Chloroflexi.Anaerolineae.Caldilineales.Caldilineaceae.Caldilinea | 1.7555325661 | | | - | | Bacteria.Proteobacteria.Gammaproteobacteria.Xanthomonadales.Xanthomonadaceae.Luteimonas | 2.35442532724 | | | - | | Bacteria.Proteobacteria.Deltaproteobacteria.Desulfobacterales.Desulfobulbaceae | 1.69784229851 | | | - | | Bacteria.Actinobacteria.Actinobacteria.Actinomycetales.Sporichthyaceae | 1.28888669704 | | | - | | Bacteria.Chloroflexi.Chloroflexi.\_Roseiflexales\_ | 2.00807141689 | | | - | | Bacteria.Bacteroidetes.Bacteroidia.Bacteroidales.\_Odoribacteraceae\_.Odoribacter | 1.54019773569 | | | - | | Bacteria.Planctomycetes.Phycisphaerae.Phycisphaerales | 2.91543423947 | | | - | | Bacteria.Proteobacteria.Alphaproteobacteria.Rhodospirillales.Rhodospirillaceae.Skermanella | 1.50542953068 | | | - | | Bacteria.Acidobacteria.\_Chloracidobacteria\_ | 2.32205276984 | | | - | | Bacteria.Firmicutes.Clostridia.Clostridiales.\_Mogibacteriaceae\_ | 2.83971032594 | | | - | | Bacteria.Proteobacteria.Gammaproteobacteria.Alteromonadales.\_Chromatiaceae\_.Rheinheimera | 1.8295148352 | | | - | | Bacteria.Proteobacteria.Betaproteobacteria.Burkholderiales.Comamonadaceae.Delftia | 3.58520833622 | | | - | | Bacteria.Firmicutes.Bacilli.Lactobacillales.Streptococcaceae.Streptococcus | 3.74185167533 | | | - | | Bacteria.Actinobacteria.Rubrobacteria.Rubrobacterales.Rubrobacteraceae | 2.46569128599 | | | - | | Bacteria.Acidobacteria.Acidobacteriia.Acidobacteriales.Koribacteraceae | 2.35836521613 | | | - | | Bacteria.WS3 | 2.2191228881 | | | - | | Bacteria.Gemmatimonadetes.Gemmatimonadetes.N1423WL | 2.3803067349 | | | - | | Bacteria.Lentisphaerae.\_Lentisphaeria\_.Victivallales | 3.05478775114 | | | - | | Bacteria.Armatimonadetes.Chthonomonadetes | 2.04143196548 | | | - | | Bacteria.Acidobacteria.DA052 | 1.7816287422 | | | - | | Bacteria.Planctomycetes.OM190.agg27 | 1.6137581252 | | | - | | Bacteria.Proteobacteria.Gammaproteobacteria.\_Marinicellales\_.\_Marinicellaceae\_ | 1.63853659763 | | | - | | Bacteria.Acidobacteria.BPC102 | 1.33540502856 | | | - | | Bacteria.Bacteroidetes.Flavobacteriia.Flavobacteriales.\_Weeksellaceae\_ | 2.98042269807 | | | - | | Bacteria.Cyanobacteria.Chloroplast | 2.99375284908 | | | - | | Bacteria.Firmicutes.Clostridia.Clostridiales.Veillonellaceae.Sporomusa | 1.81462785669 | | | - | | Bacteria.Proteobacteria.Betaproteobacteria.Burkholderiales.Oxalobacteraceae.Oxalobacter | 1.51073544665 | | | - | | Bacteria.Proteobacteria.Gammaproteobacteria.Pseudomonadales | 4.50903996973 | | | - | | Bacteria.Firmicutes.Bacilli.Turicibacterales.Turicibacteraceae | 2.51314395699 | | | - | | Bacteria.Firmicutes.Clostridia.Clostridiales.\_Tissierellaceae\_.ph2 | 3.97329222961 | | | - | | Bacteria.Armatimonadetes.\_Fimbriimonadia\_ | 2.06862190152 | | | - | | Bacteria.Actinobacteria.Actinobacteria.Actinomycetales.Dietziaceae | 2.36120512487 | | | - | | Bacteria.Actinobacteria.Actinobacteria.Actinomycetales.Actinomycetaceae.Trueperella | 3.78448660036 | | | - | | Bacteria.Actinobacteria.Actinobacteria.Actinomycetales | 4.87672683349 | | | - | | Bacteria.Acidobacteria.Acidobacteria\_6.iii1\_15.mb2424 | 1.51088762526 | | | - | | Bacteria.Proteobacteria.Alphaproteobacteria.Rhizobiales.Xanthobacteraceae | 2.04670058453 | | | - | | Bacteria.Bacteroidetes.Bacteroidia.Bacteroidales.\_Paraprevotellaceae\_.YRC22 | 1.45827319751 | | | - | | Bacteria.Actinobacteria.Actinobacteria.Actinomycetales.Microbacteriaceae.Microbacterium | 2.87882463366 | | | - | | Bacteria.Firmicutes.Clostridia.Clostridiales.Lachnospiraceae.Dorea | 3.38061448612 | | | - | | Bacteria.Proteobacteria.Epsilonproteobacteria.Campylobacterales.Helicobacteraceae.Helicobacter | 1.54467687332 | | | - | | Bacteria.Firmicutes.Clostridia.Clostridiales.Christensenellaceae | 3.08916226255 | | | - | | Bacteria.Actinobacteria.Coriobacteriia.Coriobacteriales.Coriobacteriaceae.Adlercreutzia | 1.94038301134 | | | - | | Bacteria.Bacteroidetes.Bacteroidia.Bacteroidales.BA008 | 1.45191059763 | | | - | | Bacteria.Spirochaetes | 2.81568285925 | | | - | | Bacteria.Elusimicrobia.Elusimicrobia | 1.77497431319 | | | - | | Bacteria.Proteobacteria.Betaproteobacteria.Burkholderiales.Comamonadaceae | 4.20890797507 | | | - | | Bacteria.Actinobacteria.Actinobacteria.Bifidobacteriales.Bifidobacteriaceae.Bifidobacterium | 3.80908859425 | | | - | | Bacteria.Proteobacteria.Epsilonproteobacteria.Campylobacterales.Helicobacteraceae | 1.76871045252 | | | - | | Bacteria.Proteobacteria.Betaproteobacteria.Rhodocyclales.Rhodocyclaceae.KD1\_23 | 1.13465514588 | | | - | | Bacteria.Gemmatimonadetes.Gemm\_5 | 1.8413609532 | | | - | | Bacteria.Firmicutes.Bacilli.Bacillales.Bacillaceae | 3.40776962558 | | | - | | Bacteria.Firmicutes.Clostridia.Clostridiales.Peptococcaceae.Peptococcus | 3.17818849932 | | | - | | Bacteria.Firmicutes.Bacilli.Bacillales.Planococcaceae.Solibacillus | 1.85907315801 | | | - | | Bacteria.Acidobacteria.BPC102.MVS\_40 | 1.33540502856 | | | - | | Bacteria.Proteobacteria.Betaproteobacteria.IS\_44 | 2.0094223152 | | | - | | Bacteria.Proteobacteria.Betaproteobacteria.Hydrogenophilales.Hydrogenophilaceae.Thiobacillus | 2.23178460753 | | | - | | Bacteria.Gemmatimonadetes.Gemm\_1 | 3.03726440207 | | | - | | Bacteria.Firmicutes.Bacilli.Lactobacillales.Enterococcaceae | 2.36830690363 | | | - | | Bacteria.Proteobacteria.Gammaproteobacteria.Oceanospirillales.Halomonadaceae | 1.43191566869 | | | - | | Bacteria.Chloroflexi.Thermomicrobia | 2.16270033213 | | | - | | Bacteria.Proteobacteria.Alphaproteobacteria.Rhodospirillales.Acetobacteraceae.Roseomonas | 1.90783910492 | | | - | | Bacteria.Spirochaetes.Spirochaetes.Spirochaetales.Spirochaetaceae.Treponema | 2.78088827854 | | | - | | Bacteria.Proteobacteria.Betaproteobacteria.Burkholderiales.Oxalobacteraceae.Polynucleobacter | 1.63892628151 | | | - | | Bacteria.Chloroflexi.Anaerolineae.Caldilineales.Caldilineaceae | 1.7555325661 | | | - | | Bacteria.Nitrospirae.Nitrospira | 2.56911655074 | | | - | | Bacteria.Proteobacteria.Deltaproteobacteria.NB1\_j.NB1\_i | 2.11499164115 | | | - | | Bacteria.Spirochaetes.Spirochaetes.Spirochaetales | 2.81568285925 | | | - | | Bacteria.Proteobacteria.Gammaproteobacteria.Xanthomonadales.Xanthomonadaceae.Dyella | 1.54297867377 | | | - | | Bacteria.Verrucomicrobia | 3.46946297063 | | | - | | Bacteria.Tenericutes.Mollicutes.Acholeplasmatales | 3.10559509153 | | | - | | Bacteria.Proteobacteria.Alphaproteobacteria.RF32 | 1.64428416046 | | | - | | Bacteria.Proteobacteria.Betaproteobacteria.Burkholderiales.Comamonadaceae.Methylibium | 1.95131264195 | | | - | | Bacteria.Deferribacteres | 1.76534317471 | | | - | | Bacteria.Verrucomicrobia.Opitutae.\_Cerasicoccales\_ | 1.44858753991 | | | - | | Bacteria.Actinobacteria.Actinobacteria.Actinomycetales.Brevibacteriaceae.Brevibacterium | 2.78590003124 | | | - | | Bacteria.Proteobacteria.Betaproteobacteria.Hydrogenophilales.Hydrogenophilaceae | 2.23178460753 | | | - | | Bacteria.Planctomycetes.OM190 | 1.80359838424 | | | - | | Bacteria.Proteobacteria.Betaproteobacteria.Neisseriales.Neisseriaceae.Neisseria | 1.7407910962 | | | - | | Bacteria.\_Thermi\_ | 2.87022518983 | | | - | | Bacteria.Proteobacteria | 5.55428232528 | | | - | | Bacteria.Actinobacteria.Actinobacteria.Actinomycetales.Microbacteriaceae | 3.62687645831 | | | - | | Bacteria.Bacteroidetes.Bacteroidia.Bacteroidales.Bacteroidaceae | 3.52896132424 | | | - | | Bacteria.Actinobacteria.Actinobacteria.Actinomycetales.Micrococcaceae.Micrococcus | 2.0050692351 | | | - | | Bacteria.OD1.ABY1 | 1.22034435638 | | | - | | Bacteria.Actinobacteria.Acidimicrobiia.Acidimicrobiales.C111 | 1.79914245898 | | | - | | Bacteria.Chlamydiae.Chlamydiia.Chlamydiales | 2.173278372 | | | - | | Bacteria.Firmicutes.Clostridia.Clostridiales.\_Tissierellaceae\_.1\_68 | 4.21205937092 | | | - | | Bacteria.Proteobacteria.Alphaproteobacteria.Rhizobiales.Rhizobiaceae | 3.41300117459 | | | - | | Bacteria.Proteobacteria.Alphaproteobacteria.Rhodobacterales | 2.76940898525 | | | - | | Bacteria.Chloroflexi.Ktedonobacteria.JG30\_KF\_AS9 | 1.85815132074 | | | - | | Bacteria.Acidobacteria.Acidobacteriia.Acidobacteriales | 2.35836521613 | | | - | | Bacteria.Proteobacteria.Alphaproteobacteria.Sphingomonadales.Sphingomonadaceae.Kaistobacter | 2.19877301502 | | | - | | Bacteria.Proteobacteria.Alphaproteobacteria.Rhizobiales.Methylobacteriaceae | 3.88467214527 | | | - | | Bacteria.Proteobacteria.Alphaproteobacteria.Rhodobacterales.Hyphomonadaceae | 2.31674951415 | | | - | | Bacteria.Proteobacteria.Deltaproteobacteria.BPC076 | 1.36758971193 | | | - | | Bacteria.Actinobacteria.Rubrobacteria.Rubrobacterales.Rubrobacteraceae.Rubrobacter | 2.46569128599 | | | - | | Bacteria.Bacteroidetes.Cytophagia.Cytophagales.Cytophagaceae.Hymenobacter | 3.06043501663 | | | - | | Bacteria.Verrucomicrobia.Verruco\_5 | 3.06316674641 | | | - | | Bacteria.Proteobacteria.Alphaproteobacteria.Rhizobiales.Brucellaceae | 4.8853589646 | | | - | | Bacteria.Chloroflexi.Anaerolineae.Caldilineales | 1.7555325661 | | | - | | Bacteria.Proteobacteria.Gammaproteobacteria.Pasteurellales.Pasteurellaceae.Haemophilus | 3.54857211976 | | | - | | Bacteria.Fusobacteria | 5.23974633388 | | | - | | Bacteria.Firmicutes.Bacilli.Lactobacillales.Carnobacteriaceae.Granulicatella | 1.33673008541 | | | - | | Bacteria.Planctomycetes.C6 | 1.92570879462 | | | - | | Bacteria.GN02.3BR\_5F | 3.12288815943 | | | - | | Bacteria.Acidobacteria.Solibacteres.Solibacterales.Solibacteraceae | 1.90308177035 | | | - | | Bacteria.Proteobacteria.Gammaproteobacteria.Xanthomonadales.Xanthomonadaceae | 4.48939118631 | | | - | | Bacteria.Tenericutes.Mollicutes.RF39 | 2.32900345118 | | | - | | Bacteria.Tenericutes.RF3.ML615J\_28 | 2.22892822429 | | | - | | Bacteria.Cyanobacteria.4C0d\_2.YS2 | 2.62090792445 | | | - | | Bacteria.Bacteroidetes.Sphingobacteriia.Sphingobacteriales.Sphingobacteriaceae | 2.23373750228 | | | - | | Bacteria.Proteobacteria.Betaproteobacteria.Methylophilales | 1.65932576829 | | | - | | Bacteria.Proteobacteria.Gammaproteobacteria.Xanthomonadales | 4.50797341796 | | | - | | Bacteria.Actinobacteria.Actinobacteria.Actinomycetales.Nocardioidaceae.Propionicimonas | 1.45044771302 | | | - | | Bacteria.Proteobacteria.Deltaproteobacteria.Myxococcales.Myxococcaceae.Anaeromyxobacter | 1.61828436505 | | | - | | Bacteria.Firmicutes.Clostridia.Clostridiales.Lachnospiraceae.Roseburia | 2.13732814009 | | | - | | Bacteria.Proteobacteria.Alphaproteobacteria.Rhodobacterales.Rhodobacteraceae | 2.58054996758 | | | - | | Bacteria.Actinobacteria.Thermoleophilia.Solirubrobacterales | 2.02675688462 | | | - | | Bacteria.Actinobacteria.Actinobacteria.Actinomycetales.Actinomycetaceae.Actinomyces | 3.49285251783 | | | - | | Bacteria.Fusobacteria.Fusobacteriia.Fusobacteriales.Leptotrichiaceae | 5.00776758366 | | | - | | Bacteria.Chloroflexi.Anaerolineae.SBR1031.A4b | 2.64814612581 | | | - | | Bacteria.AD3 | 2.14477171624 | | | - | | Bacteria.Acidobacteria.\_Chloracidobacteria\_.RB41 | 2.24383546413 | | | - | | Bacteria.Proteobacteria.Alphaproteobacteria.Rhizobiales.Beijerinckiaceae | 1.4880401987 | | | - | | Bacteria.Proteobacteria.Alphaproteobacteria | 5.07123356365 | | | - | | Bacteria.Proteobacteria.Alphaproteobacteria.Rhodospirillales.Rhodospirillaceae | 2.86555319155 | | | - | | Bacteria.Proteobacteria.Alphaproteobacteria.Rhizobiales.Hyphomicrobiaceae.Hyphomicrobium | 1.78143646566 | | | - | | Bacteria.Actinobacteria.Acidimicrobiia | 2.52173837048 | | | - | | Bacteria.WS3.PRR\_12.Sediment\_1.PRR\_10 | 1.81453274621 | | | - | | Bacteria.Proteobacteria.Gammaproteobacteria.Alteromonadales.\_Chromatiaceae\_ | 1.8295148352 | | | - | | Bacteria.Gemmatimonadetes.Gemmatimonadetes.Gemmatimonadales.Ellin5301 | 2.24321375673 | | | - | | Bacteria.Proteobacteria.Betaproteobacteria | 4.83903533201 | | | - | | Bacteria.Firmicutes.Clostridia.Clostridiales.Ruminococcaceae.Butyricicoccus | 1.06683347194 | | | - | | Bacteria.Actinobacteria.Actinobacteria.Actinomycetales.Microbacteriaceae.Cryocola | 3.53108988046 | | | - | | Bacteria.Elusimicrobia.Elusimicrobia.Elusimicrobiales.Elusimicrobiaceae | 1.52232611956 | | | - | | Bacteria.Chloroflexi.Anaerolineae.S0208 | 1.61839098181 | | | - | | Bacteria.Firmicutes.Clostridia.Clostridiales.Lachnospiraceae.Butyrivibrio | 1.93780865604 | | | - | | Bacteria.Proteobacteria.Alphaproteobacteria.Caulobacterales.Caulobacteraceae | 3.92284851732 | | | - | | Bacteria.Bacteroidetes.Bacteroidia.Bacteroidales.\_Paraprevotellaceae\_.CF231 | 2.24575410389 | | | - | | Bacteria.Proteobacteria.Alphaproteobacteria.Rhizobiales.Xanthobacteraceae.Labrys | 1.70108788135 | | | - | | Bacteria.Proteobacteria.Betaproteobacteria.Ellin6067 | 2.12016111994 | | | - | | Bacteria.Firmicutes.Clostridia.Clostridiales.Veillonellaceae.Dialister | 3.3239035349 | | | - | | Bacteria.Proteobacteria.Deltaproteobacteria.Myxococcales.OM27 | 1.69657050687 | | | - | | Bacteria.Actinobacteria.Rubrobacteria.Rubrobacterales | 2.46569128599 | | | - | | Bacteria.Actinobacteria.Thermoleophilia.Gaiellales.Gaiellaceae | 2.17102200139 | | | - | | Bacteria.Armatimonadetes | 2.24703141349 | | | - | | Bacteria.Firmicutes | 5.60229119208 | | | - | | Bacteria.Proteobacteria.Gammaproteobacteria.Vibrionales | 1.65282544041 | | | - | | Bacteria.Armatimonadetes.0319\_6E2 | 1.3346441876 | | | - | | Bacteria.Firmicutes.Clostridia.Clostridiales.Lachnospiraceae.Blautia | 3.48037284573 | | | - | | Bacteria.Actinobacteria.Actinobacteria.Actinomycetales.Micromonosporaceae.Phytohabitans | 1.56820455817 | | | - | | Bacteria.Actinobacteria.Actinobacteria.Actinomycetales.Dermabacteraceae | 1.97298265476 | | | - | | Bacteria.GAL15 | 1.95029045869 | | | - | | Bacteria.Chloroflexi.S085 | 2.03646346246 | | | - | | Bacteria.Firmicutes.Bacilli.Bacillales.Planococcaceae.Planococcus | 2.47273121791 | | | - | | Bacteria.Chlamydiae.Chlamydiia.Chlamydiales.Rhabdochlamydiaceae.Candidatus\_Rhabdochlamydia | 2.173278372 | | | - | | Bacteria.Proteobacteria.Alphaproteobacteria.Rhodospirillales.Rhodospirillaceae.Telmatospirillum | 1.91984732276 | | | - | | Bacteria.Acidobacteria.Solibacteres.Solibacterales.PAUC26f | 1.63880119239 | | | - | | Bacteria.Proteobacteria.Deltaproteobacteria.Myxococcales.Cystobacterineae | 1.37603687276 | | | - | | Bacteria.Firmicutes.Clostridia.Clostridiales.Lachnospiraceae | 4.14838092433 | | | - | | Bacteria.Fusobacteria.Fusobacteriia.Fusobacteriales.Fusobacteriaceae.u114 | 1.30064292229 | | | - | | Bacteria.Proteobacteria.Gammaproteobacteria.Alteromonadales | 1.96253590511 | | | - | | Bacteria.Fibrobacteres.Fibrobacteria.Fibrobacterales | 1.47766715995 | | | - | | Bacteria.Proteobacteria.Gammaproteobacteria.Xanthomonadales.Sinobacteraceae | 3.13002898847 | | | - | | Bacteria.\_Thermi\_.Deinococci.Thermales | 1.93681648739 | | | - | | Bacteria.Firmicutes.Bacilli.Bacillales.\_Thermicanaceae\_.Thermicanus | 1.51073544665 | | | - | | Bacteria.Proteobacteria.Epsilonproteobacteria.Campylobacterales.Campylobacteraceae.Sulfurospirillum | 2.59154793233 | | | - | | Bacteria.Firmicutes.Clostridia.Clostridiales.\_Acidaminobacteraceae\_.Fusibacter | 4.1215622029 | | | - | | Bacteria.Proteobacteria.Deltaproteobacteria.Myxococcales.Haliangiaceae | 2.17528199633 | | | - | | Bacteria.Chlamydiae | 2.173278372 | | | - | | Bacteria.Bacteroidetes.Flavobacteriia | 3.16970947498 | | | - | | Bacteria.Gemmatimonadetes.Gemmatimonadetes.C114 | 1.44858753991 | | | - | | Bacteria.Actinobacteria.Actinobacteria.Actinomycetales.Actinosynnemataceae.Saccharothrix | 1.67495371737 | | | - | | Bacteria.Actinobacteria.Actinobacteria.Actinomycetales.Actinomycetaceae | 4.19878515257 | | | - | | Bacteria.Proteobacteria.Alphaproteobacteria.Rhizobiales.Methylocystaceae | 1.36647239206 | | | - | | Bacteria.Deferribacteres.Deferribacteres | 1.76534317471 | | | - | | Bacteria.Actinobacteria.Actinobacteria.Bifidobacteriales | 3.80908859425 | | | - | | Bacteria.Proteobacteria.Alphaproteobacteria.Rhizobiales.Beijerinckiaceae.Beijerinckia | 1.4880401987 | | | - | | Bacteria.TM7.TM7\_3.CW040.F16 | 2.55661008936 | | | - | | Bacteria.Proteobacteria.Deltaproteobacteria.Myxococcales.Myxococcaceae | 2.97664375003 | | | - | | Bacteria.Bacteroidetes.Bacteroidia.Bacteroidales.Bacteroidaceae.BF311 | 1.7278732636 | | | - | | Bacteria.Bacteroidetes.\_Saprospirae\_ | 4.40461810347 | | | - | | Bacteria.Actinobacteria.Actinobacteria.Actinomycetales.Geodermatophilaceae.Geodermatophilus | 1.96380850853 | | | - | | Bacteria.Proteobacteria.Deltaproteobacteria.Desulfovibrionales.Desulfovibrionaceae.Desulfovibrio | 2.4683098197 | | | - | | Bacteria.Nitrospirae | 2.56911655074 | | | - | | Bacteria.Firmicutes.Bacilli.Bacillales.Planococcaceae.Lysinibacillus | 1.84517217491 | | | - | | Bacteria.Bacteroidetes.Flavobacteriia.Flavobacteriales.\_Weeksellaceae\_.Chryseobacterium | 2.00016884585 | | | - | | Bacteria | 6.0 | | | - | | Bacteria.Cyanobacteria.4C0d\_2 | 3.82360401235 | | | - | | Bacteria.Chloroflexi.Anaerolineae.SBR1031.SJA\_101 | 1.44818417765 | | | - | | Bacteria.Firmicutes.Erysipelotrichi.Erysipelotrichales.Erysipelotrichaceae | 3.31548205546 | | | - | | Bacteria.Gemmatimonadetes | 3.33569353528 | | | - | | Bacteria.Proteobacteria.Deltaproteobacteria | 3.82779168252 | | | - | | Bacteria.Bacteroidetes.Bacteroidia.Bacteroidales.\_Paraprevotellaceae\_.Paraprevotella | 1.71820124793 | | | - | | Bacteria.Actinobacteria.Actinobacteria.Actinomycetales.Micromonosporaceae.Micromonospora | 1.33614167031 | | | - | | Bacteria.Actinobacteria.Actinobacteria.Actinomycetales.Nocardioidaceae.Aeromicrobium | 2.88890134619 | | | - | | Bacteria.Acidobacteria.Acidobacteria\_6.iii1\_15.RB40 | 1.37603687276 | | | - | | Bacteria.Actinobacteria.Actinobacteria.Actinomycetales.Microbacteriaceae.Salinibacterium | 2.07920901384 | | | - | | Bacteria.Bacteroidetes.Bacteroidia.Bacteroidales.\_Odoribacteraceae\_ | 1.54019773569 | | | - | | Bacteria.Proteobacteria.Deltaproteobacteria.Bdellovibrionales.Bdellovibrionaceae | 2.19754361268 | | | - | | Bacteria.Actinobacteria.Actinobacteria.Actinomycetales.Intrasporangiaceae.Intrasporangium | 1.6136380462 | | | - | | Bacteria.Actinobacteria.Actinobacteria.Actinomycetales.Dietziaceae.Dietzia | 2.36120512487 | | | - | | Bacteria.Nitrospirae.Nitrospira.Nitrospirales.Nitrospiraceae | 1.8820247655 | | | - | | Bacteria.Proteobacteria.Gammaproteobacteria.Pasteurellales.Pasteurellaceae.Aggregatibacter | 1.12344372369 | | | - | | Bacteria.Proteobacteria.Alphaproteobacteria.Caulobacterales.Caulobacteraceae.Mycoplana | 1.6695458401 | | | - | | Bacteria.Firmicutes.Bacilli.Bacillales.Planococcaceae.Kurthia | 1.81727138188 | | | - | | Bacteria.Bacteroidetes.Cytophagia.Cytophagales | 3.06357892357 | | | - | | Bacteria.Chloroflexi.Thermomicrobia.JG30\_KF\_CM45 | 2.16270033213 | | | - | | Bacteria.Acidobacteria.Solibacteres.Solibacterales.AKIW659 | 1.4830493223 | | | - | | Bacteria.Tenericutes.Mollicutes.Mycoplasmatales.Mycoplasmataceae | 2.65679649881 | | | - | | Bacteria.Firmicutes.Clostridia.Clostridiales.Clostridiaceae | 4.11018136393 | | | - | | Bacteria.Bacteroidetes.\_Saprospirae\_.\_Saprospirales\_.Chitinophagaceae.Sediminibacterium | 4.40286049878 | | | - | | Bacteria.Proteobacteria.Deltaproteobacteria.NB1\_j | 2.35030102493 | | | - | | Bacteria.Chloroflexi.Anaerolineae.CFB\_26 | 1.71344128313 | | | - | | Bacteria.Firmicutes.Bacilli.Bacillales.Staphylococcaceae | 2.27292643624 | | | - | | Bacteria.Firmicutes.Clostridia.Thermoanaerobacterales.Thermoanaerobacteraceae | 1.69657050687 | | | - | | Bacteria.Proteobacteria.Betaproteobacteria.Rhodocyclales | 3.755557644 | | | - | | Bacteria.Actinobacteria.Actinobacteria.Actinomycetales.Mycobacteriaceae | 2.49219512185 | | | - | | Bacteria.Firmicutes.Bacilli.Bacillales.Staphylococcaceae.Salinicoccus | 2.04065635417 | | | - | | Bacteria.Actinobacteria.Actinobacteria.Actinomycetales.Pseudonocardiaceae.Amycolatopsis | 3.79110285578 | | | - | | Bacteria.Proteobacteria.Deltaproteobacteria.Desulfuromonadales.Geobacteraceae.Geobacter | 1.83706825997 | | | - | | Bacteria.Proteobacteria.Gammaproteobacteria.Enterobacteriales.Enterobacteriaceae.Proteus | 2.31024538318 | | | - | | Bacteria.Nitrospirae.Nitrospira.Nitrospirales.\_Thermodesulfovibrionaceae\_.LCP\_6 | 1.48668725381 | | | - | | Bacteria.Proteobacteria.Gammaproteobacteria | 4.91984018429 | | | - | | Bacteria.Chloroflexi.Ellin6529 | 1.90794766185 | | | - | | Bacteria.Bacteroidetes.Bacteroidia.Bacteroidales.Porphyromonadaceae.Parabacteroides | 1.98447929934 | | | - | | Bacteria.Actinobacteria.Actinobacteria.Actinomycetales.Cellulomonadaceae | 2.18170362409 | | | - | | Bacteria.Bacteroidetes.\_Saprospirae\_.\_Saprospirales\_.Chitinophagaceae | 4.40461810347 | | | - | | Bacteria.Firmicutes.Bacilli.Bacillales.\_Exiguobacteraceae\_ | 1.80000922607 | | | - | | Bacteria.Nitrospirae.Nitrospira.Nitrospirales.0319\_6A21 | 2.2970651381 | | | - | | Bacteria.Acidobacteria.Acidobacteria\_6.CCU21 | 1.96861141273 | | | - | | Bacteria.Verrucomicrobia.Verrucomicrobiae.Verrucomicrobiales.Verrucomicrobiaceae.Akkermansia | 3.24623103239 | | | - | | Bacteria.TM7.TM7\_1 | 1.56388435707 | | | - | | Bacteria.Chlorobi.SJA\_28 | 1.83652395809 | | | - | | Bacteria.Proteobacteria.Betaproteobacteria.Burkholderiales.Comamonadaceae.Alicycliphilus | 1.51047185653 | | | - | | Bacteria.Nitrospirae.Nitrospira.Nitrospirales.\_Thermodesulfovibrionaceae\_ | 1.98401189462 | | | - | | Bacteria.Spirochaetes.Spirochaetes.Sphaerochaetales.Sphaerochaetaceae.Sphaerochaeta | 2.06157237602 | | | - | | Bacteria.Cyanobacteria.Oscillatoriophycideae.Chroococcales.Gomphosphaeriaceae | 1.47673418135 | | | - | | Bacteria.Actinobacteria.Actinobacteria.Actinomycetales.Actinomycetaceae.Arcanobacterium | 3.69614034829 | | | - | | Bacteria.TM7 | 2.75282848673 | | | - | | Bacteria.Proteobacteria.Deltaproteobacteria.Desulfobacterales | 2.02704978446 | | | - | | Bacteria.Fusobacteria.Fusobacteriia | 5.23974633388 | | | - | | Bacteria.Proteobacteria.Betaproteobacteria.Burkholderiales.Oxalobacteraceae | 3.89318625305 | | | - | | Bacteria.Planctomycetes.Planctomycetia.Gemmatales | 2.22197325193 | | | - | | Bacteria.Actinobacteria.Actinobacteria.Actinomycetales.Nocardioidaceae.Kribbella | 1.4255816589 | | | - | | Bacteria.Bacteroidetes.\_Saprospirae\_.\_Saprospirales\_ | 4.40461810347 | | | - | | Bacteria.Fibrobacteres.Fibrobacteria.Fibrobacterales.Fibrobacteraceae | 1.47766715995 | | | - | | Bacteria.Proteobacteria.Gammaproteobacteria.Enterobacteriales.Enterobacteriaceae | 4.15948209572 | | | - | | Bacteria.Bacteroidetes.Bacteroidia.Bacteroidales.\_Barnesiellaceae\_.Barnesiella | 2.04507677807 | | | - | | Bacteria.Proteobacteria.Alphaproteobacteria.Rhizobiales.Xanthobacteraceae.Azorhizobium | 1.78609971859 | | | - | | Bacteria.Cyanobacteria.Oscillatoriophycideae.Chroococcales | 1.47673418135 | | | - | | Bacteria.Bacteroidetes.Bacteroidia.Bacteroidales.BS11 | 2.05037253378 | | | - | | Bacteria.Proteobacteria.Gammaproteobacteria.Thiotrichales | 2.22402537509 | | | - | | Bacteria.Firmicutes.Erysipelotrichi | 3.31548205546 | | | - | | Bacteria.Actinobacteria.Coriobacteriia.Coriobacteriales.Coriobacteriaceae | 3.67411950172 | | | - | | Bacteria.Actinobacteria.Coriobacteriia.Coriobacteriales.Coriobacteriaceae.Collinsella | 3.62957294246 | | | - | | Bacteria.Armatimonadetes.Chthonomonadetes.Chthonomonadales | 2.04143196548 | | | - | | Bacteria.Firmicutes.Bacilli.Turicibacterales.Turicibacteraceae.Turicibacter | 2.51314395699 | | | - | | Bacteria.Proteobacteria.Gammaproteobacteria.Vibrionales.Pseudoalteromonadaceae | 1.65282544041 | | | - | | Bacteria.Firmicutes.Bacilli.Turicibacterales | 2.51314395699 | | | - | | Bacteria.Armatimonadetes.Chthonomonadetes.Chthonomonadales.Chthonomonadaceae.Chthonomonas | 2.04143196548 | | | - | | Bacteria.Acidobacteria | 3.36385254642 | | | - | | Bacteria.Tenericutes.Mollicutes | 3.29359563197 | | | - | | Bacteria.Fibrobacteres | 1.69895406303 | | | - | | Bacteria.SR1 | 3.16902495763 | | | - | | Bacteria.Bacteroidetes.\_Saprospirae\_.\_Saprospirales\_.Chitinophagaceae.Flavisolibacter | 2.74449564832 | | | - | | Bacteria.Gemmatimonadetes.Gemmatimonadetes.Ellin5290 | 1.87884267402 | | | - | | Bacteria.Actinobacteria.Acidimicrobiia.Acidimicrobiales | 2.52173837048 | | | - | | Bacteria.Proteobacteria.Gammaproteobacteria.Alteromonadales.Alteromonadaceae | 1.33666663759 | | | - | | Bacteria.Actinobacteria.Actinobacteria.Actinomycetales.Micrococcaceae.Kocuria | 2.20727236553 | | | - | | Bacteria.Firmicutes.Clostridia.Clostridiales.Peptostreptococcaceae | 3.64886760542 | | | - | | Bacteria.Chloroflexi.Anaerolineae.Anaerolineales.Anaerolinaceae | 1.30064292229 | | | - | | Bacteria.Chloroflexi.Anaerolineae.DRC31 | 1.88157464632 | | | - | | Bacteria.Firmicutes.Clostridia.Clostridiales.Ruminococcaceae.Faecalibacterium | 3.25059586501 | | | - | | Bacteria.TM7.TM7\_3.CW040 | 2.55661008936 | | | - | | Bacteria.Actinobacteria.Actinobacteria.Actinomycetales.Corynebacteriaceae.Corynebacterium | 4.61869972568 | | | - | | Bacteria.Actinobacteria.Actinobacteria.Actinomycetales.Cellulomonadaceae.Actinotalea | 1.7972783019 | | | - | | Bacteria.Proteobacteria.Gammaproteobacteria.Enterobacteriales.Enterobacteriaceae.Erwinia | 1.48363463765 | | | - | | Bacteria.Proteobacteria.Betaproteobacteria.Burkholderiales.Oxalobacteraceae.Janthinobacterium | 1.76725761539 | | | - | | Bacteria.Nitrospirae.Nitrospira.Nitrospirales.\_Thermodesulfovibrionaceae\_.GOUTA19 | 1.81768047285 | | | - | | Bacteria.Chloroflexi.TK10.B07\_WMSP1 | 1.51103975057 | | | - | | Bacteria.Bacteroidetes.Bacteroidia.Bacteroidales | 4.80421783009 | | | - | | Bacteria.Proteobacteria.Deltaproteobacteria.Syntrophobacterales | 2.45480705076 | | | - | | Bacteria.Firmicutes.Clostridia.Clostridiales.\_Tissierellaceae\_.Helcococcus | 4.38282535163 | | | - | | Bacteria.Firmicutes.Bacilli.Lactobacillales.Leuconostocaceae | 1.59080365048 | | | - | | Bacteria.Planctomycetes.Planctomycetia | 2.22197325193 | | | - | | Bacteria.Proteobacteria.Alphaproteobacteria.Sphingomonadales.Sphingomonadaceae | 4.10009706085 | | | - | | Bacteria.Firmicutes.Bacilli.Bacillales.Thermoactinomycetaceae.Thermoactinomyces | 1.60199228281 | | | - | | Bacteria.Proteobacteria.Gammaproteobacteria.Pasteurellales | 3.54967735284 | | | - | | Bacteria.Proteobacteria.Gammaproteobacteria.Pasteurellales.Pasteurellaceae | 3.54967735284 | | | - | | Bacteria.Verrucomicrobia.Verruco\_5.WCHB1\_41 | 3.06316674641 | | | - | | Bacteria.Cyanobacteria.Chloroplast.Streptophyta | 2.97097150654 | | | - | | Bacteria.Planctomycetes.C6.MVS\_107 | 1.92570879462 | | | - | | Bacteria.Firmicutes.Clostridia.Clostridiales.Peptococcaceae | 3.48412623415 | | | - | | Bacteria.Actinobacteria.Rubrobacteria | 2.46569128599 | | | - | | Bacteria.Proteobacteria.Alphaproteobacteria.Caulobacterales | 3.92284851732 | | | - | | Bacteria.Proteobacteria.Alphaproteobacteria.Caulobacterales.Caulobacteraceae.Phenylobacterium | 1.28914029844 | | | - | | Bacteria.Actinobacteria.Actinobacteria.Actinomycetales.Micrococcaceae | 3.93079104358 | | | - | | Bacteria.Bacteroidetes.Bacteroidia.Bacteroidales.\_Paraprevotellaceae\_ | 2.41008108384 | | | - | | Bacteria.Bacteroidetes.Bacteroidia.Bacteroidales.Porphyromonadaceae.Paludibacter | 1.38376920718 | | | - | | Bacteria.Firmicutes.Erysipelotrichi.Erysipelotrichales.Erysipelotrichaceae.Bulleidia | 2.78202706415 | | | - | | Bacteria.Acidobacteria.AT\_s54 | 1.37624437582 | | | - | | Bacteria.Proteobacteria.Epsilonproteobacteria.Campylobacterales | 5.02508479325 | | | - | | Bacteria.Firmicutes.Bacilli.Lactobacillales.Streptococcaceae | 3.74731209149 | | | - | | Bacteria.Proteobacteria.Gammaproteobacteria.Oceanospirillales.Halomonadaceae.Halomonas | 1.43191566869 | | | - | | Bacteria.Proteobacteria.Deltaproteobacteria.Spirobacillales | 1.22034435638 | | | - | | Bacteria.Acidobacteria.Sva0725.Sva0725 | 1.44893864089 | | | - | | Bacteria.Firmicutes.Bacilli.Lactobacillales.Lactobacillaceae.Pediococcus | 1.86028817732 | | | - | | Bacteria.Lentisphaerae.\_Lentisphaeria\_.Z20 | 1.83301703377 | | | - | | Bacteria.Firmicutes.Bacilli | 5.34416838237 | | | - | | Bacteria.Actinobacteria.Actinobacteria.Actinomycetales.Nakamurellaceae | 1.37916388189 | | | - | | Bacteria.Firmicutes.Bacilli.Bacillales.Staphylococcaceae.Jeotgalicoccus | 2.19500269342 | | | - | | Bacteria.Acidobacteria.Solibacteres.Solibacterales | 2.88712709906 | | | - | | Bacteria.Proteobacteria.Deltaproteobacteria.Desulfuromonadales | 1.83706825997 | | | - | | Bacteria.Firmicutes.Bacilli.Lactobacillales.Aerococcaceae | 5.17116762657 | | | - | | Bacteria.Proteobacteria.Alphaproteobacteria.Rhizobiales.Rhizobiaceae.Rhizobium | 2.82451155241 | | | - | | Bacteria.Actinobacteria.Coriobacteriia | 3.67411950172 | | | - | | Bacteria.Firmicutes.Clostridia.Clostridiales.Veillonellaceae.Veillonella | 2.03407016934 | | | - | | Bacteria.Proteobacteria.Alphaproteobacteria.Sphingomonadales.Sphingomonadaceae.Novosphingobium | 2.55985368324 | | | - | | Bacteria.Bacteroidetes.Bacteroidia.Bacteroidales.Prevotellaceae | 2.47298427911 | | | - | | Bacteria.Tenericutes | 3.33265905688 | | | - | | Bacteria.Proteobacteria.Deltaproteobacteria.Myxococcales | 3.35931426948 | | | - | | Bacteria.Proteobacteria.Betaproteobacteria.Burkholderiales.Alcaligenaceae | 4.51770721329 | | | - | | Bacteria.Proteobacteria.Betaproteobacteria.Burkholderiales.Burkholderiaceae.Burkholderia | 1.98971945885 | | | - | | Bacteria.Proteobacteria.Betaproteobacteria.Burkholderiales.Comamonadaceae.Ramlibacter | 3.43974508617 | | | - | | Bacteria.Proteobacteria.Gammaproteobacteria.Cardiobacteriales.Cardiobacteriaceae | 2.6851933624 | | | - | | Bacteria.Actinobacteria.Thermoleophilia | 2.3713062986 | | | - | | Bacteria.Chloroflexi.Anaerolineae.H39 | 2.26151408852 | | | - | | Bacteria.Proteobacteria.Betaproteobacteria.Burkholderiales.Comamonadaceae.Comamonas | 2.81375618635 | | | - | | Bacteria.AD3.ABS\_6 | 2.14477171624 | | | - | | Bacteria.Firmicutes.Clostridia.Clostridiales.\_Tissierellaceae\_.GW\_34 | 4.78737208479 | | | - | | Bacteria.Proteobacteria.Gammaproteobacteria.Oceanospirillales | 1.43191566869 | | | - | | Bacteria.Firmicutes.Clostridia.Clostridiales | 5.32847092552 | | | - | | Bacteria.Verrucomicrobia.Verrucomicrobiae.Verrucomicrobiales.Verrucomicrobiaceae | 3.24623103239 | | | - | | Bacteria.Tenericutes.Mollicutes.Acholeplasmatales.Acholeplasmataceae | 3.10559509153 | | | - | | Bacteria.Firmicutes.Bacilli.Gemellales | 2.28204853748 | | | - | | Bacteria.\_Thermi\_.Deinococci.Deinococcales.Deinococcaceae.Deinococcus | 2.83790793463 | | | - | | Bacteria.Actinobacteria.Coriobacteriia.Coriobacteriales | 3.67411950172 | | | - | | Bacteria.Chloroflexi.Anaerolineae.SBR1031 | 2.80076113851 | | | - | | Bacteria.Fibrobacteres.Fibrobacteria.258ds10 | 1.30016991932 | | | - | | Bacteria.Firmicutes.Bacilli.Lactobacillales.Streptococcaceae.Lactococcus | 1.84402617635 | | | - | | Bacteria.Acidobacteria.\_Chloracidobacteria\_.RB41.Ellin6075 | 1.48166795428 | | | - | | Bacteria.Firmicutes.Clostridia.Clostridiales.Veillonellaceae.Megamonas | 1.60122150674 | | | - | | Bacteria.Gemmatimonadetes.Gemmatimonadetes.Gemmatimonadales | 2.49730541533 | | | - | | Bacteria.Planctomycetes.OM190.CL500\_15 | 1.35273442264 | | | - | | Bacteria.Acidobacteria.RB25 | 1.5653616235 | | | - | | Bacteria.Bacteroidetes.Flavobacteriia.Flavobacteriales.Flavobacteriaceae.Flavobacterium | 1.64221167663 | | | - | | Bacteria.Proteobacteria.Betaproteobacteria.Methylophilales.Methylophilaceae | 1.65932576829 | | | - | | Bacteria.Proteobacteria.Gammaproteobacteria.Pseudomonadales.Moraxellaceae.Moraxella | 3.92550169767 | | | - | | Bacteria.Firmicutes.Clostridia.Clostridiales.Peptostreptococcaceae.Peptostreptococcus | 2.97448326954 | | | - | | Bacteria.Proteobacteria.Betaproteobacteria.Rhodocyclales.Rhodocyclaceae.Dechloromonas | 1.9623326973 | | | - | | Bacteria.Actinobacteria.Actinobacteria.Actinomycetales.Nocardiaceae | 3.39623400578 | | | - | | Bacteria.Proteobacteria.Alphaproteobacteria.Rhizobiales.Methylocystaceae.Methylosinus | 1.36647239206 | | | - | | Bacteria.Firmicutes.Bacilli.Gemellales.Gemellaceae | 2.28204853748 | | | - | | Bacteria.Proteobacteria.Betaproteobacteria.Burkholderiales.Comamonadaceae.Rubrivivax | 1.57229636627 | | | - | | Bacteria.Bacteroidetes.Cytophagia | 3.06357892357 | | | - | | Bacteria.Actinobacteria.Actinobacteria.Actinomycetales.Streptomycetaceae.Streptomyces | 3.08685755408 | | | - | | Bacteria.Proteobacteria.Alphaproteobacteria.Caulobacterales.Caulobacteraceae.Asticcacaulis | 2.07998730326 | | | - | | Bacteria.Spirochaetes.Spirochaetes | 2.81568285925 | | | - | | Bacteria.Actinobacteria.Actinobacteria.Actinomycetales.Pseudonocardiaceae.Pseudonocardia | 1.65781900328 | | | - | | Bacteria.Proteobacteria.Gammaproteobacteria.Aeromonadales.Aeromonadaceae | 3.01049378551 | | | - | | Bacteria.Proteobacteria.Alphaproteobacteria.Caulobacterales.Caulobacteraceae.Brevundimonas | 2.76676293745 | | | - | | Bacteria.Bacteroidetes.Flavobacteriia.Flavobacteriales.Cryomorphaceae | 1.76552021223 | | | - | | Bacteria.Proteobacteria.Gammaproteobacteria.Xanthomonadales.Xanthomonadaceae.Stenotrophomonas | 1.35273442264 | | | - | | Bacteria.Elusimicrobia | 1.77497431319 | | | - | | Bacteria.Chloroflexi.Anaerolineae.SBR1031.SHA\_31 | 2.00037186658 | | | - | | Bacteria.Firmicutes.Erysipelotrichi.Erysipelotrichales.Erysipelotrichaceae.Allobaculum | 2.4218830311 | | | - | | Bacteria.Proteobacteria.Alphaproteobacteria.Rhodospirillales | 3.15225295461 | | | - | | Bacteria.Fusobacteria.Fusobacteriia.Fusobacteriales | 5.23974633388 | | | - | | Bacteria.Actinobacteria.Actinobacteria.Actinomycetales.Microbacteriaceae.Agrococcus | 1.24715282946 | | | - | | Bacteria.Bacteroidetes.Bacteroidia.Bacteroidales.Rikenellaceae.Blvii28 | 3.15861287281 | | | - | | Bacteria.Lentisphaerae | 3.15001334909 | | | - | | Bacteria.Firmicutes.Bacilli.Bacillales | 3.74121548383 | | | - | | Bacteria.Bacteroidetes.Flavobacteriia.Flavobacteriales | 3.16970947498 | | | - | | Bacteria.Actinobacteria.Actinobacteria.Actinomycetales.Propionibacteriaceae.Propionibacterium | 2.58747769837 | | | - | | Bacteria.Firmicutes.Clostridia.Clostridiales.\_Tissierellaceae\_.Parvimonas | 1.95242123758 | | | - | | Bacteria.Proteobacteria.Alphaproteobacteria.BD7\_3 | 1.89985748986 | | | - | | Bacteria.Lentisphaerae.\_Lentisphaeria\_ | 3.15001334909 | | | - | | Bacteria.Spirochaetes.Spirochaetes.Spirochaetales.Spirochaetaceae | 2.81568285925 | | | - | | Bacteria.OD1.ZB2 | 2.06616536604 | | | - | | Bacteria.Bacteroidetes.Bacteroidia.Bacteroidales.Bacteroidaceae.5\_7N15 | 3.26311910098 | | | - | | Bacteria.Bacteroidetes.Bacteroidia.Bacteroidales.\_Barnesiellaceae\_ | 2.63788262126 | | | - | | Bacteria.Firmicutes.Clostridia.Clostridiales.Veillonellaceae.Phascolarctobacterium | 2.47470299063 | | | - | | Bacteria.Proteobacteria.Betaproteobacteria.Burkholderiales.Alcaligenaceae.Achromobacter | 4.51421903366 | | | - | | Bacteria.Firmicutes.Bacilli.Lactobacillales.Aerococcaceae.Aerococcus | 3.6515703656 | | | - | | Bacteria.Firmicutes.Clostridia.Clostridiales.Lachnospiraceae.Oribacterium | 1.88071317329 | | | - | | Bacteria.Actinobacteria.Coriobacteriia.Coriobacteriales.Coriobacteriaceae.Eggerthella | 1.63148802359 | | | - | | Bacteria.Proteobacteria.Deltaproteobacteria.Desulfovibrionales.Desulfovibrionaceae.Bilophila | 2.74540168377 | | | - | | Bacteria.Proteobacteria.Betaproteobacteria.Rhodocyclales.Rhodocyclaceae | 3.755557644 | | | - | | Bacteria.Firmicutes.Erysipelotrichi.Erysipelotrichales.Erysipelotrichaceae.Catenibacterium | 2.02046994967 | | | - | | Bacteria.Proteobacteria.Betaproteobacteria.Burkholderiales | 4.75646044707 | | | - | | Bacteria.Proteobacteria.Betaproteobacteria.Burkholderiales.Oxalobacteraceae.Cupriavidus | 3.74564962767 | | | - | | Bacteria.Actinobacteria.Thermoleophilia.Gaiellales | 2.17102200139 | | | - | | Bacteria.Proteobacteria.Betaproteobacteria.Rhodocyclales.Rhodocyclaceae.Azospira | 3.44086397011 | | | - | | Bacteria.Proteobacteria.Alphaproteobacteria.Rickettsiales | 2.23712693218 | | | - | | Bacteria.Actinobacteria.Actinobacteria.Actinomycetales.Nocardioidaceae.Nocardioides | 1.38195190329 | | | - | | Bacteria.Proteobacteria.Alphaproteobacteria.Rhizobiales.Hyphomicrobiaceae.Pedomicrobium | 1.37011194114 | | | - | | Bacteria.Proteobacteria.Alphaproteobacteria.Rhizobiales.Phyllobacteriaceae.Aminobacter | 3.33744083961 | | | - | | Bacteria.Cyanobacteria.ML635J\_21 | 1.52387352437 | | | - | | Bacteria.Spirochaetes.Spirochaetes.Sphaerochaetales.Sphaerochaetaceae | 2.06157237602 | | | - | | Bacteria.Proteobacteria.Betaproteobacteria.Hydrogenophilales | 2.23178460753 | | | - | | Bacteria.Proteobacteria.Deltaproteobacteria.MBNT15 | 2.09445499623 | | | - | | Bacteria.Proteobacteria.Alphaproteobacteria.Rhodobacterales.Rhodobacteraceae.Rhodobacter | 2.11317806288 | | | - | | Bacteria.Proteobacteria.Alphaproteobacteria.Rhizobiales.Bradyrhizobiaceae | 3.21386825184 | | | - | | Bacteria.Actinobacteria.Actinobacteria.Actinomycetales.Propionibacteriaceae | 2.83843912095 | | | - | | Bacteria.Chloroflexi.TK10 | 1.51103975057 | | | - | | Bacteria.Chloroflexi.TK17 | 1.89479788061 | | | - | | Bacteria.Proteobacteria.Gammaproteobacteria.Alteromonadales.Alteromonadaceae.Marinobacter | 1.33666663759 | | | - | | Bacteria.Chlorobi | 1.83652395809 | | | - | | Bacteria.Proteobacteria.Gammaproteobacteria.Thiotrichales.Thiotrichaceae.Thiothrix | 1.3975529353 | | | - | | Bacteria.Planctomycetes.Phycisphaerae.Phycisphaerales.Phycisphaeraceae | 1.70609257466 | | | - | | Bacteria.Bacteroidetes.Bacteroidia | 4.80421783009 | | | - | | Bacteria.Firmicutes.Bacilli.Bacillales.Thermoactinomycetaceae | 1.60199228281 | | | - | | Bacteria.Chloroflexi.Chloroflexi.AKIW781 | 1.45191059763 | | | - | | Bacteria.Bacteroidetes.Bacteroidia.Bacteroidales.Prevotellaceae.Prevotella | 2.47298427911 | | | - | | Bacteria.Firmicutes.Bacilli.Gemellales.Gemellaceae.Gemella | 2.27052758534 | | | - | | Bacteria.Cyanobacteria.Chloroplast.Stramenopiles | 1.72736763249 | | | - | | Bacteria.Firmicutes.Clostridia.Clostridiales.Eubacteriaceae.Anaerofustis | 1.63917543713 | | | - | | Bacteria.Proteobacteria.Gammaproteobacteria.Cardiobacteriales.Cardiobacteriaceae.Suttonella | 2.6851933624 | | | - | | Bacteria.Bacteroidetes.Bacteroidia.Bacteroidales.Marinilabiaceae | 1.30190453133 | | | - | | Bacteria.Proteobacteria.Alphaproteobacteria.Rhizobiales.Hyphomicrobiaceae | 2.81303332046 | | | - | | Bacteria.Verrucomicrobia.Verruco\_5.WCHB1\_41.RFP12 | 3.0100804693 | | | - | | Bacteria.Tenericutes.CK\_1C4\_19 | 1.99986427093 | | | - | | Bacteria.Tenericutes.RF3 | 2.22892822429 | | | - | | Bacteria.Acidobacteria.Acidobacteriia.Acidobacteriales.Koribacteraceae.Candidatus\_Koribacter | 1.39641686149 | | | - | | Bacteria.Firmicutes.Erysipelotrichi.Erysipelotrichales.Erysipelotrichaceae.p\_75\_a5 | 2.47418564167 | | | - | | Bacteria.Cyanobacteria.4C0d\_2.MLE1\_12 | 3.79548019624 | | | - | | Bacteria.Firmicutes.Clostridia.Clostridiales.Ruminococcaceae.Ruminococcus | 3.04787004037 | | | - | | Bacteria.Firmicutes.Bacilli.Lactobacillales | 5.34045490403 | | | - | | Bacteria.Tenericutes.Mollicutes.Acholeplasmatales.Acholeplasmataceae.Acholeplasma | 3.09967412269 | | | - | | Bacteria.Actinobacteria.Actinobacteria.Actinomycetales.Geodermatophilaceae | 2.27669512021 | | | - | | Bacteria.Bacteroidetes.Flavobacteriia.Flavobacteriales.\_Weeksellaceae\_.Cloacibacterium | 2.7071560275 | | | - | | Bacteria.Firmicutes.Bacilli.Lactobacillales.Aerococcaceae.Alloiococcus | 3.54086426726 | | | - | | Bacteria.Armatimonadetes.\_Fimbriimonadia\_.\_Fimbriimonadales\_.\_Fimbriimonadaceae\_.Fimbriimonas | 2.06862190152 | | | - | | Bacteria.Proteobacteria.Betaproteobacteria.Rhodocyclales.Rhodocyclaceae.Zoogloea | 3.37730094843 | | | - | | Bacteria.Acidobacteria.DA052.Ellin6513 | 1.7816287422 | | | - | | Bacteria.Proteobacteria.Alphaproteobacteria.Sphingomonadales.Erythrobacteraceae | 2.28913979262 | | | - | | Bacteria.Proteobacteria.Gammaproteobacteria.Cardiobacteriales | 2.6851933624 | | | - | | Bacteria.Firmicutes.Bacilli.Lactobacillales.Carnobacteriaceae.Desemzia | 1.83896850562 | | | - | | Bacteria.Chloroflexi.Anaerolineae.Anaerolineales | 1.30064292229 | | | - | | Bacteria.Actinobacteria.Actinobacteria.Actinomycetales.Brevibacteriaceae | 2.78590003124 | | | - | | Bacteria.Firmicutes.Clostridia.Clostridiales.\_Acidaminobacteraceae\_ | 4.1215622029 | | | - | | Bacteria.Proteobacteria.Deltaproteobacteria.Bdellovibrionales | 2.19754361268 | | | - | | Bacteria.Proteobacteria.Deltaproteobacteria.Desulfobacterales.Desulfobacteraceae.Desulfococcus | 1.75247994994 | | | - | | Bacteria.Firmicutes.Bacilli.Lactobacillales.Lactobacillaceae | 3.66651238933 | | | - | | Bacteria.Proteobacteria.Deltaproteobacteria.Desulfovibrionales.Desulfovibrionaceae | 3.49864984251 | | | - | | Bacteria.Chloroflexi | 3.24276345877 | | | - | | Bacteria.Fibrobacteres.Fibrobacteria.Fibrobacterales.Fibrobacteraceae.Fibrobacter | 1.47766715995 | | | - | | Bacteria.Firmicutes.Clostridia.Clostridiales.Veillonellaceae | 3.42506936224 | | | - | | Bacteria.Actinobacteria.Actinobacteria.Actinomycetales.Dermabacteraceae.Dermabacter | 1.52882568169 | | | - | | Bacteria.Proteobacteria.Gammaproteobacteria.HOC36 | 1.51073544665 | | | - | | Bacteria.Proteobacteria.Betaproteobacteria.Burkholderiales.Oxalobacteraceae.Herbaspirillum | 3.29145736625 | | | - | | Bacteria.Firmicutes.Clostridia.Clostridiales.\_Tissierellaceae\_ | 5.14233304403 | | | - | | Bacteria.Bacteroidetes.Bacteroidia.Bacteroidales.Porphyromonadaceae.Porphyromonas | 4.70335956752 | | | - | | Bacteria.Verrucomicrobia.Verrucomicrobiae | 3.24623103239 | | | - | | Bacteria.Firmicutes.Clostridia.Clostridiales.\_Tissierellaceae\_.Peptoniphilus | 4.3276739457 | | | - | | Bacteria.Firmicutes.Clostridia | 5.32857227773 | | | - | | Bacteria.Actinobacteria.Actinobacteria.Actinomycetales.Cellulomonadaceae.Cellulomonas | 1.95060563563 | | | - | | Bacteria.Firmicutes.Clostridia.Clostridiales.Lachnospiraceae.Coprococcus | 3.38519747323 | | | - | | Bacteria.Proteobacteria.Deltaproteobacteria.Myxococcales.0319\_6G20 | 1.73726412524 | | | - | | Bacteria.Actinobacteria.Actinobacteria.Actinomycetales.Yaniellaceae | 1.48471264083 | | | - | | Bacteria.Proteobacteria.Alphaproteobacteria.Rhizobiales.Rhizobiaceae.Agrobacterium | 3.36914041418 | | | - | | Bacteria.Firmicutes.Bacilli.Bacillales.Planococcaceae.Sporosarcina | 1.52276297395 | | | - | | Bacteria.Tenericutes.Mollicutes.Mycoplasmatales | 2.65679649881 | | | - | | Bacteria.Firmicutes.Clostridia.Clostridiales.Clostridiaceae.Clostridium | 4.09411644882 | | | - | | Bacteria.Bacteroidetes.Cytophagia.Cytophagales.Cyclobacteriaceae | 1.79404568761 | | | - | | Bacteria.Actinobacteria.Actinobacteria.Actinomycetales.Dermabacteraceae.Brachybacterium | 1.91251075718 | | | - | | Bacteria.GN02 | 3.12288815943 | | | - | | Bacteria.Planctomycetes | 3.04542593522 | | | - | | Bacteria.Fibrobacteres.Fibrobacteria | 1.69895406303 | | | - | | Bacteria.WS3.PRR\_12.Sediment\_1 | 2.2191228881 | | | - | | Bacteria.Acidobacteria.\_Chloracidobacteria\_.DS\_100 | 1.53904946142 | | | - | | Bacteria.Proteobacteria.Alphaproteobacteria.Rickettsiales.mitochondria.Zea | 1.94715947205 | | | - | | Bacteria.Proteobacteria.Gammaproteobacteria.Alteromonadales.Shewanellaceae.Shewanella | 1.84525210506 | | | - | | Bacteria.Proteobacteria.Epsilonproteobacteria.Campylobacterales.Campylobacteraceae.Campylobacter | 5.0243760106 | | | - | | Bacteria.Proteobacteria.Deltaproteobacteria.Desulfuromonadales.Geobacteraceae | 1.83706825997 | | | - | | Bacteria.Firmicutes.Bacilli.Lactobacillales.Enterococcaceae.Enterococcus | 1.61789116692 | | | - | | Bacteria.\_Thermi\_.Deinococci.Deinococcales | 2.83790793463 | | | - | | Bacteria.Bacteroidetes.Bacteroidia.Bacteroidales.Rikenellaceae.Alistipes | 1.66349745394 | | | - | | Bacteria.Elusimicrobia.Elusimicrobia.Elusimicrobiales | 1.77497431319 | | | - | | Bacteria.Actinobacteria.Actinobacteria.Actinomycetales.Nocardioidaceae | 2.92058264928 | | | - | | Bacteria.Tenericutes.Mollicutes.Mycoplasmatales.Mycoplasmataceae.Mycoplasma | 2.65679649881 | | | - | | Bacteria.Proteobacteria.Deltaproteobacteria.Bdellovibrionales.Bdellovibrionaceae.Bdellovibrio | 2.19754361268 | | | - | | Bacteria.\_Thermi\_.Deinococci.Thermales.Thermaceae | 1.93681648739 | | | - | | Bacteria.Proteobacteria.Betaproteobacteria.Rhodocyclales.Rhodocyclaceae.Petrobacter | 2.25911368859 | | | - | | Bacteria.Proteobacteria.Betaproteobacteria.SC\_I\_84 | 2.73168825806 | | | - | | Bacteria.Firmicutes.Clostridia.Clostridiales.Peptostreptococcaceae.Filifactor | 2.83239776988 | | | - | | Bacteria.Proteobacteria.Deltaproteobacteria.GMD14H09 | 2.24505463924 | | | - | | Bacteria.Actinobacteria.Acidimicrobiia.Acidimicrobiales.EB1017 | 1.83993683493 | | | - | | Bacteria.Bacteroidetes.Sphingobacteriia | 2.43278962547 | | | - | | Bacteria.Proteobacteria.Gammaproteobacteria.Thiotrichales.Thiotrichaceae | 1.3975529353 | | | - | | Bacteria.Planctomycetes.Planctomycetia.Gemmatales.Isosphaeraceae | 1.9117341765 | | | - | | Bacteria.Actinobacteria.Actinobacteria.Actinomycetales.Actinomycetaceae.Mobiluncus | 3.77189196272 | | | - | | Bacteria.Proteobacteria.Gammaproteobacteria.Thiotrichales.Piscirickettsiaceae | 2.20000907143 | | | - | | Bacteria.Verrucomicrobia.Verrucomicrobiae.Verrucomicrobiales | 3.24623103239 | | | - | | Bacteria.Chloroflexi.Anaerolineae.GCA004 | 2.06990036179 | | | - | | Bacteria.Firmicutes.Clostridia.Clostridiales.\_Tissierellaceae\_.Tepidimicrobium | 2.12283643698 | | | - | | Bacteria.Deferribacteres.Deferribacteres.Deferribacterales.Deferribacteraceae.Mucispirillum | 1.76534317471 | | | - | | Bacteria.Proteobacteria.Alphaproteobacteria.Rickettsiales.mitochondria | 1.94715947205 | | | - | | Bacteria.Firmicutes.Bacilli.Lactobacillales.Carnobacteriaceae | 4.84162192373 | | | - | | Bacteria.Bacteroidetes.Sphingobacteriia.Sphingobacteriales | 2.43278962547 | | | - | | Bacteria.Actinobacteria | 4.88253069986 | | | - | | Bacteria.Proteobacteria.Gammaproteobacteria.Pseudomonadales.Moraxellaceae.Enhydrobacter | 3.11662300033 | | | - | | Bacteria.Proteobacteria.Gammaproteobacteria.\_Marinicellales\_ | 1.63853659763 | | | - | | Bacteria.Proteobacteria.Betaproteobacteria.Burkholderiales.Alcaligenaceae.Sutterella | 2.20945125729 | | | - | | Bacteria.Proteobacteria.Alphaproteobacteria.Sphingomonadales.Sphingomonadaceae.Sphingobium | 2.78611672459 | | | - | | Bacteria.Armatimonadetes.\_Fimbriimonadia\_.\_Fimbriimonadales\_.\_Fimbriimonadaceae\_ | 2.06862190152 | | | - | | Bacteria.WS3.PRR\_12 | 2.2191228881 | | | - | | Bacteria.Actinobacteria.Actinobacteria.Bifidobacteriales.Bifidobacteriaceae | 3.80908859425 | | | - | | Bacteria.Gemmatimonadetes.Gemmatimonadetes | 3.00310773923 | | | - | | Bacteria.Chloroflexi.Ktedonobacteria | 1.85815132074 | | | - | | Bacteria.Deferribacteres.Deferribacteres.Deferribacterales | 1.76534317471 | | | - | | Bacteria.Chloroflexi.Gitt\_GS\_136 | 1.54677152286 | | | - | | Bacteria.Actinobacteria.Actinobacteria.Actinomycetales.Streptomycetaceae | 3.08685755408 | | | - | | Bacteria.Fusobacteria.Fusobacteriia.Fusobacteriales.Fusobacteriaceae.Fusobacterium | 4.83365775878 | | | - | | Bacteria.Acidobacteria.Acidobacteriia | 2.35836521613 | | | - | | Bacteria.Proteobacteria.Gammaproteobacteria.Aeromonadales | 3.01049378551 | | | - | | Bacteria.Firmicutes.Bacilli.Bacillales.Planococcaceae.Planomicrobium | 2.10595164953 | | | - | | Bacteria.Proteobacteria.Deltaproteobacteria.Syntrophobacterales.Syntrophobacteraceae | 2.45480705076 | | | - | | Bacteria.Planctomycetes.Planctomycetia.Gemmatales.Gemmataceae | 1.92996110826 | | | - | | Bacteria.Spirochaetes.Spirochaetes.Sphaerochaetales | 2.06157237602 | | | - | | Bacteria.Proteobacteria.Deltaproteobacteria.Desulfobacterales.Desulfobacteraceae | 1.75247994994 | | | - | | Bacteria.Bacteroidetes.Bacteroidia.Bacteroidales.Porphyromonadaceae | 4.70439605969 | | | - | | Bacteria.Proteobacteria.Gammaproteobacteria.Alteromonadales.Shewanellaceae | 1.84525210506 | | | - | | Bacteria.Actinobacteria.Actinobacteria.Actinomycetales.Mycobacteriaceae.Mycobacterium | 2.49219512185 | | | - | | Bacteria.Firmicutes.Clostridia.Clostridiales.\_Tissierellaceae\_.Finegoldia | 2.4703459292 | | | - | | Bacteria.Actinobacteria.Actinobacteria.Actinomycetales.Yaniellaceae.Yaniella | 1.48471264083 | | | - | | Bacteria.Chloroflexi.Anaerolineae | 3.06318864124 | | | - | | Bacteria.Firmicutes.Bacilli.Bacillales.\_Thermicanaceae\_ | 1.51073544665 | | | - | 注：第一列为样本群落中从门到属的所有分类单元列表，LEfSe会逐一判断这些分类单元的组间差异是否具有统计学显著性。第二列的数值表明各分类单元所具有的最高组内相对丰度均值（为对数转换值）。如果该分类单元未体现出显著的组间差异，则后三列为空；而对于具有统计学差异的分类单元，LEfSe将在第三列中列出该分类单元对应的平均丰度最高的分组，并给出LDA差异分析的对数得分值（第四列，默认要求> 2），以及P值大小（第五列，默认< 0.05）。通常可根据第四列的LDA差异分析对数得分值和第五列的P值，描述在组间具有显著差异的分类单元的统计学效力强弱。 **组间具有显著差异的分类单元** 注：纵坐标为组间具有显著差异的分类单元，横坐标则以条形图直观地展示对应分类单元的LDA差异分析对数得分值，并按照得分值大小进行排序，以此描述它们在不同分组样本中的差异大小。长度越长表明该分类单元的差异越显著，条形图的不同颜色指示了该分类单元所对应的丰度较高的样本分组。 **基于分类等级树的组间差异分类单元展示图** 注：分类等级树展示了样本群落中从门到属（从内圈到外圈依次排列）所有分类单元的等级关系，节点大小对应于该分类单元的平均相对丰度，黄色节点代表未体现出显著的组间差异的分类单元，而其它色（如绿色和红色）则表明这些分类单元体现出显著的组间差异，且在该色所代表分组样本中丰度较高。字母则标识了组间存在显著差异的分类单元名称。 **具有显著差异的分类单元在不同分组中的相对丰度分布** 注：对于组间具有显著差异的分类单元，LEfSe还能展示其在不同样本分组（以“class”标识）中的相对丰度分布，并分别以实线和虚线标识该分类单元在每一分组中的相对丰度平均值和中位值，从而直观地体现组间差异大小。 结果目录: C01\_diff/lefse 系统发育树和分类等级树的构建 在2.1节划分OTU的过程中，通过FastTree工具(Price et al., 2009)构建了OTU代表序列的系统发育树，该文件（rep\_set.tre）以Newick格式保存（https://en.wikipedia.org/wiki/Newick\_format），可以用MEGA（http://www.megasoftware.net/）、FigTree（http://tree.bio.ed.ac.uk/software/figtree/）或TopiaryExplorer（http://topiaryexplorer.sourceforge.net/）等软件打开、编辑。 (SEQ1:0.02120,SEQ2:0.09111,(SEQ3:0.04491,SEQ4:0.00097)SEQ5:0.00194)SEQ6; Newick序列树文件格式示例 注：处在同一层括号内的为聚类在一个节点的序列分枝，冒号后的数字表示分枝长度。 除此之外，还可以利用各样本的分类学组成，通过多种软件构建分类等级树 基于MEGAN的分类学组成信息可视化 方法步骤 使用软件MEGAN（http://ab.inf.uni-tuebingen.de/software/megan6/）(Huson et al., 2011)，将每个样本所含有的OTU的丰度信息和分类学组成数据映射至NCBI Taxonomy所提供的微生物分类等级树（https://www.ncbi.nlm.nih.gov/taxonomy），从而可以在一套标准的分类等级系统中，统一呈现所有样本在各分类水平的具体组成。 结果说明 **物种进化及丰度信息图** 注：分类等级树的每个分枝节点的饼图表明该分类单元在各样本中的丰度高低，扇形面积越大，表明该分类单元在对应样本中的丰度越高。 结果目录: B08\_megan 基于GraPhlAn的分类学组成信息可视化 方法步骤 使用GraPhlAn这一最近涌现的可视化工具(Asnicar et al., 2015)，对样本总体在各分类水平的组成构建等级树，同时以不同颜色区分各分类单元，并通过节点大小反映它们的丰度分布。与MEGAN相比，GraPhlAn绘制的分类等级树提供了一种从复杂的群落数据中，快速发现优势微生物类群的方法。 结果说明 **基于GraPhlAn的样本总体分类等级树图** 注：分类等级树展示了样本总体中，从门到属（从内圈到外圈依次排列）所有分类单元（以节点表示）的等级关系，节点大小对应于该分类单元的平均相对丰度，相对丰度前20位的分类单元还将在图中以字母标识（从门到属按照从外层到内层依次排列），字母上的阴影颜色同对应节点颜色一致。 结果目录: B09\_graphlan 基于Krona的分类学组成信息交互展示 方法步骤 使用Krona软件（https://github.com/marbl/Krona/wiki）进行群落分类学组成的交互展示(Ondov et al., 2011)。相比于上述的MEGAN和GraPhlAn两种软件，Krona不仅可以对样本分类学组成进行可视化分析，更侧重于数据的交互展示。 结果说明 A01.html A02.html A03.html A04.html A05.html A06.html A07.html A08.html A09.html A10.html A11.html A12.html A13.html B01.html B02.html B03.html B04.html B05.html B06.html B07.html B08.html B09.html B10.html **基于Krona的分类学组成信息交互展示图** 注：该网页文件对样本分类学组成进行交互展示，圆圈从内到外依次代表门、纲、目、科、属五个分类水平，扇形的大小反映了不同分类单元的相对丰度高低，并给出具体数值。在每个分类水平，各单元以不同的颜色加以区分。网页左上方提供了图形的可视化选项，可通过点击对应按钮调整图形的尺寸和显示的分类层次的深度等。点击图形中某一分类单元，将在网页右上方显示它在各分类层次中所占的比例。 结果目录: B10\_krona 热图分析 OTU热图交互式网页 方法步骤 为了快速、直观、灵活地显示群落样本的OTU组成及丰度信息，使用QIIME软件构建了以下OTU热图的交互式网页，可将网页格式的文件通过浏览器打开查看结果。 结果说明 **通过交互式网页查看OTU热图** 注：每一色块代表一个OTU在对应样本中的丰度，色块上的数字表明该OTU在对应样本中的实际序列数，颜色反映了该OTU在对应样本中丰度的高低（蓝色表示低丰度，红色表示高丰度）。 结合聚类分析的群落组成热图 将各分类水平的群落组成数据根据分类单元的丰度分布或样本间的相似程度加以聚类，根据聚类结果对分类单元和样本分别排序，并通过热图加以呈现。通过聚类，可以将高丰度和低丰度的分类单元加以区分，并以颜色梯度反映样本之间的群落组成相似度。 方法步骤 使用R软件，对丰度前50位的属进行聚类分析并绘制热图。 结果说明 **结合聚类分析的属水平群落组成热图** 注：样本先按照彼此之间组成的相似度进行聚类，根据聚类结果横向依次排列。同理，分类单元也按照彼此在不同样本中分布的相似度进行聚类，根据聚类结果纵向依次排列。图中，红色代表在对应样本中丰度较高的属，绿色代表丰度较低的属。 结果目录: B11\_heatmap Beta多样性分析 Beta多样性分析的主要目的是考察不同样本之间群落结构的相似性。主要通过主成分分析（Principal component analysis，PCA）、多维尺度分析（Multidimensional scaling，MDS）和聚类分析（Clustering analysis）三类方法，对群落数据结构进行自然分解并通过对样本排序（Ordination），从而观测样本之间的差异。 PCA主成分分析 PCA分析通过线性变换，将原始的高维数据（如菌群OTU丰度矩阵）通过线性变换组合，投影到维度较低的空间坐标系（即主成分）中，从而达到降维、简化数据结构的目的，展现样本的自然分布(Ramette, 2007)。PCA分析能够从原始数据中提取样本间最主要的差异特征，并根据这些差异特征将样本在新的低维坐标系中依次排序，使得样本在新坐标系中的距离远近能在最大程度上还原样本间的实际差异。在这排序过程中，每一坐标轴对原始数据中样本差异的解释比例依次下降。因此，通过对PCA分析得到的前二维或三维数据作图，可以得知群落样本的主要分布特征，从而量化样本间的差异和相似度。PCA分析基于欧式距离评价样本之间的相似度，不考虑原始变量之间可能存在的相互关系。 方法步骤 通过R软件，对属水平的群落组成结构进行PCA分析，并且以二维和三维图像描述样本间的自然分布特征。 结果说明 **PCA分析的样本二维排序图** 注：每个点代表一个样本，不同颜色的点属于不同样本（组），两点之间的距离越近，表明两个样本之间的微生物群落结构相似度越高，差异越小。坐标轴括号中的百分比代表了对应的主成分所能解释的原始数据中差异的比例。 **PCA分析的样本三维排序图** 注：每个点代表一个样本，不同颜色的点属于不同样本（组），两点之间的距离越近，表明两个样本之间的微生物群落结构相似度越高，差异越小。坐标轴括号中的百分比代表了对应的主成分所能解释的原始数据中差异的比例。 结果目录: B12\_pca 基于UniFrac距离的PCoA主坐标分析 由于微生物极其多样，不同微生物彼此之间具有特定的系统发育关系，意味着群落中的各微生物成员（如OTU）之间存在某种内在关联。因此，在比较不同群落样本之间的差异时，需要考虑两个群落成员之间是否存在系统发育亲缘关系。UniFrac距离就是基于这个思路，通过比较两个群落各自独有OTU之间系统发育关系的远近，从而更全面地反映群落样本之间的相似程度(Lozupone and Knight, 2005)。 MDS分析与PCA分析类似，但是它可以基于任意距离尺度（如UniFrac距离）评价样本之间的相似度。主坐标分析（Principal coordinates analysis，PCoA）是其中一种经典的MDS分析方法(Ramette, 2007)，通过对样本距离矩阵作降维分解，从而简化数据结构，展现样本在某种特定距离尺度下的自然分布(Ramette, 2007)。 方法步骤 首先使用QIIME软件，对UniFrac PCoA分析得到的前二维或三维数据作图，从而得知基于微生物系统发育关系的群落样本空间分布特征，量化样本间的差异和相似度。UniFrac距离有Unweighted和Weighted之分，前者仅仅考虑OTU在样本中存在与否，而不考虑其丰度高低(Lozupone and Knight, 2005)；后者则兼顾群落成员之间的系统发育关系以及它们在各自样本中的丰度高低(Lozupone et al., 2007)。因此，Unweighted UniFrac距离侧重于描述由群落成员的截然不同导致的样本差异，Weighted UniFrac距离则侧重于描述由群落成员丰度梯度的改变导致的样本差异。 结果说明 **Unweighted UniFrac PCoA分析的样本三维排序图** **Weighted UniFrac PCoA分析的样本三维排序图** 注：每个点代表一个样本，不同颜色的点属于不同样本（组），两点之间的距离越近，表明两个样本之间的微生物群落结构相似度越高，差异越小。坐标轴括号中的百分比代表了对应的主坐标所能解释的原始数据中差异的比例。 Unweighted UniFrac PCoA结果点击此处 Weighted UniFrac PCoA结果点击此处 基于UniFrac距离的NMDS非度量多维尺度分析 NMDS分析（Nonmetric Multidimensional Scaling）与上述PcoA分析类似，也是一种基于样本距离矩阵的MDS分析方法，通过降维处理简化数据结构，在新的低维坐标系中对样本重新排序，从而在特定距离尺度下描述样本的分布特征。与PcoA分析不同，NMDS分析不依赖于特征根和特征向量的计算，而是通过对样本距离进行等级排序，使样本在低维空间中的排序尽可能符合彼此之间的距离远近关系（而非确切的距离数值）。因此，NMDS分析不受样本距离的数值影响，仅考虑彼此之间的大小关系，对于结构复杂的数据，排序结果可能更稳定。 方法步骤 使用R软件对Unweighted和Weighted的UniFrac距离矩阵分别进行NMDS分析，通过二维或三维排序图描述群落样本的结构分布。 结果说明 **Unweighted UniFrac NMDS分析的样本二维排序图** **Weighted UniFrac NMDS分析的样本二维排序图** 注：每个点代表一个样本，不同颜色的点属于不同样本（组），两点之间的距离越近，表明两个样本之间的微生物群落结构相似度越高，差异越小。 基于UniFrac距离的样本聚类分析 聚类分析主要指层次聚类（Hierarchical clustering）的分析方法，以等级树的形式展示样本间的相似度，通过聚类树的分枝长度衡量聚类效果的好坏。与MDS分析相同，聚类分析可以采用任何距离评价样本之间的相似度。常用的聚类分析方法包括非加权组平均法（Unweighted pair-group method with arithmetic means，UPGMA）、单一连接法（Single-linkage clustering）和完全连接法（Complete-linkage clustering）等。 方法步骤 使用QIIME软件，对Unweighted和Weighted的UniFrac距离矩阵分别进行UPGMA聚类分析，并使用R软件进行可视化。 结果说明 **基于Unweighted UniFrac距离矩阵的UPGMA聚类分析图** **基于Weighted UniFrac距离矩阵的UPGMA聚类分析图** 注：样本根据彼此之间的相似度聚类，样本间的分枝长度越短，两样本越相似。 结果目录: B13\_bdiv UniFrac距离组间/组内差异比较分析 根据样本距离矩阵，可以对不同分组的样本距离均值进行统计学比较检验，通过对不同的样本分组两两进行t检验，并通过蒙特卡罗置换检验（Monte Carlo permutation test，将原始的样本分组信息多次随机打乱，如果原始样本分组有规律可循，那么它有较大的可能性与随机置换的样本分组具有显著差异）获得统计检验的显著性P值，从而衡量组内和组间距离差异，得出具有生物学意义的结论。一般而言，同组样本彼此之间的距离大小反映了组内的个体间差异，如果组间差异显著高于组内差异，表明两组样本存在统计学差异。相比上述MDS降维排序分析，该分析的优点是直接使用距离矩阵进行统计检验，因此最大程度上保留了原始数据的完整性。 方法步骤 使用QIIME软件，根据样本分组，分别对Unweighted和Weighted的UniFrac距离矩阵的组内和组间距离均值进行t检验，并通过1000次蒙特卡罗置换检验判断统计显著性。 结果说明 不同分组的UniFrac距离值差异可以通过箱线图进行展示，根据中位值、上下四分位值和最大最小值，可以呈现每组数据的具体分布特征，识别数据异常值（Outlier）。结合统计检验结果，有助于更全面地描述组内和组间的菌群结构差异大小。 **Unweighted UniFrac距离的多组比较箱线图** **Weighted UniFrac距离的多组比较箱线图** 注：横坐标对应各组组间和组内的统计比较，纵坐标表明相应的距离值，箱形图边框代表上下四分位数间距（Interquartile range，IQR），横线代表中位值，上下触须分别代表上下四分位以外的1.5倍IQR范围，符号“+”表示超过范围的潜在异常值。 结果目录: B13\_bdiv\boxplot 菌群比较分析和关键物种筛选 解析高通量测序产生的海量的微生物群落数据，还需要借助约束排序（Constrained ordination）和有监督学习（Supervised learning）的统计分析方法，通过某种已知的样本间相互关系（比如某种样本分布/分组）或某种样本检测指标（连续型变量），尽可能地按照这种关系提取原始群落数据中与之相关的模式特征，而不关注其它无关的数据信息。常用的约束排序和有监督学习方法主要包括冗余分析（Redundancy analysis，RDA）、典型相关分析（Canonical analysis）和偏最小二乘判别分析（Partial least squares discriminant analysis，PLS-DA）等。 PLS-DA偏最小二乘法判别分析 PLS-DA（Partial Least Squares Discriminant Analysis）分析是以偏最小二乘回归模型为基础，作为一种有监督的模式识别方法，根据给定的样本分布/分组信息，对群落结构数据进行判别分析。PLS-DA通过寻找物种丰度矩阵和给定的样本分布/分组信息的最大协方差，从而在新的低维坐标系中对样本重新排序。PLS-DA可以减少变量间多重共线性产生的影响，因此，比较适合用于微生物群落数据的研究。 方法步骤 使用R软件，根据物种丰度矩阵和样本分组数据构建PLS-DA判别模型。并计算每个物种的VIP（Variable importance in projection）系数（VIP值需>1，值越大，说明该物种对于组间差异的贡献越大）。 结果说明 **PLS-DA判别分析图** 注：每个点代表一个样本，颜色相同的点属于同一分组，相同分组的点以椭圆标出。如果属于同一分组的样本彼此之间距离越近，同时不同分组的点之间的距离越远，表明分类模型效果越好。 结果目录: C02\_plsda Adonis/PERMANOVA多元方差分析和置换检验 基于置换的PERMANOVA（Permutational multivariate analysis of variance）分析(McArdle and Anderson, 2001)借鉴了ANOVA方差分析多组间差异的统计检验思路，通过对距离矩阵进行置换检验，将原始样本的组间差异与多次随机置换打乱后获得的距离矩阵进行比较，从而评价原始样本组间差异的大小及其统计学显著性。而Adonis分析则可视为PERMANOVA分析的扩展版本，能够对样本检测指标（连续型变量）进行类似的统计检验。 Adonis/PERMANOVA分析将给出一个P值，以表征组间差异是否具有统计学意义。值得注意的是，与ANOVA一样，在多组别的相互比较时，只要其中一组与其它各组中的任意一组具有统计学差异，Adonis/PERMANOVA即给出具有显著性意义的P值。 方法步骤 使用QIIME软件进行Adonis/PERMANOVA分析，并作999次置换检验确定组间差异是否具有统计学显著性。 结果说明 **Unweighted UniFrac距离的Adonis/PERMANOVA分析结果示例图** **Weighted UniFrac距离的Adonis/PERMANOVA分析结果示例图** 注：以上为Adonis/PERMANOVA分析的输出结果，其中需要关注的主要是“Terms added sequentially (first to last)”下方的数据表。表中，“Df”表示自由度，“SumsOfSqs”和“MeanSqs”分别表示离差平方和以及均方差，“F.Model”为F统计量检验值，“R2”表明分组方差以及残差分别占总体方差的比例，即分组所能解释的原始数据中差异的比例。“Pr(>F)”为通过置换检验获得的P值。一般重点关注P值大小即可。P值越小，表明组间差异显著性越强。 结果目录: C03\_adonis ANOSIM相似度分析 ANOSIM（Analysis of similarities）分析也是一种非参数检验方法，通过对样本距离等级排序来判断样本组内和组间差异的大小，并通过置换检验评价原始样本组间差异的统计学显著性(Clarke, 1993; Warton et al., 2012)。 方法步骤 使用QIIME软件进行ANOSIM分析，结果如下所示。 结果说明 **ANOSIM分析结果示例表** | | | | | | --- | --- | --- | --- | | Method name | R statistic | p-value | Number of permutations | | Unweighted UniFrac | 0.1443 | 0.046 | 999 | | Weighted UniFrac | 0.1483 | 0.033 | 999 | 注：R值为ANOSIM的统计量，数值介于-1和1之间，表征组间差异与组内差异的差值大小。R值越接近1，表明组间差异越大，同时组内差异越小，分组效果越好；如果R=0，表明样本的分组效果等同于随机分配，各样本分组之间不具有可观测的统计学差异；如果R为负值，则表明组内差异超过了组间差异的大小，预示分组效果较差。P值则反映了ANOSIM分析结果的统计学显著性，P值越小，表明各样本分组之间的差异显著性越高。 结果目录: C04\_anosim 随机森林分析 随机森林（Random Forests）(Breiman, 2001)是一种基于决策树（Decision tree）的经典高效的机器学习算法，属于非线性分类器（Non-linear classifier），能够深入挖掘变量之间复杂的非线性相互依赖关系，对于经常呈现离散、不连续分布的微生物群落数据而言尤其适用，近几年已有研究证明这一算法能够对微生物群落样本进行有效且准确的分类(Yatsunenko et al., 2012)。 方法步骤 调用R软件的“randomForest”工具包(Liaw and Wiener, 2002)，使用随机森林算法(Breiman, 2001)挑取丰度分布在不同组间存在显著差异的OTU。挑取OTU时使用1000棵随机森林决策树进行建模，并以10倍交叉验证（10-fold cross-validation）估计“基线”误差（Baseline error）的大小（即样本量最大的组别中所有样本全部被错误分类的比率大小，相当于分类模型可能达到的最大错误率）。 结果说明 **衡量随机森林模型效果的关键指标** | | | | --- | --- | | Model | Random Forest | | Error type | 10-fold cross validation | | Estimated error (mean +/- s.d.) | 0.26667 +/- 0.23831 | | Baseline error (for random guessing) | 0.43478 | | Ratio baseline error to observed error | 1.63043 | | Number of trees | 1000 | 注：表中第一行表明分析方法为随即森林，第二行为衡量模型效果和分类准确率的具体方法，一般选用10倍交叉验证。第三行表明建模估计的预报错误率（Estimated error），第四行则为基线错误率，第五行对应两者的比值。表格最后一行表明建模使用的决策树数量，这一参数值越高，建模效果越好。 一般而言，表中第三行的预报错误率越低，相对第四行的基线错误率的改善就越多，模型也就越准确。第五行的比值一般建议应至少> 2，即表明随机森林的分类效果至少相当于“随机猜测”效果的两倍。另一方面，各分组中的样本量大小也会影响到基线错误率值。比如，如果90%同属于一个分组，那么基线错误率为10%，此时即使随机森林能够将分类错误率降低到5%，也依然有较多样本被错误分类；相反，如果数据总体被均分为10个组别，那么基线错误率为90%，此时即使随机森林只能将分类错误率降低到45%，相比“随机猜测”而言，实际上也已经是较大的改善。 **各分组中被错误分类的样本数列表** | | | | | | --- | --- | --- | --- | | True\Predicted | illness | normal | Class error | | illness | 6 | 4 | 0.4 | | normal | 2 | 11 | 0.153846153846154 | 注：上表从第二行开始，每一行代表一种真实的样本分组。第二列开始的每一列则反映了对应分组的样本被正确/错误分类到各分组中的样本数，据此可以计算每组的预报错误率大小，具体数值在最后一列中列出。 **每个OTU的重要度贡献值列表** | | | | | --- | --- | --- | | Feature\_id | Mean\_decrease\_in\_accuracy | Standard\_deviation | | otu749 | 0.00498857864357864 | 0.00306722302796753 | | otu22570 | 0.00469233627483627 | 0.00216391828466729 | | otu3987 | 0.00415501082251082 | 0.00129802828162978 | | otu3338 | 0.00374588744588745 | 0.00191701373937603 | | otu1388 | 0.00333329365079365 | 0.00196251364359776 | | otu578 | 0.00278695887445887 | 0.00188301747912155 | | otu10818 | 0.00255871212121212 | 0.00149862999485973 | | otu11621 | 0.00244314213564214 | 0.00241705946451597 | | otu10547 | 0.0023370670995671 | 0.00154979582389182 | | otu22808 | 0.00210492063492063 | 0.00124257537445842 | | otu2134 | 0.00208075396825397 | 0.000829404048981933 | | otu14930 | 0.00199440476190476 | 0.000687250071640402 | | otu18675 | 0.00196832251082251 | 0.00159692213220206 | | otu3607 | 0.00190549422799423 | 0.00119662635417662 | | otu20709 | 0.0018473556998557 | 0.00102618277527416 | | otu2586 | 0.00180516955266955 | 0.0013539001931897 | | otu6601 | 0.00173961038961039 | 0.00227938970173076 | | otu14203 | 0.00166635642135642 | 0.0020090542065586 | | otu1403 | 0.00163727994227994 | 0.000775566630065235 | | otu13524 | 0.00149416666666667 | 0.000887504493439631 | | otu7744 | 0.00146896825396825 | 0.00104955300220483 | | otu20428 | 0.00137203823953824 | 0.00113734919410948 | | otu21801 | 0.00123945165945166 | 0.00106626034333815 | | otu8677 | 0.00121866161616162 | 0.00092861661611077 | | otu17338 | 0.00119723665223665 | 0.00162295872689325 | | otu8197 | 0.0011505303030303 | 0.000758788530289155 | | otu15649 | 0.000980887445887446 | 0.000569254925406034 | | otu12057 | 0.000891984126984127 | 0.000899771790631524 | | otu10243 | 0.000831717171717172 | 0.000661876406439635 | | otu7326 | 0.000754206349206349 | 0.000715482688043774 | | otu12713 | 0.000750436507936508 | 0.000748271536686374 | | otu18994 | 0.000746038961038961 | 0.000629159167677939 | | otu10983 | 0.000727896825396825 | 0.000758872452997745 | | otu15192 | 0.000707417027417027 | 0.00064685456921782 | | otu6643 | 0.000646587301587302 | 0.000438748187466641 | | otu7591 | 0.000644242424242424 | 0.000653796211622001 | | otu23172 | 0.000630858585858586 | 0.000812075814046012 | | otu6546 | 0.000588769841269841 | 0.000508445376230609 | | otu13401 | 0.000587936507936508 | 0.000967178820200481 | | otu7808 | 0.000585634920634921 | 0.00033608363986359 | | otu11694 | 0.000581071428571429 | 0.000811891272444534 | | otu3663 | 0.000574166666666667 | 0.000649772003446274 | | otu13693 | 0.00056695165945166 | 0.000550219920957319 | | otu10971 | 0.000557388167388167 | 0.000624282239679539 | | otu20568 | 0.000550357142857143 | 0.000750738754373759 | | otu13026 | 0.000541706349206349 | 0.000866139958924337 | | otu13340 | 0.000513373015873016 | 0.000395288206540429 | | otu15538 | 0.0005070670995671 | 0.000503946169884071 | | otu10907 | 0.000487420634920635 | 0.000508124002611192 | | otu20140 | 0.000487420634920635 | 0.000620695418335885 | | otu12869 | 0.00048 | 0.000872349737843577 | | otu9087 | 0.000474920634920635 | 0.000527529605759552 | | otu22610 | 0.00046290404040404 | 0.000465130235737321 | | otu23424 | 0.000460119047619048 | 0.000810659805909771 | | otu6891 | 0.000453376623376623 | 0.000635804960476077 | | otu17047 | 0.000450364357864358 | 0.000500221574782715 | | otu5326 | 0.000436071428571429 | 0.000972787123974073 | | otu10489 | 0.000406071428571429 | 0.000692554799929673 | | otu19057 | 0.00040047619047619 | 0.0005310663481001 | | otu3415 | 0.000378694083694084 | 0.000495853690315769 | | otu19644 | 0.000372676767676768 | 0.00073832935020652 | | otu20431 | 0.00036765873015873 | 0.000322115626997969 | | otu4472 | 0.000348134920634921 | 0.000593741830453345 | | otu5176 | 0.000338134920634921 | 0.000532059137134232 | | otu6416 | 0.000328928571428571 | 0.00036488931148855 | | otu21832 | 0.000318373015873016 | 0.000363080518875526 | | otu7996 | 0.000314365079365079 | 0.000266535527194398 | | otu2398 | 0.000304325396825397 | 0.000237918250649944 | | otu11944 | 0.000301230158730159 | 0.000339812134803999 | | otu6655 | 0.000298257575757576 | 0.000467096446929148 | | otu3847 | 0.00028202380952381 | 0.000554493979767286 | | otu13703 | 0.000280595238095238 | 0.000357355338027191 | | otu13138 | 0.000274365079365079 | 0.00026638176863597 | | otu13224 | 0.000272539682539683 | 0.000293691355223335 | | otu12861 | 0.000271468253968254 | 0.000214165363191799 | | otu23630 | 0.000270039682539683 | 0.000682617588402807 | | otu6703 | 0.000267063492063492 | 0.00063483947342811 | | otu19039 | 0.000266071428571429 | 0.000260913243271673 | | otu2551 | 0.000259484126984127 | 0.000375185929943247 | | otu275 | 0.000258849206349206 | 0.000306339157043826 | | otu10707 | 0.000251547619047619 | 0.000405810898624885 | | otu10959 | 0.000250800865800866 | 0.000526395487687737 | | otu13303 | 0.000245634920634921 | 0.000375846980752602 | | otu19181 | 0.000242222222222222 | 0.00023851104198082 | | otu22023 | 0.000233412698412698 | 0.00053827673787082 | | otu4895 | 0.000219761904761905 | 0.000507132361965079 | | otu12517 | 0.000219126984126984 | 0.000295527441608857 | | otu7794 | 0.000216865079365079 | 0.00024774685586529 | | otu11524 | 0.000214761904761905 | 0.000509873840659945 | | otu10622 | 0.000211507936507937 | 0.000263131446669252 | | otu18482 | 0.000210241702741703 | 0.000571944303683759 | | otu569 | 0.000205119047619048 | 0.000793684577102837 | | otu21544 | 0.000194761904761905 | 0.000397498626450822 | | otu10710 | 0.000194087301587302 | 0.000267960065796734 | | otu7432 | 0.000193614718614719 | 0.000205327163076048 | | otu1220 | 0.000180198412698413 | 0.000207869556011852 | | otu12558 | 0.00017468253968254 | 0.000373602666153488 | | otu23769 | 0.000174404761904762 | 0.000365045156456492 | | otu14275 | 0.000171031746031746 | 0.00038134730227994 | | otu18708 | 0.00017031746031746 | 0.000595142373255715 | | otu8299 | 0.000169404761904762 | 0.000172002176780477 | | otu20294 | 0.000168611111111111 | 0.000319922137272968 | | otu12246 | 0.000167698412698413 | 0.000235970546393892 | | otu2858 | 0.000165948773448773 | 0.000372341962607285 | | otu2364 | 0.000164917027417027 | 0.000347120575794681 | | otu1832 | 0.00016484126984127 | 0.000476572899056497 | | otu14602 | 0.000164007936507936 | 0.000416275706813108 | | otu19892 | 0.000163888888888889 | 0.00024443392233246 | | otu3533 | 0.000163683261183261 | 0.000572662658262068 | | otu3317 | 0.000162777777777778 | 0.000205621835650332 | | otu17129 | 0.00016234126984127 | 0.000276528207953978 | | otu22053 | 0.000159325396825397 | 0.000421274980148984 | | otu12026 | 0.000159242424242424 | 0.0003639815994544 | | otu10967 | 0.000146706349206349 | 0.000445230748523894 | | otu3720 | 0.000145865800865801 | 0.000381173462466072 | | otu11739 | 0.000145205627705628 | 0.000317074256768022 | | otu5610 | 0.000143571428571429 | 0.000211754747446813 | | otu5182 | 0.000143333333333333 | 0.00035540990497494 | | otu13223 | 0.000139671717171717 | 0.000437064526893786 | | otu20828 | 0.000137063492063492 | 0.00019674316994375 | | otu22301 | 0.000136666666666667 | 0.000178652362611488 | | otu3313 | 0.000135555555555556 | 0.000472010319565734 | | otu6455 | 0.000134722222222222 | 0.000259021274126577 | | otu12088 | 0.000134126984126984 | 0.000172808234222408 | | otu7969 | 0.000132539682539683 | 0.000259143222602008 | | otu20727 | 0.000131428571428571 | 0.000361296965565036 | | otu6338 | 0.000131313131313131 | 0.00031758409629414 | | otu7837 | 0.000131071428571429 | 0.000295884661272324 | | otu1328 | 0.000130555555555556 | 0.000368264325859327 | | otu7119 | 0.00013 | 0.000191351987892327 | | otu23589 | 0.000128928571428571 | 0.000319398867891356 | | otu1330 | 0.000123134920634921 | 0.000754714520912697 | | otu10552 | 0.00012281746031746 | 0.000324263590520561 | | otu14895 | 0.000122698412698413 | 0.000113794874825605 | | otu6656 | 0.000120039682539683 | 0.000371501544049712 | | otu12073 | 0.000113611111111111 | 0.000151995471829887 | | otu13725 | 0.000113293650793651 | 0.00017329165620524 | | otu1431 | 0.000113095238095238 | 0.000255051015305102 | | otu15666 | 0.000112896825396825 | 0.000210410385946757 | | otu12078 | 0.000110555555555556 | 0.000188497233754395 | | otu23733 | 0.00010968253968254 | 0.000191136257112743 | | otu2809 | 0.000108095238095238 | 0.000159806667019512 | | otu10695 | 0.000107380952380952 | 0.000413424659459954 | | otu16218 | 0.000106428571428571 | 0.000192855673127851 | | otu19605 | 0.000106150793650794 | 0.000155504172352537 | | otu1438 | 0.000104761904761905 | 0.000170826560691827 | | otu14526 | 0.000103293650793651 | 0.000121792151160574 | | otu14702 | 0.00010297619047619 | 0.000232203051482236 | | otu9548 | 0.000102738095238095 | 0.00013519585607974 | | otu18692 | 1e-04 | 0.000164991582276861 | | otu12640 | 9.8452380952381e-05 | 0.000137860192690446 | | otu1341 | 9.62301587301587e-05 | 0.000119901588765513 | | otu23368 | 9.61111111111111e-05 | 0.000261017268695508 | | otu16663 | 9.23376623376623e-05 | 0.000189504061985996 | | otu5341 | 9.07611832611833e-05 | 0.000336018484524113 | | otu20349 | 8.88492063492063e-05 | 0.000191817591685866 | | otu15930 | 8.8452380952381e-05 | 0.000119670394840832 | | otu15318 | 8.82936507936508e-05 | 0.000169367059761409 | | otu21136 | 8.50793650793651e-05 | 0.000179158470694431 | | otu6440 | 8.45238095238095e-05 | 0.000115890681410512 | | otu22383 | 8.38095238095238e-05 | 0.000114774088795362 | | otu16590 | 8.21031746031746e-05 | 0.000191041420500955 | | otu10888 | 8.20634920634921e-05 | 0.000334817902195814 | | otu3336 | 8.05555555555556e-05 | 0.000148984905469021 | | otu14482 | 7.95238095238095e-05 | 0.000156653880888988 | | otu10585 | 7.90079365079365e-05 | 0.000238739314767053 | | otu23030 | 7.61904761904762e-05 | 0.000108381694214344 | | otu9799 | 7.35714285714286e-05 | 9.95482995614603e-05 | | otu7889 | 7.31746031746032e-05 | 9.66226518155725e-05 | | otu14096 | 7.21031746031746e-05 | 0.000327574393707931 | | otu7874 | 7.18253968253968e-05 | 0.000279506525339268 | | otu15713 | 7.11904761904762e-05 | 0.00031548892967302 | | otu11470 | 7.06746031746032e-05 | 9.40595156154558e-05 | | otu19410 | 7.06349206349206e-05 | 0.000119864919352613 | | otu10201 | 6.96031746031746e-05 | 0.000551010546928921 | | otu16932 | 6.94444444444444e-05 | 0.000448786697679418 | | otu20840 | 6.8452380952381e-05 | 0.000440228104739543 | | otu21778 | 6.8452380952381e-05 | 0.000113121342016669 | | otu19065 | 6.81818181818182e-05 | 0.000148546599682158 | | otu2424 | 6.80555555555556e-05 | 0.000120094056074923 | | otu98 | 6.78571428571429e-05 | 0.000170408672450256 | | otu6448 | 6.78571428571428e-05 | 0.000114743626285919 | | otu16574 | 6.76984126984127e-05 | 0.000367337792547785 | | otu12299 | 6.70634920634921e-05 | 0.000162202431516262 | | otu20224 | 6.67857142857143e-05 | 0.00016092182367017 | | otu14823 | 6.6468253968254e-05 | 0.00013218664368215 | | otu20812 | 6.57936507936508e-05 | 0.00015263814366093 | | otu19500 | 6.5e-05 | 0.000128668393770792 | | otu12718 | 6.3452380952381e-05 | 8.40296869219597e-05 | | otu2741 | 6.28571428571428e-05 | 0.000106734672199024 | | otu18345 | 6.11111111111111e-05 | 0.000105409255338946 | | otu12976 | 6.06349206349206e-05 | 0.000162748174951154 | | otu20334 | 6.01190476190476e-05 | 0.000146944664128877 | | otu11725 | 6e-05 | 0.000134989711542111 | | otu15125 | 5.99206349206349e-05 | 0.000634029218811359 | | otu12016 | 5.87301587301587e-05 | 0.000142179462581904 | | otu8080 | 5.87301587301587e-05 | 0.000117133345374097 | | otu11777 | 5.8531746031746e-05 | 0.000343567806187599 | | otu611 | 5.8452380952381e-05 | 0.000267818309942277 | | otu1497 | 5.8452380952381e-05 | 0.000170093481865109 | | otu19592 | 5.83333333333333e-05 | 0.000124536176508111 | | otu7611 | 5.78968253968254e-05 | 0.000132570628931633 | | otu1169 | 5.76190476190476e-05 | 0.000272029062850875 | | otu21271 | 5.69444444444444e-05 | 0.000145292205926129 | | otu7840 | 5.69444444444444e-05 | 9.54310306905238e-05 | | otu3957 | 5.58333333333333e-05 | 0.000108301277308565 | | otu20592 | 5.55555555555556e-05 | 0.000120013716637183 | | otu7018 | 5.51984126984127e-05 | 0.000227611370643084 | | otu8848 | 5.41666666666667e-05 | 0.000171290039925787 | | otu883 | 5.35714285714286e-05 | 0.000113251771371669 | | otu3899 | 5.28571428571429e-05 | 0.000135701753807364 | | otu17667 | 5.27380952380952e-05 | 0.000214323960431673 | | otu9391 | 5.23412698412699e-05 | 0.000463985735330983 | | otu20300 | 5.23412698412698e-05 | 0.000467666374592476 | | otu62 | 5.17857142857143e-05 | 0.000126114644780525 | | otu21590 | 5.11904761904762e-05 | 0.000277301493919013 | | otu19404 | 5.05952380952381e-05 | 0.00019446975053985 | | otu11884 | 5.0515873015873e-05 | 0.000251854634199428 | | otu8632 | 5.01623376623377e-05 | 6.62848970086498e-05 | | otu1770 | 5e-05 | 0.000148610389983251 | | otu9246 | 4.98015873015873e-05 | 0.000189468717343439 | | otu8388 | 4.92857142857143e-05 | 8.73163768136536e-05 | | otu135 | 4.90079365079365e-05 | 0.00025356844425576 | | otu19055 | 4.86111111111111e-05 | 0.000179454142993651 | | otu14979 | 4.8015873015873e-05 | 0.000133832760163398 | | otu10995 | 4.76190476190476e-05 | 0.000109971479845643 | | otu19275 | 4.76190476190476e-05 | 0.000145478593490662 | | otu14678 | 4.76190476190476e-05 | 0.000109971479845643 | | otu19856 | 4.75757575757576e-05 | 0.000186311850388596 | | otu10775 | 4.61904761904762e-05 | 0.000151153288764947 | | otu16775 | 4.57539682539682e-05 | 0.000372264292643986 | | otu12708 | 4.56746031746032e-05 | 0.000139043810045435 | | otu13793 | 4.56637806637807e-05 | 0.000384038552164207 | | otu15689 | 4.52380952380952e-05 | 7.31279333319965e-05 | | otu14363 | 4.52380952380952e-05 | 9.94124842547412e-05 | | otu9072 | 4.45634920634921e-05 | 0.000179934251878346 | | otu5300 | 4.44444444444444e-05 | 0.000135735814169604 | | otu14800 | 4.30555555555556e-05 | 0.00019183098163332 | | otu41 | 4.27380952380952e-05 | 0.000145907091810731 | | otu15799 | 4.26587301587302e-05 | 0.000154691935750195 | | otu10336 | 4.16666666666667e-05 | 6.80413817439772e-05 | | otu5705 | 4.11111111111111e-05 | 0.000105934990547138 | | otu12052 | 4.08369408369408e-05 | 0.000121082353404441 | | otu1408 | 4e-05 | 0.000126491106406735 | | otu10255 | 4e-05 | 0.000126491106406735 | | otu6231 | 3.98412698412698e-05 | 0.000180429021305429 | | otu982 | 3.96825396825397e-05 | 0.000150270604990171 | | otu337 | 3.9047619047619e-05 | 0.000208801665583674 | | otu7971 | 3.9047619047619e-05 | 0.00051170310343751 | | otu15213 | 3.88888888888889e-05 | 8.30241176720737e-05 | | otu17574 | 3.82936507936508e-05 | 0.000121095156431051 | | otu14885 | 3.77380952380952e-05 | 0.000218490368204853 | | otu11775 | 3.75396825396825e-05 | 0.00027082584409928 | | otu21752 | 3.75e-05 | 8.43685697138191e-05 | | otu14392 | 3.67857142857143e-05 | 7.9931411924505e-05 | | otu1631 | 3.65079365079365e-05 | 0.000243749369668969 | | otu4648 | 3.65079365079365e-05 | 0.000115448232037893 | | otu4912 | 3.61111111111111e-05 | 0.000114193359950525 | | otu17192 | 3.42857142857143e-05 | 7.35247964404525e-05 | | otu3972 | 3.42857142857143e-05 | 7.35247964404525e-05 | | otu7282 | 3.37301587301587e-05 | 9.10539198855854e-05 | | otu22957 | 3.33333333333333e-05 | 0.000157173250761757 | | otu1803 | 3.33333333333333e-05 | 7.02728368926307e-05 | | otu11836 | 3.33333333333333e-05 | 7.02728368926307e-05 | | otu14691 | 3.33333333333333e-05 | 0.000105409255338946 | | otu4586 | 3.33333333333333e-05 | 0.000105409255338946 | | otu22211 | 3.33333333333333e-05 | 0.000105409255338946 | | otu11219 | 3.33333333333333e-05 | 0.000105409255338946 | | otu6419 | 3.33333333333333e-05 | 7.49942841900614e-05 | | otu9123 | 3.28968253968254e-05 | 0.000107160565849886 | | otu16491 | 3.13492063492064e-05 | 0.000162705510233626 | | otu12205 | 3.11111111111111e-05 | 0.000124809869118413 | | otu23512 | 3.0952380952381e-05 | 6.54942255104056e-05 | | otu15727 | 3.07936507936508e-05 | 0.000147116970605802 | | otu17900 | 3.03571428571429e-05 | 8.66884130226234e-05 | | otu10811 | 3.03571428571429e-05 | 0.000109772621106864 | | otu592 | 3e-05 | 0.000123178080461472 | | otu16277 | 2.92929292929293e-05 | 6.39642028241922e-05 | | otu13302 | 2.91666666666667e-05 | 0.000163363938640166 | | otu3667 | 2.91666666666667e-05 | 9.22330984215777e-05 | | otu12498 | 2.88492063492063e-05 | 0.000171845511854603 | | otu13578 | 2.85714285714286e-05 | 0.000149793046114121 | | otu7725 | 2.85714285714286e-05 | 9.03507902905251e-05 | | otu9688 | 2.85714285714286e-05 | 9.03507902905251e-05 | | otu10806 | 2.85714285714286e-05 | 9.03507902905251e-05 | | otu10105 | 2.85714285714286e-05 | 9.03507902905251e-05 | | otu4350 | 2.85714285714286e-05 | 9.03507902905251e-05 | | otu11289 | 2.85714285714286e-05 | 9.03507902905251e-05 | | otu967 | 2.85714285714286e-05 | 9.03507902905251e-05 | | otu19653 | 2.85714285714286e-05 | 9.03507902905251e-05 | | otu20772 | 2.85714285714286e-05 | 9.03507902905251e-05 | | otu3166 | 2.85714285714286e-05 | 9.03507902905251e-05 | | otu17146 | 2.85714285714286e-05 | 9.03507902905251e-05 | | otu13814 | 2.85714285714286e-05 | 9.03507902905251e-05 | | otu4789 | 2.85714285714286e-05 | 9.03507902905251e-05 | | otu19970 | 2.85714285714286e-05 | 9.03507902905251e-05 | | otu21319 | 2.85714285714286e-05 | 9.03507902905251e-05 | | otu16754 | 2.81746031746032e-05 | 0.000232612129134118 | | otu4448 | 2.77777777777778e-05 | 9.53299087128426e-05 | | otu13606 | 2.77777777777778e-05 | 6.00068583185913e-05 | | otu18499 | 2.75793650793651e-05 | 0.000144865276694253 | | otu15947 | 2.73809523809524e-05 | 0.00010707669915128 | | otu14517 | 2.70634920634921e-05 | 0.000311521770326061 | | otu4401 | 2.69011544011544e-05 | 0.000126368351770765 | | otu23662 | 2.67857142857143e-05 | 8.47038658973673e-05 | | otu2654 | 2.67857142857143e-05 | 5.66258856858347e-05 | | otu12922 | 2.67857142857143e-05 | 5.66258856858347e-05 | | otu23795 | 2.67857142857143e-05 | 0.00011080065755349 | | otu22085 | 2.66666666666667e-05 | 0.000158369608147007 | | otu3049 | 2.66666666666667e-05 | 5.83730023847275e-05 | | otu19951 | 2.53968253968254e-05 | 5.4061544018814e-05 | | otu16726 | 2.53968253968254e-05 | 5.4061544018814e-05 | | otu7859 | 2.53968253968254e-05 | 5.4061544018814e-05 | | otu1596 | 2.5e-05 | 7.90569415042095e-05 | | otu19071 | 2.5e-05 | 7.90569415042095e-05 | | otu4394 | 2.5e-05 | 7.90569415042095e-05 | | otu4433 | 2.5e-05 | 7.90569415042095e-05 | | otu10506 | 2.5e-05 | 7.90569415042095e-05 | | otu10665 | 2.5e-05 | 7.90569415042095e-05 | | otu13110 | 2.5e-05 | 7.90569415042095e-05 | | otu10928 | 2.5e-05 | 7.90569415042095e-05 | | otu14597 | 2.5e-05 | 7.90569415042095e-05 | | otu21032 | 2.5e-05 | 7.90569415042095e-05 | | otu21883 | 2.5e-05 | 7.90569415042095e-05 | | otu15525 | 2.5e-05 | 0.000124536176508111 | | otu13805 | 2.46031746031746e-05 | 0.000116793024471557 | | otu20805 | 2.44444444444444e-05 | 9.81027148512196e-05 | | otu1083 | 2.42857142857143e-05 | 5.21857835916094e-05 | | otu11722 | 2.41666666666667e-05 | 9.28334829442241e-05 | | otu8893 | 2.33333333333333e-05 | 0.000142335024710221 | | otu5642 | 2.27777777777778e-05 | 0.000260470472731504 | | otu19295 | 2.22222222222222e-05 | 0.000140454142890639 | | otu5988 | 2.22222222222222e-05 | 7.02728368926306e-05 | | otu4477 | 2.22222222222222e-05 | 7.02728368926306e-05 | | otu12255 | 2.22222222222222e-05 | 7.02728368926306e-05 | | otu9189 | 2.22222222222222e-05 | 0.000105409255338946 | | otu13028 | 2.22222222222222e-05 | 7.02728368926306e-05 | | otu13183 | 2.22222222222222e-05 | 7.02728368926306e-05 | | otu259 | 2.22222222222222e-05 | 9.35597635486602e-05 | | otu12276 | 2.19444444444444e-05 | 0.000216826262680287 | | otu18036 | 2.11111111111111e-05 | 4.45831169949753e-05 | | otu9419 | 2.09920634920635e-05 | 0.000206344920081653 | | otu12101 | 2.09523809523809e-05 | 0.000206623139297422 | | otu11925 | 2.08333333333333e-05 | 0.000116617052942685 | | otu12953 | 2e-05 | 6.32455532033676e-05 | | otu14010 | 2e-05 | 6.32455532033676e-05 | | otu9231 | 2e-05 | 6.32455532033676e-05 | | otu5064 | 2e-05 | 6.32455532033676e-05 | | otu12008 | 2e-05 | 6.32455532033676e-05 | | otu1064 | 2e-05 | 6.32455532033676e-05 | | otu7648 | 2e-05 | 6.32455532033676e-05 | | otu4550 | 1.98809523809524e-05 | 0.000228163710345008 | | otu1159 | 1.94444444444444e-05 | 5.36717657266275e-05 | | otu7232 | 1.94444444444444e-05 | 0.000109500323377084 | | otu22225 | 1.82142857142857e-05 | 0.000236695654969374 | | otu230 | 1.79365079365079e-05 | 9.22398420454492e-05 | | otu9376 | 1.78571428571429e-05 | 0.000164311592164789 | | otu6341 | 1.73737373737374e-05 | 0.000422714427996753 | | otu18826 | 1.7063492063492e-05 | 0.000218845278833836 | | otu19554 | 1.66666666666667e-05 | 5.2704627669473e-05 | | otu19798 | 1.66666666666667e-05 | 8.55156428384189e-05 | | otu20228 | 1.66666666666667e-05 | 5.2704627669473e-05 | | otu19528 | 1.66666666666667e-05 | 5.2704627669473e-05 | | otu8062 | 1.66666666666667e-05 | 0.000122977464562104 | | otu22756 | 1.66666666666667e-05 | 5.2704627669473e-05 | | otu3635 | 1.66666666666667e-05 | 5.2704627669473e-05 | | otu23483 | 1.66666666666667e-05 | 5.2704627669473e-05 | | otu11690 | 1.66666666666667e-05 | 5.2704627669473e-05 | | otu4435 | 1.66666666666667e-05 | 5.2704627669473e-05 | | otu22595 | 1.66666666666667e-05 | 5.2704627669473e-05 | | otu21767 | 1.66666666666667e-05 | 5.2704627669473e-05 | | otu2218 | 1.66666666666667e-05 | 5.2704627669473e-05 | | otu8058 | 1.66666666666667e-05 | 5.2704627669473e-05 | | otu17949 | 1.66666666666667e-05 | 5.2704627669473e-05 | | otu11750 | 1.66666666666667e-05 | 5.2704627669473e-05 | | otu12825 | 1.66666666666667e-05 | 5.2704627669473e-05 | | otu17107 | 1.62301587301587e-05 | 0.000222907300170003 | | otu3174 | 1.60714285714286e-05 | 0.000102564204768395 | | otu18276 | 1.60714285714286e-05 | 0.000102564204768395 | | otu13943 | 1.60714285714286e-05 | 0.000102564204768395 | | otu15810 | 1.60714285714286e-05 | 7.73580552204035e-05 | | otu3163 | 1.42857142857143e-05 | 4.51753951452626e-05 | | otu19660 | 1.42857142857143e-05 | 4.51753951452626e-05 | | otu1044 | 1.42857142857143e-05 | 4.51753951452626e-05 | | otu9710 | 1.42857142857143e-05 | 4.51753951452626e-05 | | otu13369 | 1.42857142857143e-05 | 4.51753951452626e-05 | | otu9690 | 1.42857142857143e-05 | 4.51753951452626e-05 | | otu19950 | 1.42857142857143e-05 | 4.51753951452626e-05 | | otu6136 | 1.42857142857143e-05 | 0.000209063346843002 | | otu7395 | 1.42857142857143e-05 | 4.51753951452626e-05 | | otu14425 | 1.42857142857143e-05 | 4.51753951452626e-05 | | otu18716 | 1.42857142857143e-05 | 4.51753951452626e-05 | | otu14317 | 1.42857142857143e-05 | 4.51753951452626e-05 | | otu11478 | 1.42857142857143e-05 | 4.51753951452626e-05 | | otu12296 | 1.42857142857143e-05 | 6.91686384967661e-05 | | otu21198 | 1.42857142857143e-05 | 4.51753951452626e-05 | | otu4924 | 1.42857142857143e-05 | 4.51753951452626e-05 | | otu12346 | 1.42857142857143e-05 | 4.51753951452626e-05 | | otu13903 | 1.42857142857143e-05 | 4.51753951452626e-05 | | otu6826 | 1.42857142857143e-05 | 4.51753951452626e-05 | | otu18915 | 1.42857142857143e-05 | 4.51753951452626e-05 | | otu6158 | 1.42857142857143e-05 | 4.51753951452626e-05 | | otu23178 | 1.42857142857143e-05 | 4.51753951452626e-05 | | otu12614 | 1.42857142857143e-05 | 4.51753951452626e-05 | | otu22793 | 1.42857142857143e-05 | 4.51753951452626e-05 | | otu2251 | 1.42857142857143e-05 | 4.51753951452626e-05 | | otu4474 | 1.42857142857143e-05 | 8.10923160282209e-05 | | otu4082 | 1.40873015873016e-05 | 0.000127109623007162 | | otu20893 | 1.38888888888889e-05 | 9.0010287477887e-05 | | otu5931 | 1.38888888888889e-05 | 9.0010287477887e-05 | | otu8990 | 1.3531746031746e-05 | 0.000377930606619571 | | otu2500 | 1.34920634920635e-05 | 9.99784767296835e-05 | | otu20809 | 1.25e-05 | 3.95284707521047e-05 | | otu2070 | 1.25e-05 | 3.95284707521047e-05 | | otu2040 | 1.25e-05 | 3.95284707521047e-05 | | otu14382 | 1.25e-05 | 3.95284707521047e-05 | | otu8920 | 1.25e-05 | 3.95284707521047e-05 | | otu17115 | 1.25e-05 | 3.95284707521047e-05 | | otu18541 | 1.25e-05 | 3.95284707521047e-05 | | otu13801 | 1.25e-05 | 3.95284707521047e-05 | | otu11553 | 1.25e-05 | 3.95284707521047e-05 | | otu13231 | 1.25e-05 | 3.95284707521047e-05 | | otu13207 | 1.25e-05 | 9.22330984215777e-05 | | otu14948 | 1.25e-05 | 3.95284707521047e-05 | | otu19681 | 1.25e-05 | 3.95284707521047e-05 | | otu21768 | 1.25e-05 | 3.95284707521047e-05 | | otu21336 | 1.25e-05 | 3.95284707521047e-05 | | otu5915 | 1.25e-05 | 3.95284707521047e-05 | | otu21432 | 1.25e-05 | 3.95284707521047e-05 | | otu1535 | 1.25e-05 | 3.95284707521047e-05 | | otu10674 | 1.25e-05 | 3.95284707521047e-05 | | otu7210 | 1.25e-05 | 3.95284707521047e-05 | | otu6468 | 1.25e-05 | 3.95284707521047e-05 | | otu22297 | 1.25e-05 | 3.95284707521047e-05 | | otu18778 | 1.22222222222222e-05 | 8.02003663671937e-05 | | otu7484 | 1.17857142857143e-05 | 9.74454030519845e-05 | | otu12733 | 1.15079365079365e-05 | 6.222416807597e-05 | | otu3065 | 1.11111111111111e-05 | 3.51364184463153e-05 | | otu16498 | 1.11111111111111e-05 | 3.51364184463153e-05 | | otu22952 | 1.11111111111111e-05 | 3.51364184463153e-05 | | otu1423 | 1.11111111111111e-05 | 3.51364184463153e-05 | | otu5946 | 1.11111111111111e-05 | 3.51364184463153e-05 | | otu558 | 1.11111111111111e-05 | 3.51364184463153e-05 | | otu13460 | 1.11111111111111e-05 | 3.51364184463153e-05 | | otu10164 | 1.11111111111111e-05 | 3.51364184463153e-05 | | otu3187 | 1.11111111111111e-05 | 0.00013919467471996 | | otu3970 | 1.11111111111111e-05 | 3.51364184463153e-05 | | otu6227 | 1.11111111111111e-05 | 3.51364184463153e-05 | | otu13920 | 1.11111111111111e-05 | 3.51364184463153e-05 | | otu11179 | 1.11111111111111e-05 | 3.51364184463153e-05 | | otu16556 | 1.11111111111111e-05 | 8.19849763747357e-05 | | otu18317 | 1.11111111111111e-05 | 3.51364184463153e-05 | | otu9388 | 1.11111111111111e-05 | 3.51364184463153e-05 | | otu18806 | 1.11111111111111e-05 | 3.51364184463153e-05 | | otu10458 | 1.08730158730159e-05 | 0.000350023604766176 | | otu2966 | 1.07936507936508e-05 | 0.000163885397813106 | | otu10 | 1.07142857142857e-05 | 9.53124709583243e-05 | | otu15272 | 1.07142857142857e-05 | 9.53124709583243e-05 | | otu7954 | 1.07142857142857e-05 | 0.00042277925853491 | | otu19176 | 1.0515873015873e-05 | 0.000253274049152559 | | otu13364 | 1e-05 | 3.16227766016838e-05 | | otu3211 | 1e-05 | 3.16227766016838e-05 | | otu8695 | 1e-05 | 3.16227766016838e-05 | | otu18910 | 9.79437229437229e-06 | 0.000369773690125323 | | otu15832 | 9.72222222222222e-06 | 6.6140542261081e-05 | | otu8850 | 8.61111111111111e-06 | 7.0058397157983e-05 | | otu21830 | 8.33333333333333e-06 | 9.97682499781554e-05 | | otu16098 | 7.93650793650794e-06 | 8.76615953745021e-05 | | otu9877 | 7.81746031746031e-06 | 0.000201109020484993 | | otu5061 | 7.57575757575757e-06 | 6.27767822962427e-05 | | otu11704 | 6.78571428571429e-06 | 0.000319100450691699 | | otu6197 | 6.78571428571428e-06 | 4.52521907864697e-05 | | otu21647 | 6.66666666666667e-06 | 0.00017270541535756 | | otu3756 | 5.95238095238095e-06 | 0.000110720685938918 | | otu16677 | 5.71428571428572e-06 | 8.17051831981989e-05 | | otu8719 | 5.71428571428572e-06 | 8.17051831981989e-05 | | otu789 | 5.71428571428571e-06 | 0.00013468326458261 | | otu13879 | 5.55555555555556e-06 | 6.65121666521036e-05 | | otu13545 | 5.55555555555555e-06 | 0.000111742349025415 | | otu22263 | 5.16233766233766e-06 | 0.000127041383707892 | | otu11531 | 4.28571428571428e-06 | 5.79506397202907e-05 | | otu12886 | 4.16666666666667e-06 | 6.93054163881925e-05 | | otu10194 | 4.16666666666667e-06 | 0.000127604202820579 | | otu64 | 4.16666666666667e-06 | 6.93054163881925e-05 | | otu6773 | 3.57142857142857e-06 | 0.000112246186345935 | | otu4945 | 3.45238095238094e-06 | 0.000329327044084191 | | otu13731 | 3.40909090909091e-06 | 5.13952481608659e-05 | | otu14239 | 3.40909090909091e-06 | 5.13952481608659e-05 | | otu9827 | 3.33333333333334e-06 | 1.05409255338946e-05 | | otu9067 | 3.17460317460318e-06 | 0.000319296113973254 | | otu17614 | 3.17460317460317e-06 | 1.00389766989472e-05 | | otu12584 | 3.17460317460317e-06 | 6.02338601936834e-05 | | otu16778 | 3.17460317460317e-06 | 0.000196814995884943 | | otu23403 | 3.17460317460317e-06 | 0.000174891536772563 | | otu17301 | 3.0952380952381e-06 | 0.000324609578079425 | | otu9073 | 2.5e-06 | 0.000123855875023262 | | otu169 | 2.5e-06 | 5.32942565177316e-05 | | otu23034 | 2.5e-06 | 7.90569415042095e-06 | | otu9573 | 2.38095238095238e-06 | 7.31279333319965e-05 | | otu1539 | 2.38095238095238e-06 | 7.52923252421043e-06 | | otu3560 | 2.14285714285715e-06 | 0.000178302972811388 | | otu18303 | 1.85064935064936e-06 | 0.000332610423626103 | | otu7531 | 1.78571428571429e-06 | 5.48459499989973e-05 | | otu3124 | 1.78571428571428e-06 | 5.64692439315782e-06 | | otu14786 | 1.78571428571428e-06 | 6.324667361495e-05 | | otu5363 | 1.78571428571428e-06 | 6.324667361495e-05 | | otu23144 | 1.51515151515152e-06 | 8.2200751670197e-05 | | otu11221 | 1.38888888888889e-06 | 5.57288962437191e-05 | | otu14104 | 9.92063492063484e-07 | 0.000354442132732008 | | otu15266 | 7.93650793650798e-08 | 0.000214065289334253 | | otu17402 | 1.01643953670516e-21 | 6.35540372144721e-05 | | otu23176 | 0 | 0 | | otu22009 | 0 | 0 | | otu16836 | 0 | 0 | | otu11892 | 0 | 0 | | otu1360 | 0 | 0 | | otu1207 | 0 | 0 | | otu12525 | 0 | 0 | | otu13880 | 0 | 0 | | otu507 | 0 | 0 | | otu8138 | 0 | 0 | | otu7845 | 0 | 0 | | otu9477 | 0 | 0 | | otu7310 | 0 | 0 | | otu2818 | 0 | 0 | | otu18649 | 0 | 0 | | otu11066 | 0 | 0 | | otu17847 | 0 | 0 | | otu20609 | 0 | 0 | | otu6474 | 0 | 0 | | otu4475 | 0 | 0 | | otu15380 | 0 | 0 | | otu22795 | 0 | 0 | | otu11184 | 0 | 0 | | otu5701 | 0 | 0 | | otu20400 | 0 | 0 | | otu13753 | 0 | 0 | | otu16022 | 0 | 0 | | otu23855 | 0 | 0 | | otu18678 | 0 | 0 | | otu7514 | 0 | 0 | | otu3180 | 0 | 0 | | otu4495 | 0 | 0 | | otu20729 | 0 | 0 | | otu15578 | 0 | 0 | | otu16374 | 0 | 0 | | otu22408 | 0 | 0 | | otu13721 | 0 | 0 | | otu8693 | 0 | 0 | | otu7501 | 0 | 0 | | otu18523 | 0 | 0 | | otu23661 | 0 | 0 | | otu19565 | 0 | 0 | | otu14518 | 0 | 0 | | otu429 | 0 | 0 | | otu10736 | 0 | 0 | | otu8599 | 0 | 0 | | otu13529 | 0 | 0 | | otu10964 | 0 | 0 | | otu9447 | 0 | 0 | | otu19406 | 0 | 0 | | otu4619 | 0 | 0 | | otu9622 | 0 | 0 | | otu4755 | 0 | 0 | | otu11738 | 0 | 0 | | otu14637 | 0 | 0 | | otu20882 | 0 | 0 | | otu22933 | 0 | 0 | | otu4510 | 0 | 0 | | otu12390 | 0 | 0 | | otu19114 | 0 | 0 | | otu20360 | 0 | 0 | | otu18630 | 0 | 0 | | otu12028 | 0 | 0 | | otu16222 | 0 | 0 | | otu22664 | 0 | 0 | | otu19680 | 0 | 0 | | otu6256 | 0 | 0 | | otu9750 | 0 | 0 | | otu17237 | 0 | 0 | | otu999 | 0 | 0 | | otu16557 | 0 | 0 | | otu2848 | 0 | 0 | | otu7358 | 0 | 0 | | otu12516 | 0 | 0 | | otu21514 | 0 | 0 | | otu17178 | 0 | 0 | | otu8571 | 0 | 0 | | otu15974 | 0 | 0 | | otu10396 | 0 | 0 | | otu3150 | 0 | 0 | | otu8556 | 0 | 0 | | otu9463 | 0 | 0 | | otu3070 | 0 | 0 | | otu12637 | 0 | 0 | | otu23851 | 0 | 0 | | otu5457 | 0 | 0 | | otu14646 | 0 | 0 | | otu16205 | 0 | 0 | | otu23573 | 0 | 0 | | otu4533 | 0 | 0 | | otu14043 | 0 | 0 | | otu18221 | 0 | 0 | | otu18685 | 0 | 0 | | otu21783 | 0 | 0 | | otu1537 | 0 | 0 | | otu10040 | 0 | 0 | | otu10551 | 0 | 0 | | otu12650 | 0 | 0 | | otu15686 | 0 | 0 | | otu23801 | 0 | 0 | | otu2940 | 0 | 0 | | otu7931 | 0 | 0 | | otu22485 | 0 | 0 | | otu9673 | 0 | 0 | | otu11130 | 0 | 0 | | otu2770 | 0 | 0 | | otu10340 | 0 | 0 | | otu9212 | 0 | 0 | | otu11253 | 0 | 0 | | otu13562 | 0 | 0 | | otu16526 | 0 | 0 | | otu13980 | 0 | 0 | | otu8769 | 0 | 0 | | otu9539 | 0 | 0 | | otu17981 | 0 | 0 | | otu3643 | 0 | 0 | | otu16958 | 0 | 0 | | otu17223 | 0 | 0 | | otu18006 | 0 | 0 | | otu22471 | 0 | 0 | | otu2950 | 0 | 0 | | otu6798 | 0 | 0 | | otu6911 | 0 | 0 | | otu16137 | 0 | 0 | | otu18239 | 0 | 0 | | otu19514 | 0 | 0 | | otu1236 | 0 | 0 | | otu1684 | 0 | 0 | | otu2944 | 0 | 0 | | otu13051 | 0 | 0 | | otu18592 | 0 | 0 | | otu23748 | 0 | 0 | | otu1099 | 0 | 0 | | otu5481 | 0 | 0 | | otu9753 | 0 | 0 | | otu18187 | 0 | 0 | | otu23725 | 0 | 0 | | otu1502 | 0 | 0 | | otu5541 | 0 | 5.8925565098879e-05 | | otu17166 | 0 | 0 | | otu340 | 0 | 0 | | otu2757 | 0 | 0 | | otu3024 | 0 | 0 | | otu3864 | 0 | 0 | | otu5944 | 0 | 0 | | otu9596 | 0 | 0 | | otu14476 | 0 | 0 | | otu2624 | 0 | 0 | | otu4064 | 0 | 0 | | otu5467 | 0 | 0 | | otu11514 | 0 | 0 | | otu19576 | 0 | 0 | | otu20106 | 0 | 0 | | otu20469 | 0 | 0 | | otu22983 | 0 | 0 | | otu23821 | 0 | 0 | | otu3054 | 0 | 0 | | otu5212 | 0 | 0 | | otu6427 | 0 | 0 | | otu6473 | 0 | 6.73435029701474e-05 | | otu11563 | 0 | 0 | | otu3157 | 0 | 0 | | otu3493 | 0 | 0 | | otu11568 | 0 | 0 | | otu13124 | 0 | 0 | | otu13714 | 0 | 0 | | otu17163 | 0 | 0 | | otu1412 | 0 | 0 | | otu10121 | 0 | 0 | | otu11022 | 0 | 0 | | otu17286 | 0 | 0 | | otu22340 | 0 | 0 | | otu2023 | 0 | 0 | | otu2819 | 0 | 0 | | otu7565 | 0 | 0 | | otu11377 | 0 | 0 | | otu13031 | 0 | 0 | | otu142 | 0 | 0 | | otu7981 | 0 | 0 | | otu11844 | 0 | 0 | | otu13199 | 0 | 0 | | otu19385 | 0 | 0 | | otu21694 | 0 | 0 | | otu3050 | 0 | 0 | | otu5935 | 0 | 0 | | otu9017 | 0 | 0 | | otu11902 | 0 | 0 | | otu13534 | 0 | 0 | | otu21216 | 0 | 0 | | otu5983 | 0 | 0 | | otu6570 | 0 | 0 | | otu7281 | 0 | 0 | | otu10021 | 0 | 0 | | otu11505 | 0 | 0 | | otu12295 | 0 | 0 | | otu13639 | 0 | 0 | | otu13711 | 0 | 0 | | otu13756 | 0 | 0 | | otu17222 | 0 | 0 | | otu18949 | 0 | 0 | | otu22140 | 0 | 0 | | otu1191 | 0 | 0 | | otu3503 | 0 | 0 | | otu5260 | 0 | 9.82092751647983e-05 | | otu9012 | 0 | 7.85674201318386e-05 | | otu9454 | 0 | 0 | | otu16413 | 0 | 0 | | otu17201 | 0 | 0 | | otu17616 | 0 | 0 | | otu17712 | 0 | 0 | | otu19696 | 0 | 0.000102062072615966 | | otu23513 | 0 | 0 | | otu992 | 0 | 0 | | otu1358 | 0 | 0 | | otu1637 | 0 | 0 | | otu2230 | 0 | 0 | | otu6336 | 0 | 0 | | otu7683 | 0 | 0 | | otu7905 | 0 | 0 | | otu8247 | 0 | 0 | | otu8381 | 0 | 0 | | otu8989 | 0 | 0 | | otu11343 | 0 | 0 | | otu14039 | 0 | 0 | | otu21412 | 0 | 0 | | otu3260 | 0 | 0 | | otu3647 | 0 | 0 | | otu3729 | 0 | 6.73435029701474e-05 | | otu4972 | 0 | 0 | | otu11283 | 0 | 0 | | otu16288 | 0 | 0 | | otu22523 | 0 | 0 | | otu993 | 0 | 0 | | otu1154 | 0 | 0 | | otu2195 | 0 | 0 | | otu7406 | 0 | 0 | | otu10877 | 0 | 0 | | otu17109 | 0 | 6.73435029701474e-05 | | otu20098 | 0 | 0 | | otu22185 | 0 | 0 | | otu23421 | 0 | 0 | | otu1781 | 0 | 0 | | otu7296 | 0 | 0 | | otu7614 | 0 | 0 | | otu11646 | 0 | 0 | | otu12936 | 0 | 0 | | otu15486 | 0 | 0 | | otu16356 | 0 | 0 | | otu16984 | 0 | 0 | | otu17363 | 0 | 0 | | otu17473 | 0 | 0 | | otu20961 | 0 | 0 | | otu21213 | 0 | 0 | | otu22159 | 0 | 0 | | otu86 | 0 | 0 | | otu671 | 0 | 0 | | otu2533 | 0 | 0 | | otu4190 | 0 | 0 | | otu6802 | 0 | 0 | | otu7707 | 0 | 0 | | otu9088 | 0 | 0 | | otu9776 | 0 | 0 | | otu12723 | 0 | 0 | | otu15080 | 0 | 0 | | otu15877 | 0 | 0 | | otu19422 | 0 | 0 | | otu22909 | 0 | 0 | | otu23497 | 0 | 0 | | otu947 | 0 | 0 | | otu3400 | 0 | 0 | | otu4830 | 0 | 0 | | otu5095 | 0 | 0 | | otu8364 | 0 | 0 | | otu9269 | 0 | 0 | | otu10373 | 0 | 0 | | otu10996 | 0 | 0 | | otu18328 | 0 | 0 | | otu18565 | 0 | 0 | | otu20982 | 0 | 0 | | otu21083 | 0 | 0 | | otu22390 | 0 | 0 | | otu1454 | 0 | 0 | | otu2296 | 0 | 0 | | otu3095 | 0 | 0 | | otu5717 | 0 | 0 | | otu6940 | 0 | 0 | | otu7381 | 0 | 0 | | otu11497 | 0 | 0 | | otu12820 | 0 | 0 | | otu14175 | 0 | 0 | | otu18010 | 0 | 0 | | otu18095 | 0 | 0 | | otu19746 | 0 | 0 | | otu21179 | 0 | 0 | | otu21463 | 0 | 0 | | otu21864 | 0 | 0 | | otu22082 | 0 | 0 | | otu23730 | 0 | 0 | | otu7193 | 0 | 0 | | otu7328 | 0 | 0 | | otu10831 | 0 | 0 | | otu11538 | 0 | 0 | | otu11661 | 0 | 0 | | otu11845 | 0 | 0 | | otu12656 | 0 | 0 | | otu13127 | 0 | 0 | | otu13181 | 0 | 0 | | otu13543 | 0 | 0 | | otu17273 | 0 | 0 | | otu20969 | 0 | 0 | | otu22309 | 0 | 0 | | otu600 | 0 | 0 | | otu640 | 0 | 0 | | otu896 | 0 | 0 | | otu3014 | 0 | 0 | | otu3940 | 0 | 0 | | otu8615 | 0 | 0 | | otu9488 | 0 | 0 | | otu9871 | 0 | 0 | | otu10130 | 0 | 0 | | otu10277 | 0 | 0 | | otu18197 | 0 | 0.000102062072615966 | | otu18600 | 0 | 0 | | otu18893 | 0 | 0 | | otu23723 | 0 | 0 | | otu504 | 0 | 0 | | otu554 | 0 | 0 | | otu1748 | 0 | 0 | | otu2688 | 0 | 0 | | otu4488 | 0 | 0 | | otu4545 | 0 | 0 | | otu5511 | 0 | 0 | | otu7592 | 0 | 0 | | otu9783 | 0 | 0 | | otu12682 | 0 | 0 | | otu13625 | 0 | 0 | | otu16587 | 0 | 0 | | otu16846 | 0 | 0 | | otu19593 | 0 | 0 | | otu20846 | 0 | 0 | | otu21050 | 0 | 0 | | otu21699 | 0 | 0 | | otu21720 | 0 | 0 | | otu2762 | 0 | 0 | | otu6539 | 0 | 0 | | otu7698 | 0 | 0 | | otu7914 | 0 | 0 | | otu8313 | 0 | 0 | | otu8993 | 0 | 0 | | otu9512 | 0 | 0 | | otu9532 | 0 | 0 | | otu16315 | 0 | 0 | | otu18714 | 0 | 0 | | otu19870 | 0 | 0 | | otu20384 | 0 | 0 | | otu20884 | 0 | 0 | | otu21344 | 0 | 0 | | otu22590 | 0 | 0 | | otu23021 | 0 | 0 | | otu23100 | 0 | 0 | | otu359 | 0 | 0 | | otu528 | 0 | 0 | | otu1578 | 0 | 0 | | otu2076 | 0 | 0 | | otu2896 | 0 | 0 | | otu3020 | 0 | 6.73435029701474e-05 | | otu4004 | 0 | 0 | | otu4913 | 0 | 0 | | otu7403 | 0 | 0 | | otu10502 | 0 | 0 | | otu12221 | 0 | 0 | | otu12991 | 0 | 0 | | otu13156 | 0 | 0 | | otu13634 | 0 | 0 | | otu16111 | 0 | 0 | | otu16956 | 0 | 0 | | otu17173 | 0 | 0 | | otu18987 | 0 | 0 | | otu19690 | 0 | 0 | | otu19799 | 0 | 0 | | otu21145 | 0 | 0 | | otu21562 | 0 | 0 | | otu21578 | 0 | 0 | | otu21683 | 0 | 0 | | otu21961 | 0 | 0 | | otu22291 | 0 | 0 | | otu22472 | 0 | 0 | | otu29 | 0 | 0 | | otu1218 | 0 | 0 | | otu1359 | 0 | 0 | | otu3561 | 0 | 0 | | otu4797 | 0 | 0 | | otu7120 | 0 | 0 | | otu10882 | 0 | 0 | | otu13537 | 0 | 0 | | otu13618 | 0 | 0 | | otu16044 | 0 | 0 | | otu16074 | 0 | 0 | | otu16699 | 0 | 0 | | otu20472 | 0 | 0 | | otu20565 | 0 | 0 | | otu21559 | 0 | 0 | | otu22280 | 0 | 0 | | otu22500 | 0 | 0 | | otu22615 | 0 | 0 | | otu523 | 0 | 0 | | otu1265 | 0 | 0 | | otu4633 | 0 | 0 | | otu5390 | 0 | 0 | | otu5966 | 0 | 0 | | otu6002 | 0 | 0 | | otu6751 | 0 | 0 | | otu7339 | 0 | 0 | | otu8779 | 0 | 0 | | otu8969 | 0 | 0 | | otu9406 | 0 | 0 | | otu9537 | 0 | 0 | | otu10048 | 0 | 0 | | otu10256 | 0 | 0 | | otu10744 | 0 | 0 | | otu11441 | 0 | 0 | | otu12124 | 0 | 0 | | otu12183 | 0 | 0 | | otu13221 | 0 | 0 | | otu13712 | 0 | 0 | | otu14437 | 0 | 0 | | otu15292 | 0 | 0 | | otu17517 | 0 | 0 | | otu18005 | 0 | 0 | | otu18815 | 0 | 0 | | otu19127 | 0 | 0 | | otu19609 | 0 | 0 | | otu19625 | 0 | 0 | | otu20008 | 0 | 0 | | otu20889 | 0 | 0 | | otu22201 | 0 | 0 | | otu22416 | 0 | 0 | | otu23415 | 0 | 0 | | otu23523 | 0 | 0 | | otu1145 | 0 | 0 | | otu2146 | 0 | 0 | | otu3669 | 0 | 0 | | otu5121 | 0 | 0 | | otu6868 | 0 | 0 | | otu7901 | 0 | 0 | | otu8526 | 0 | 0 | | otu8950 | 0 | 0 | | otu9373 | 0 | 0 | | otu11292 | 0 | 0 | | otu12312 | 0 | 0 | | otu13474 | 0 | 0 | | otu14334 | 0 | 0 | | otu15367 | 0 | 0 | | otu15569 | 0 | 0 | | otu16191 | 0 | 0 | | otu20578 | 0 | 0 | | otu20977 | 0 | 0 | | otu21248 | 0 | 0 | | otu22781 | 0 | 0 | | otu22875 | 0 | 0 | | otu66 | 0 | 0 | | otu638 | 0 | 0 | | otu860 | 0 | 0 | | otu3232 | 0 | 0 | | otu3383 | 0 | 0 | | otu3595 | 0 | 0 | | otu3623 | 0 | 0 | | otu4037 | 0 | 0 | | otu4933 | 0 | 0 | | otu5019 | 0 | 0 | | otu5272 | 0 | 0 | | otu5399 | 0 | 0 | | otu5776 | 0 | 0 | | otu6057 | 0 | 0 | | otu7722 | 0 | 0 | | otu7825 | 0 | 0 | | otu8122 | 0 | 0 | | otu9217 | 0 | 0 | | otu9575 | 0 | 0 | | otu9789 | 0 | 0 | | otu13294 | 0 | 0 | | otu14478 | 0 | 0 | | otu14551 | 0 | 0 | | otu15084 | 0 | 0 | | otu16624 | 0 | 0 | | otu19477 | 0 | 0 | | otu20016 | 0 | 0 | | otu21930 | 0 | 0 | | otu22349 | 0 | 0 | | otu22679 | 0 | 0 | | otu23701 | 0 | 0 | | otu1382 | 0 | 0 | | otu1543 | 0 | 0 | | otu2958 | 0 | 0 | | otu3517 | 0 | 0 | | otu3602 | 0 | 0 | | otu3773 | 0 | 0 | | otu4748 | 0 | 0 | | otu4848 | 0 | 0 | | otu8334 | 0 | 0 | | otu8682 | 0 | 0 | | otu9808 | 0 | 0 | | otu11151 | 0 | 0 | | otu12491 | 0 | 0 | | otu12880 | 0 | 0 | | otu12987 | 0 | 0 | | otu13228 | 0 | 0 | | otu13392 | 0 | 0 | | otu13498 | 0 | 0 | | otu15780 | 0 | 0 | | otu16692 | 0 | 0 | | otu16712 | 0 | 0 | | otu17924 | 0 | 0 | | otu18151 | 0 | 0 | | otu18659 | 0 | 0 | | otu19155 | 0 | 0 | | otu19801 | 0 | 0 | | otu21901 | 0 | 0 | | otu22911 | 0 | 0 | | otu1886 | 0 | 0 | | otu2588 | 0 | 0 | | otu3890 | 0 | 0 | | otu3977 | 0 | 0 | | otu4151 | 0 | 0 | | otu4707 | 0 | 0 | | otu5321 | 0 | 0 | | otu5356 | 0 | 0 | | otu6525 | 0 | 0 | | otu6638 | 0 | 0 | | otu6943 | 0 | 0 | | otu6992 | 0 | 0 | | otu7150 | 0 | 0 | | otu7674 | 0 | 0 | | otu7907 | 0 | 0 | | otu8806 | 0 | 0 | | otu9786 | 0 | 0 | | otu10029 | 0 | 0 | | otu10755 | 0 | 0 | | otu11317 | 0 | 0 | | otu11638 | 0 | 0 | | otu12051 | 0 | 0 | | otu14238 | 0 | 0 | | otu15169 | 0 | 0 | | otu16877 | 0 | 0 | | otu17101 | 0 | 0 | | otu17880 | 0 | 0 | | otu19133 | 0 | 0 | | otu21417 | 0 | 0 | | otu21438 | 0 | 0 | | otu21453 | 0 | 0 | | otu23123 | 0 | 0 | | otu23508 | 0 | 0 | | otu23721 | 0 | 0 | | otu209 | 0 | 0 | | otu2124 | 0 | 0 | | otu3659 | 0 | 0 | | otu3718 | 0 | 0 | | otu3999 | 0 | 0 | | otu4561 | 0 | 0 | | otu4810 | 0 | 0 | | otu4898 | 0 | 0 | | otu5462 | 0 | 0 | | otu7883 | 0 | 0 | | otu8711 | 0 | 0 | | otu8816 | 0 | 0 | | otu8959 | 0 | 0 | | otu10418 | 0 | 0 | | otu10531 | 0 | 0 | | otu11136 | 0 | 0 | | otu11209 | 0 | 0 | | otu11644 | 0 | 0 | | otu12242 | 0 | 0 | | otu12635 | 0 | 0 | | otu12652 | 0 | 0 | | otu14420 | 0 | 0 | | otu15268 | 0 | 0 | | otu15529 | 0 | 0 | | otu16139 | 0 | 0 | | otu16397 | 0 | 0 | | otu16495 | 0 | 0 | | otu16787 | 0 | 0 | | otu16987 | 0 | 0 | | otu17465 | 0 | 0 | | otu18383 | 0 | 0 | | otu18429 | 0 | 0 | | otu18465 | 0 | 0 | | otu18489 | 0 | 0 | | otu19378 | 0 | 0 | | otu20616 | 0 | 0 | | otu21377 | 0 | 0 | | otu21835 | 0 | 0 | | otu21848 | 0 | 0 | | otu21924 | 0 | 0 | | otu22550 | 0 | 0 | | otu22730 | 0 | 0 | | otu22774 | 0 | 0 | | otu22806 | 0 | 0 | | otu23217 | 0 | 0 | | otu23464 | 0 | 0 | | otu23755 | 0 | 0 | | otu165 | 0 | 0 | | otu760 | 0 | 0 | | otu1282 | 0 | 0 | | otu1997 | 0 | 0 | | otu2939 | 0 | 0 | | otu3459 | 0 | 0 | | otu6110 | 0 | 0 | | otu6116 | 0 | 0 | | otu6653 | 0 | 0 | | otu6669 | 0 | 0 | | otu7506 | 0 | 0 | | otu7643 | 0 | 0 | | otu8378 | 0 | 0 | | otu8429 | 0 | 0 | | otu8825 | 0 | 0 | | otu9390 | 0 | 0 | | otu10039 | 0 | 0 | | otu10056 | 0 | 0 | | otu10966 | 0 | 0 | | otu11015 | 0 | 0 | | otu11087 | 0 | 0 | | otu12170 | 0 | 0 | | otu12821 | 0 | 0 | | otu12884 | 0 | 0 | | otu13249 | 0 | 0 | | otu13535 | 0 | 0 | | otu15014 | 0 | 0 | | otu16063 | 0 | 0 | | otu16468 | 0 | 0 | | otu16718 | 0 | 0 | | otu16856 | 0 | 0 | | otu17207 | 0 | 0 | | otu17220 | 0 | 0 | | otu17635 | 0 | 0 | | otu17805 | 0 | 0 | | otu17855 | 0 | 0 | | otu18713 | 0 | 0 | | otu18734 | 0 | 0 | | otu19547 | 0 | 0 | | otu19924 | 0 | 0 | | otu20665 | 0 | 0 | | otu21465 | 0 | 0 | | otu22080 | 0 | 0 | | otu22751 | 0 | 0 | | otu22782 | 0 | 0 | | otu23278 | 0 | 0 | | otu23844 | 0 | 0 | | otu615 | 0 | 0 | | otu1118 | 0 | 0 | | otu1296 | 0 | 0 | | otu1366 | 0 | 0 | | otu2577 | 0 | 0 | | otu2992 | 0 | 0 | | otu3033 | 0 | 0 | | otu3852 | 0 | 0 | | otu3872 | 0 | 0 | | otu3910 | 0 | 0 | | otu4729 | 0 | 0 | | otu5394 | 0 | 0 | | otu5593 | 0 | 0 | | otu5707 | 0 | 0 | | otu5716 | 0 | 0 | | otu5943 | 0 | 0 | | otu6255 | 0 | 0 | | otu6373 | 0 | 0 | | otu6417 | 0 | 0 | | otu6531 | 0 | 0 | | otu7070 | 0 | 0 | | otu7125 | 0 | 0 | | otu7220 | 0 | 0 | | otu7245 | 0 | 0 | | otu7871 | 0 | 0 | | otu9104 | 0 | 0 | | otu9187 | 0 | 0 | | otu9487 | 0 | 0 | | otu9794 | 0 | 0 | | otu10901 | 0 | 0 | | otu11551 | 0 | 0 | | otu12056 | 0 | 0 | | otu12942 | 0 | 0 | | otu14147 | 0 | 0 | | otu14428 | 0 | 0 | | otu14479 | 0 | 0 | | otu15365 | 0 | 0 | | otu15567 | 0 | 0 | | otu15604 | 0 | 0 | | otu15765 | 0 | 0 | | otu16450 | 0 | 0 | | otu17329 | 0 | 0 | | otu17488 | 0 | 0 | | otu17790 | 0 | 0 | | otu18742 | 0 | 0 | | otu18749 | 0 | 0 | | otu19198 | 0 | 0 | | otu19331 | 0 | 0 | | otu19743 | 0 | 0 | | otu19762 | 0 | 0 | | otu20518 | 0 | 0 | | otu21125 | 0 | 0 | | otu21688 | 0 | 0 | | otu22110 | 0 | 0 | | otu22129 | 0 | 0 | | otu23024 | 0 | 0 | | otu23036 | 0 | 0 | | otu23190 | 0 | 0 | | otu23585 | 0 | 0 | | otu227 | 0 | 0 | | otu650 | 0 | 0 | | otu1390 | 0 | 0 | | otu1930 | 0 | 0 | | otu1972 | 0 | 0 | | otu2961 | 0 | 0 | | otu3588 | 0 | 0 | | otu4714 | 0 | 0 | | otu4956 | 0 | 0 | | otu5528 | 0 | 0 | | otu5740 | 0 | 0 | | otu5906 | 0 | 0 | | otu6071 | 0 | 0 | | otu6146 | 0 | 0 | | otu6257 | 0 | 0 | | otu6280 | 0 | 0 | | otu6571 | 0 | 0 | | otu6961 | 0 | 0 | | otu7466 | 0 | 0 | | otu7834 | 0 | 0 | | otu8919 | 0 | 0 | | otu9557 | 0 | 0 | | otu10167 | 0 | 0 | | otu10374 | 0 | 0 | | otu10943 | 0 | 0 | | otu11366 | 0 | 0 | | otu12120 | 0 | 0 | | otu12352 | 0 | 0 | | otu12457 | 0 | 0 | | otu13913 | 0 | 0 | | otu13994 | 0 | 0 | | otu14856 | 0 | 0 | | otu15243 | 0 | 0 | | otu15442 | 0 | 0 | | otu17295 | 0 | 0 | | otu17603 | 0 | 0 | | otu18598 | 0 | 0 | | otu18823 | 0 | 0 | | otu18885 | 0 | 0 | | otu19445 | 0 | 0 | | otu19569 | 0 | 0 | | otu19633 | 0 | 0 | | otu20125 | 0 | 0 | | otu20793 | 0 | 0 | | otu21967 | 0 | 0 | | otu22574 | 0 | 0 | | otu23460 | 0 | 0 | | otu46 | 0 | 0 | | otu82 | 0 | 0 | | otu297 | 0 | 0 | | otu728 | 0 | 0 | | otu970 | 0 | 0 | | otu1117 | 0 | 0 | | otu1237 | 0 | 0 | | otu1742 | 0 | 0 | | otu1970 | 0 | 0 | | otu2418 | 0 | 0 | | otu2473 | 0 | 0 | | otu2729 | 0 | 0 | | otu2847 | 0 | 0 | | otu2887 | 0 | 0 | | otu3145 | 0 | 0 | | otu3154 | 0 | 0 | | otu4686 | 0 | 0 | | otu5057 | 0 | 0 | | otu5426 | 0 | 0 | | otu5829 | 0 | 0 | | otu5832 | 0 | 0 | | otu6054 | 0 | 0 | | otu6258 | 0 | 0 | | otu6301 | 0 | 0 | | otu6497 | 0 | 0 | | otu6532 | 0 | 0 | | otu7332 | 0 | 0 | | otu7552 | 0 | 0 | | otu7593 | 0 | 0 | | otu7687 | 0 | 0 | | otu8638 | 0 | 0 | | otu8951 | 0 | 0 | | otu9621 | 0 | 0 | | otu10294 | 0 | 0 | | otu10637 | 0 | 0 | | otu11160 | 0 | 0 | | otu11172 | 0 | 0 | | otu11679 | 0 | 0 | | otu11709 | 0 | 0 | | otu13819 | 0 | 0 | | otu16571 | 0 | 0 | | otu16641 | 0 | 0 | | otu17188 | 0 | 0 | | otu18001 | 0 | 0 | | otu18102 | 0 | 0 | | otu18318 | 0 | 0 | | otu18794 | 0 | 0 | | otu20394 | 0 | 0 | | otu20497 | 0 | 0 | | otu20623 | 0 | 0 | | otu20758 | 0 | 0 | | otu20836 | 0 | 0 | | otu21254 | 0 | 0 | | otu21288 | 0 | 0 | | otu21355 | 0 | 0 | | otu21940 | 0 | 0 | | otu21954 | 0 | 0 | | otu22440 | 0 | 0 | | otu948 | -1.98412698412694e-07 | 0.000222368090353112 | | otu6917 | -3.96825396825394e-07 | 0.00014019229675894 | | otu11730 | -3.96825396825395e-07 | 9.2898210668567e-05 | | otu5722 | -1.11111111111111e-06 | 4.98144706032442e-05 | | otu12467 | -1.38888888888889e-06 | 0.000109519855576202 | | otu23397 | -1.38888888888889e-06 | 5.57288962437191e-05 | | otu7921 | -1.38888888888889e-06 | 5.57288962437191e-05 | | otu2759 | -1.78571428571428e-06 | 0.000104616376193216 | | otu21081 | -1.78571428571428e-06 | 6.324667361495e-05 | | otu15737 | -1.78571428571428e-06 | 6.324667361495e-05 | | otu2084 | -1.78571428571428e-06 | 6.324667361495e-05 | | otu7402 | -1.78571428571428e-06 | 0.000134805331216028 | | otu2691 | -1.78571428571428e-06 | 0.000102218170702516 | | otu17802 | -1.78571428571428e-06 | 5.64692439315782e-06 | | otu93 | -2.06349206349206e-06 | 9.41324712659581e-05 | | otu15988 | -2.61904761904759e-06 | 0.000453700934787238 | | otu19914 | -2.77777777777778e-06 | 0.00014371253313845 | | otu6618 | -3.17460317460317e-06 | 6.02338601936834e-05 | | otu12265 | -3.17460317460317e-06 | 6.02338601936834e-05 | | otu1993 | -3.17460317460317e-06 | 6.02338601936834e-05 | | otu3544 | -3.33333333333334e-06 | 8.67093911783408e-05 | | otu5934 | -3.62193362193362e-06 | 0.000128594994175716 | | otu17063 | -3.96825396825396e-06 | 0.000261348013339672 | | otu3986 | -3.96825396825397e-06 | 0.000177908823583015 | | otu3130 | -4.09451659451659e-06 | 0.000424139340613061 | | otu7728 | -4.28571428571428e-06 | 5.79506397202907e-05 | | otu4227 | -4.64285714285715e-06 | 0.000361506753144498 | | otu11757 | -5e-06 | 0.000106588513035463 | | otu6718 | -5.23809523809524e-06 | 0.000130420972966484 | | otu4520 | -5.55555555555555e-06 | 1.75682092231577e-05 | | otu20521 | -5.71428571428572e-06 | 0.000140931465629699 | | otu8553 | -5.71428571428572e-06 | 8.17051831981989e-05 | | otu21963 | -6.07142857142857e-06 | 0.000226323798852789 | | otu18879 | -6.89033189033189e-06 | 0.000378503875146442 | | otu15858 | -6.98412698412699e-06 | 0.000308325031268818 | | otu13437 | -8.21428571428572e-06 | 7.09917196794003e-05 | | otu9586 | -8.33333333333333e-06 | 0.000151608884212934 | | otu7427 | -8.33333333333333e-06 | 9.97682499781554e-05 | | otu108 | -9.08730158730158e-06 | 0.00018098877712654 | | otu18945 | -9.32539682539682e-06 | 0.000208468818798636 | | otu19882 | -9.32539682539683e-06 | 0.000193599128079106 | | otu16681 | -9.72222222222222e-06 | 8.43685697138191e-05 | | otu16770 | -9.72222222222222e-06 | 3.07443661405259e-05 | | otu21419 | -1e-05 | 0.000122020034784821 | | otu5311 | -1e-05 | 3.16227766016838e-05 | | otu16102 | -1e-05 | 3.16227766016838e-05 | | otu21836 | -1e-05 | 3.16227766016838e-05 | | otu12162 | -1e-05 | 3.16227766016838e-05 | | otu2012 | -1e-05 | 3.16227766016838e-05 | | otu11800 | -1e-05 | 3.16227766016838e-05 | | otu23567 | -1.01190476190476e-05 | 8.35244407317193e-05 | | otu11474 | -1.07142857142857e-05 | 0.000123520470474955 | | otu8219 | -1.08333333333333e-05 | 5.90628980488253e-05 | | otu23757 | -1.09523809523809e-05 | 9.83115199296056e-05 | | otu19946 | -1.11111111111111e-05 | 7.59586419936851e-05 | | otu818 | -1.11111111111111e-05 | 3.51364184463153e-05 | | otu16109 | -1.11111111111111e-05 | 3.51364184463153e-05 | | otu19662 | -1.11111111111111e-05 | 3.51364184463153e-05 | | otu5187 | -1.11111111111111e-05 | 3.51364184463153e-05 | | otu4549 | -1.11111111111111e-05 | 3.51364184463153e-05 | | otu10826 | -1.11111111111111e-05 | 3.51364184463153e-05 | | otu18327 | -1.11111111111111e-05 | 3.51364184463153e-05 | | otu11067 | -1.11111111111111e-05 | 3.51364184463153e-05 | | otu277 | -1.11111111111111e-05 | 3.51364184463153e-05 | | otu11150 | -1.11111111111111e-05 | 3.51364184463153e-05 | | otu3998 | -1.11111111111111e-05 | 3.51364184463153e-05 | | otu9367 | -1.11111111111111e-05 | 3.51364184463153e-05 | | otu14842 | -1.11111111111111e-05 | 3.51364184463153e-05 | | otu20813 | -1.11111111111111e-05 | 3.51364184463153e-05 | | otu848 | -1.11111111111111e-05 | 3.51364184463153e-05 | | otu9159 | -1.11111111111111e-05 | 7.59586419936851e-05 | | otu8007 | -1.11111111111111e-05 | 3.51364184463153e-05 | | otu22266 | -1.11111111111111e-05 | 3.51364184463153e-05 | | otu237 | -1.11111111111111e-05 | 3.51364184463153e-05 | | otu18457 | -1.11111111111111e-05 | 8.19849763747357e-05 | | otu11231 | -1.11111111111111e-05 | 3.51364184463153e-05 | | otu11471 | -1.14285714285714e-05 | 6.91255717603971e-05 | | otu23468 | -1.19047619047619e-05 | 0.000109541061443193 | | otu20173 | -1.19047619047619e-05 | 0.000109541061443193 | | otu15654 | -1.19047619047619e-05 | 0.000109541061443193 | | otu20123 | -1.25e-05 | 0.00019129209165333 | | otu8162 | -1.25e-05 | 3.95284707521047e-05 | | otu22278 | -1.25e-05 | 3.95284707521047e-05 | | otu22254 | -1.25e-05 | 3.95284707521047e-05 | | otu20130 | -1.25e-05 | 3.95284707521047e-05 | | otu2822 | -1.25e-05 | 3.95284707521047e-05 | | otu1648 | -1.25e-05 | 0.000131569048001778 | | otu21984 | -1.25e-05 | 3.95284707521047e-05 | | otu3360 | -1.25e-05 | 3.95284707521047e-05 | | otu22117 | -1.25e-05 | 3.95284707521047e-05 | | otu7545 | -1.25e-05 | 3.95284707521047e-05 | | otu23514 | -1.25e-05 | 3.95284707521047e-05 | | otu6271 | -1.25e-05 | 3.95284707521047e-05 | | otu8782 | -1.25e-05 | 3.95284707521047e-05 | | otu18886 | -1.25e-05 | 3.95284707521047e-05 | | otu18473 | -1.25e-05 | 3.95284707521047e-05 | | otu21160 | -1.25e-05 | 3.95284707521047e-05 | | otu8111 | -1.25e-05 | 3.95284707521047e-05 | | otu20771 | -1.25e-05 | 3.95284707521047e-05 | | otu7806 | -1.25e-05 | 3.95284707521047e-05 | | otu17533 | -1.25e-05 | 3.95284707521047e-05 | | otu11369 | -1.33730158730159e-05 | 0.000190548381565116 | | otu7621 | -1.34920634920635e-05 | 0.000179973916346824 | | otu3027 | -1.37698412698413e-05 | 0.000405460158261316 | | otu2203 | -1.38888888888889e-05 | 9.0010287477887e-05 | | otu20454 | -1.38888888888889e-05 | 9.0010287477887e-05 | | otu9841 | -1.42857142857143e-05 | 4.51753951452626e-05 | | otu8428 | -1.42857142857143e-05 | 4.51753951452626e-05 | | otu16768 | -1.42857142857143e-05 | 4.51753951452626e-05 | | otu9996 | -1.42857142857143e-05 | 4.51753951452626e-05 | | otu12744 | -1.42857142857143e-05 | 4.51753951452626e-05 | | otu20345 | -1.42857142857143e-05 | 4.51753951452626e-05 | | otu293 | -1.42857142857143e-05 | 4.51753951452626e-05 | | otu200 | -1.42857142857143e-05 | 4.51753951452626e-05 | | otu18335 | -1.42857142857143e-05 | 4.51753951452626e-05 | | otu12806 | -1.42857142857143e-05 | 4.51753951452626e-05 | | otu20958 | -1.42857142857143e-05 | 4.51753951452626e-05 | | otu400 | -1.42857142857143e-05 | 4.51753951452626e-05 | | otu22078 | -1.42857142857143e-05 | 4.51753951452626e-05 | | otu22354 | -1.42857142857143e-05 | 4.51753951452626e-05 | | otu16421 | -1.42857142857143e-05 | 4.51753951452626e-05 | | otu17461 | -1.42857142857143e-05 | 4.51753951452626e-05 | | otu7216 | -1.42857142857143e-05 | 4.51753951452626e-05 | | otu2429 | -1.42857142857143e-05 | 4.51753951452626e-05 | | otu22381 | -1.42857142857143e-05 | 4.51753951452626e-05 | | otu15837 | -1.42857142857143e-05 | 4.51753951452626e-05 | | otu22165 | -1.42857142857143e-05 | 4.51753951452626e-05 | | otu22370 | -1.42857142857143e-05 | 4.51753951452626e-05 | | otu3126 | -1.42857142857143e-05 | 4.51753951452626e-05 | | otu2058 | -1.42857142857143e-05 | 4.51753951452626e-05 | | otu7157 | -1.42857142857143e-05 | 4.51753951452626e-05 | | otu8647 | -1.42857142857143e-05 | 4.51753951452626e-05 | | otu16195 | -1.42857142857143e-05 | 4.51753951452626e-05 | | otu6347 | -1.53968253968254e-05 | 6.69852553880732e-05 | | otu9989 | -1.60714285714286e-05 | 0.000102564204768395 | | otu17494 | -1.66666666666667e-05 | 5.2704627669473e-05 | | otu8830 | -1.66666666666667e-05 | 5.2704627669473e-05 | | otu11135 | -1.66666666666667e-05 | 5.2704627669473e-05 | | otu20593 | -1.66666666666667e-05 | 5.2704627669473e-05 | | otu12523 | -1.66666666666667e-05 | 5.2704627669473e-05 | | otu7147 | -1.66666666666667e-05 | 5.2704627669473e-05 | | otu2930 | -1.66666666666667e-05 | 5.2704627669473e-05 | | otu21040 | -1.66666666666667e-05 | 5.2704627669473e-05 | | otu8290 | -1.66666666666667e-05 | 5.2704627669473e-05 | | otu254 | -1.66666666666667e-05 | 5.2704627669473e-05 | | otu1798 | -1.66666666666667e-05 | 5.2704627669473e-05 | | otu21950 | -1.66666666666667e-05 | 5.2704627669473e-05 | | otu10354 | -1.66666666666667e-05 | 5.2704627669473e-05 | | otu13502 | -1.66666666666667e-05 | 5.2704627669473e-05 | | otu9738 | -1.66666666666667e-05 | 5.2704627669473e-05 | | otu10802 | -1.68253968253968e-05 | 6.51296747081452e-05 | | otu10676 | -1.6976911976912e-05 | 0.000223969115032146 | | otu19776 | -1.7449494949495e-05 | 0.000127516026268908 | | otu8195 | -1.76190476190476e-05 | 9.72291866634141e-05 | | otu9468 | -1.8452380952381e-05 | 0.000116859857861631 | | otu1883 | -1.84920634920635e-05 | 0.000111551188207446 | | otu19986 | -1.9047619047619e-05 | 0.000192056932854021 | | otu7240 | -1.9047619047619e-05 | 0.000119206243527102 | | otu2013 | -1.91666666666667e-05 | 7.43334578972549e-05 | | otu9772 | -1.96428571428571e-05 | 0.000169109113729092 | | otu15745 | -2e-05 | 0.000103279555898864 | | otu15004 | -2e-05 | 6.32455532033676e-05 | | otu3212 | -2e-05 | 6.32455532033676e-05 | | otu6558 | -2e-05 | 6.32455532033676e-05 | | otu3575 | -2e-05 | 6.32455532033676e-05 | | otu15917 | -2e-05 | 6.32455532033676e-05 | | otu4297 | -2e-05 | 6.32455532033676e-05 | | otu21405 | -2.03571428571429e-05 | 7.607971860336e-05 | | otu19490 | -2.08333333333333e-05 | 0.000116617052942685 | | otu3296 | -2.0952380952381e-05 | 0.000135362488435254 | | otu15926 | -2.11111111111111e-05 | 5.17889830507619e-05 | | otu20718 | -2.12301587301587e-05 | 8.92246999050725e-05 | | otu1458 | -2.14285714285714e-05 | 0.000178333174391181 | | otu2746 | -2.16666666666667e-05 | 0.000118125796097651 | | otu21617 | -2.17171717171717e-05 | 0.000279437065398589 | | otu21756 | -2.19444444444444e-05 | 0.000155756318858008 | | otu22374 | -2.22222222222222e-05 | 8.46197011762656e-05 | | otu15388 | -2.22222222222222e-05 | 7.02728368926306e-05 | | otu9859 | -2.22222222222222e-05 | 7.02728368926306e-05 | | otu20163 | -2.22222222222222e-05 | 7.02728368926306e-05 | | otu8031 | -2.22222222222222e-05 | 7.02728368926306e-05 | | otu18153 | -2.22222222222222e-05 | 7.02728368926306e-05 | | otu6228 | -2.22222222222222e-05 | 7.02728368926306e-05 | | otu13417 | -2.22222222222222e-05 | 7.02728368926306e-05 | | otu9024 | -2.22222222222222e-05 | 7.02728368926306e-05 | | otu12032 | -2.22222222222222e-05 | 7.02728368926306e-05 | | otu20316 | -2.22222222222222e-05 | 7.02728368926306e-05 | | otu18650 | -2.22222222222222e-05 | 0.000114755062109849 | | otu19532 | -2.23015873015873e-05 | 0.000280357434617427 | | otu11026 | -2.24603174603175e-05 | 0.000161890950235656 | | otu23302 | -2.26190476190476e-05 | 0.000144876599300727 | | otu22161 | -2.28968253968254e-05 | 7.68376326672207e-05 | | otu4852 | -2.31746031746032e-05 | 0.000142282205793613 | | otu14003 | -2.33766233766234e-05 | 5.0780530904295e-05 | | otu11380 | -2.35714285714286e-05 | 0.000148692334808621 | | otu9825 | -2.36111111111111e-05 | 4.98841249890777e-05 | | otu6720 | -2.38095238095238e-05 | 0.000227839374940362 | | otu1003 | -2.45238095238095e-05 | 0.000113044683771952 | | otu5519 | -2.48809523809524e-05 | 0.000184878435473827 | | otu11229 | -2.5e-05 | 0.000147248425341095 | | otu8660 | -2.5e-05 | 7.90569415042095e-05 | | otu14699 | -2.5e-05 | 5.2704627669473e-05 | | otu18270 | -2.5e-05 | 7.90569415042095e-05 | | otu6397 | -2.5e-05 | 7.90569415042095e-05 | | otu13247 | -2.5e-05 | 5.2704627669473e-05 | | otu16983 | -2.5e-05 | 7.90569415042095e-05 | | otu22002 | -2.5e-05 | 7.90569415042095e-05 | | otu9951 | -2.5e-05 | 7.90569415042095e-05 | | otu5206 | -2.5e-05 | 7.90569415042095e-05 | | otu319 | -2.5e-05 | 7.90569415042095e-05 | | otu5250 | -2.53968253968254e-05 | 5.4061544018814e-05 | | otu11926 | -2.53968253968254e-05 | 8.03118135915779e-05 | | otu18510 | -2.56746031746032e-05 | 0.000348179006790035 | | otu22573 | -2.57575757575758e-05 | 0.000226092384906675 | | otu4996 | -2.63492063492063e-05 | 0.000153894962464725 | | otu11326 | -2.63492063492063e-05 | 0.000195409654911297 | | otu11789 | -2.67857142857143e-05 | 0.00011080065755349 | | otu9741 | -2.67857142857143e-05 | 5.66258856858347e-05 | | otu17916 | -2.67857142857143e-05 | 9.68469433481411e-05 | | otu16765 | -2.67857142857143e-05 | 5.66258856858347e-05 | | otu1283 | -2.67857142857143e-05 | 5.66258856858347e-05 | | otu570 | -2.67857142857143e-05 | 8.47038658973673e-05 | | otu20206 | -2.71825396825397e-05 | 0.000195127767575536 | | otu18182 | -2.71825396825397e-05 | 0.000181402817611357 | | otu13993 | -2.73809523809524e-05 | 7.85774408474118e-05 | | otu3246 | -2.77777777777778e-05 | 6.00068583185913e-05 | | otu7562 | -2.79761904761905e-05 | 0.000148638790945861 | | otu399 | -2.81746031746032e-05 | 9.84974673318999e-05 | | otu5855 | -2.84126984126984e-05 | 0.000185808776215097 | | otu16975 | -2.85714285714286e-05 | 0.000104435384478098 | | otu2938 | -2.85714285714286e-05 | 9.03507902905251e-05 | | otu22985 | -2.85714285714286e-05 | 9.03507902905251e-05 | | otu20365 | -2.85714285714286e-05 | 9.03507902905251e-05 | | otu7198 | -2.85714285714286e-05 | 9.03507902905251e-05 | | otu13243 | -2.85714285714286e-05 | 6.02338601936834e-05 | | otu18282 | -2.85714285714286e-05 | 9.03507902905251e-05 | | otu17594 | -2.87301587301587e-05 | 0.000174617123059976 | | otu11375 | -2.88888888888889e-05 | 0.000128047219942408 | | otu13852 | -2.88888888888889e-05 | 0.00013846973789517 | | otu6760 | -2.91666666666667e-05 | 0.000412384616915342 | | otu22074 | -2.91666666666667e-05 | 6.22680882540553e-05 | | otu6819 | -2.98412698412698e-05 | 8.27490222822634e-05 | | otu5195 | -3e-05 | 0.000108367357746536 | | otu7434 | -3e-05 | 9.48683298050514e-05 | | otu6477 | -3.02777777777778e-05 | 7.92892163877973e-05 | | otu7488 | -3.03210678210678e-05 | 0.000128794796352363 | | otu21847 | -3.03571428571429e-05 | 8.76638407759541e-05 | | otu7782 | -3.08333333333333e-05 | 0.000186290451642358 | | otu4407 | -3.0952380952381e-05 | 9.78800228147356e-05 | | otu22379 | -3.15079365079365e-05 | 0.000184395159112043 | | otu13744 | -3.1547619047619e-05 | 0.000123464683074725 | | otu3901 | -3.22222222222222e-05 | 5.19707631219587e-05 | | otu19270 | -3.24675324675325e-05 | 6.90608490629602e-05 | | otu18560 | -3.25e-05 | 7.07597657178459e-05 | | otu18315 | -3.31746031746032e-05 | 0.000305526075675402 | | otu12247 | -3.33333333333333e-05 | 7.49942841900614e-05 | | otu10251 | -3.33333333333333e-05 | 0.000120761472884912 | | otu3671 | -3.33333333333333e-05 | 7.02728368926307e-05 | | otu16353 | -3.33333333333333e-05 | 0.000105409255338946 | | otu20650 | -3.33333333333333e-05 | 0.000105409255338946 | | otu12503 | -3.33333333333333e-05 | 0.000105409255338946 | | otu16901 | -3.33333333333333e-05 | 0.000105409255338946 | | otu19457 | -3.33333333333333e-05 | 0.000105409255338946 | | otu21651 | -3.33333333333333e-05 | 0.000105409255338946 | | otu3501 | -3.3531746031746e-05 | 0.000311156619538981 | | otu12645 | -3.36904761904762e-05 | 0.000188038342811638 | | otu23778 | -3.36904761904762e-05 | 0.000139081555708591 | | otu12590 | -3.41269841269841e-05 | 0.000238918330376522 | | otu6232 | -3.42857142857143e-05 | 7.35247964404525e-05 | | otu18727 | -3.44444444444444e-05 | 0.000214843548525283 | | otu22092 | -3.47222222222222e-05 | 7.67038959526893e-05 | | otu18009 | -3.47222222222222e-05 | 7.67038959526893e-05 | | otu8722 | -3.5e-05 | 8.18195847242238e-05 | | otu8075 | -3.59126984126984e-05 | 0.000254924844326449 | | otu11594 | -3.61111111111111e-05 | 8.28690771676461e-05 | | otu12323 | -3.63023088023088e-05 | 0.000420584446262535 | | otu4389 | -3.63095238095238e-05 | 0.000161348689089498 | | otu22753 | -3.65079365079365e-05 | 7.92061901579343e-05 | | otu20247 | -3.66269841269841e-05 | 0.00015196358208172 | | otu4916 | -3.66666666666667e-05 | 0.000115950180872841 | | otu18116 | -3.69444444444444e-05 | 0.000158897888672768 | | otu819 | -3.70238095238095e-05 | 0.000213663448350432 | | otu8864 | -3.75e-05 | 0.000118585412256314 | | otu16290 | -3.75e-05 | 0.000115286144274706 | | otu712 | -3.75e-05 | 8.43685697138191e-05 | | otu2245 | -3.75e-05 | 8.43685697138191e-05 | | otu15819 | -3.75e-05 | 0.000118585412256314 | | otu5241 | -3.76190476190476e-05 | 8.21250268202959e-05 | | otu11989 | -3.78968253968254e-05 | 0.000140689543190168 | | otu1966 | -3.83333333333333e-05 | 0.000136490401236239 | | otu12442 | -3.86904761904762e-05 | 0.000153516816917045 | | otu20985 | -3.92857142857143e-05 | 6.34424409260677e-05 | | otu651 | -3.96825396825397e-05 | 0.000119077009893406 | | otu10132 | -3.98484848484848e-05 | 0.000131557983348893 | | otu7942 | -3.98809523809524e-05 | 0.000146426988181076 | | otu3961 | -4e-05 | 0.000126491106406735 | | otu21113 | -4e-05 | 8.43274042711568e-05 | | otu19674 | -4.08333333333333e-05 | 0.000102473272637007 | | otu19030 | -4.10714285714286e-05 | 9.450986463048e-05 | | otu16274 | -4.16666666666667e-05 | 9.0010287477887e-05 | | otu882 | -4.16666666666667e-05 | 9.0010287477887e-05 | | otu1729 | -4.20634920634921e-05 | 9.10337410925243e-05 | | otu4058 | -4.20634920634921e-05 | 0.000198423280141109 | | otu16215 | -4.28571428571429e-05 | 0.000135526185435788 | | otu7114 | -4.34126984126984e-05 | 0.000330322348295553 | | otu6861 | -4.34523809523809e-05 | 0.000179147244265146 | | otu19990 | -4.39285714285714e-05 | 0.000263850334004401 | | otu19729 | -4.44444444444444e-05 | 0.000115387594273304 | | otu15270 | -4.44444444444444e-05 | 0.000107343531453255 | | otu15961 | -4.44444444444444e-05 | 9.36971158568409e-05 | | otu1416 | -4.44444444444444e-05 | 0.000107343531453255 | | otu235 | -4.46428571428571e-05 | 0.000247678341751024 | | otu18497 | -4.72222222222222e-05 | 9.97682499781554e-05 | | otu22202 | -4.72222222222222e-05 | 0.000134848642479636 | | otu18066 | -4.72222222222222e-05 | 0.000220199124251182 | | otu7641 | -4.7536075036075e-05 | 0.000607850703684516 | | otu4259 | -4.76190476190476e-05 | 0.000121808089240437 | | otu9479 | -4.76190476190476e-05 | 0.000109971479845643 | | otu5226 | -4.76190476190476e-05 | 0.000125208037737558 | | otu20567 | -4.86111111111111e-05 | 0.00034145924207754 | | otu18934 | -4.87301587301587e-05 | 0.000219306169778993 | | otu19761 | -4.91666666666667e-05 | 8.11234769873587e-05 | | otu2265 | -4.96031746031746e-05 | 0.000267954812684996 | | otu21239 | -4.98015873015873e-05 | 0.000235128954092196 | | otu9140 | -5e-05 | 0.000112491426285092 | | otu12505 | -5e-05 | 8.46602181530553e-05 | | otu4558 | -5e-05 | 0.000105409255338946 | | otu22555 | -5e-05 | 0.000120761472884912 | | otu14278 | -5.0952380952381e-05 | 0.000119671381737464 | | otu17703 | -5.17063492063492e-05 | 0.000481188281053869 | | otu22524 | -5.22222222222222e-05 | 0.000111609991145313 | | otu603 | -5.25e-05 | 0.000128262967722133 | | otu21204 | -5.27380952380952e-05 | 0.000146956237283393 | | otu980 | -5.27777777777778e-05 | 9.1146108906109e-05 | | otu13092 | -5.31746031746032e-05 | 8.77453847249095e-05 | | otu15466 | -5.35714285714286e-05 | 0.000131492393061753 | | otu19440 | -5.3968253968254e-05 | 9.73314902651168e-05 | | otu5450 | -5.41666666666667e-05 | 0.000106068037925711 | | otu21721 | -5.53571428571429e-05 | 9.83314596846748e-05 | | otu3453 | -5.57106782106782e-05 | 0.000686976899252867 | | otu7091 | -5.60786435786436e-05 | 0.000241236072050721 | | otu22178 | -5.63492063492063e-05 | 0.000100002099583233 | | otu7504 | -5.63492063492064e-05 | 0.000239516992597036 | | otu15230 | -5.63888888888889e-05 | 0.0001782692377412 | | otu9811 | -5.69444444444444e-05 | 0.000193167100735566 | | otu16028 | -5.69444444444444e-05 | 7.53506890310139e-05 | | otu476 | -5.71428571428571e-05 | 9.98865569685858e-05 | | otu10912 | -5.71428571428571e-05 | 9.98865569685858e-05 | | otu2587 | -5.71428571428571e-05 | 9.98865569685858e-05 | | otu20322 | -5.75e-05 | 0.000107399824125658 | | otu9933 | -5.76190476190476e-05 | 0.000109706541834294 | | otu6498 | -5.81349206349206e-05 | 9.61343138032274e-05 | | otu10450 | -5.83333333333333e-05 | 0.000124536176508111 | | otu17576 | -5.86111111111111e-05 | 8.57114380773299e-05 | | otu1477 | -5.9047619047619e-05 | 0.000192766651969066 | | otu14674 | -5.96825396825397e-05 | 0.000222101504122126 | | otu16993 | -5.96825396825397e-05 | 0.000134102092176924 | | otu20096 | -5.98412698412698e-05 | 0.00016607023540436 | | otu15308 | -6.01190476190476e-05 | 0.000111056179732104 | | otu17379 | -6.01587301587302e-05 | 0.00022891412251106 | | otu2991 | -6.05194805194805e-05 | 0.000165750786464448 | | otu12980 | -6.06746031746032e-05 | 0.0001258114461734 | | otu246 | -6.11111111111111e-05 | 0.000138269594364139 | | otu17314 | -6.22222222222222e-05 | 0.000127355717335333 | | otu6181 | -6.25e-05 | 0.000197642353760524 | | otu21998 | -6.31493506493506e-05 | 0.000267531121282285 | | otu16637 | -6.31709956709957e-05 | 0.000226114199725041 | | otu13659 | -6.34523809523809e-05 | 0.000317150722604884 | | otu5682 | -6.36904761904762e-05 | 0.00019146671189444 | | otu1974 | -6.41269841269841e-05 | 0.000198539218920513 | | otu16196 | -6.42857142857143e-05 | 0.000136567908568571 | | otu12024 | -6.5e-05 | 0.000128668393770792 | | otu7998 | -6.53571428571429e-05 | 8.90898400159991e-05 | | otu9807 | -6.55952380952381e-05 | 0.000296035413422773 | | otu14325 | -6.60714285714286e-05 | 0.000150677720450693 | | otu8272 | -6.60714285714286e-05 | 0.000208936202546839 | | otu2607 | -6.62698412698413e-05 | 0.000148136927957576 | | otu3721 | -6.66666666666667e-05 | 0.000109713431434064 | | otu9779 | -6.66666666666667e-05 | 0.000140545673785261 | | otu5527 | -6.72222222222222e-05 | 0.000140542088390708 | | otu22722 | -6.74206349206349e-05 | 0.000128966619172925 | | otu10440 | -6.76190476190476e-05 | 0.000169682465440167 | | otu21328 | -6.78571428571429e-05 | 0.000170408672450256 | | otu5403 | -7.00793650793651e-05 | 0.000410980738999346 | | otu13161 | -7.02380952380952e-05 | 0.000140685656762317 | | otu20337 | -7.03968253968254e-05 | 9.75800025153951e-05 | | otu23530 | -7.08333333333333e-05 | 0.000149652374967233 | | otu10414 | -7.08333333333333e-05 | 7.61992595796956e-05 | | otu21716 | -7.1547619047619e-05 | 0.000277341181834479 | | otu10471 | -7.17857142857143e-05 | 0.000170684793376113 | | otu23210 | -7.17857142857143e-05 | 0.000128884649336013 | | otu17919 | -7.19877344877345e-05 | 0.000118846468949955 | | otu3217 | -7.20707070707071e-05 | 0.000196701060011096 | | otu5339 | -7.25793650793651e-05 | 0.000166342850163647 | | otu8652 | -7.42857142857143e-05 | 0.000109194351500418 | | otu20207 | -7.42857142857143e-05 | 0.000135492718047838 | | otu3337 | -7.48809523809524e-05 | 0.000415644334533731 | | otu4709 | -7.51190476190476e-05 | 0.00020789022752602 | | otu22360 | -7.55555555555556e-05 | 0.000160657086953789 | | otu10809 | -7.61904761904762e-05 | 0.000131276654781812 | | otu10855 | -7.77777777777778e-05 | 0.000139073950781619 | | otu13253 | -7.85714285714286e-05 | 0.00017317234805684 | | otu14554 | -7.90079365079365e-05 | 0.000161758853098702 | | otu6636 | -7.91666666666667e-05 | 0.000140888385032397 | | otu12612 | -7.91666666666667e-05 | 0.000250346981429997 | | otu16610 | -7.98015873015873e-05 | 0.00019779882549558 | | otu3007 | -7.99603174603175e-05 | 0.000110795920103804 | | otu7218 | -8.09523809523809e-05 | 0.000157392956519537 | | otu16504 | -8.17460317460317e-05 | 0.000206689788122973 | | otu19196 | -8.2063492063492e-05 | 0.000267306331569455 | | otu2990 | -8.23412698412698e-05 | 0.000239526214978986 | | otu21822 | -8.33333333333333e-05 | 0.000230739551747724 | | otu1190 | -8.39285714285714e-05 | 0.000137305314657178 | | otu5833 | -8.45093795093795e-05 | 0.000404821041708815 | | otu16237 | -8.52380952380952e-05 | 0.00021138557763921 | | otu9009 | -8.5515873015873e-05 | 0.000356491285412594 | | otu15485 | -8.56746031746032e-05 | 0.00030452388104936 | | otu3032 | -8.69047619047619e-05 | 0.000201841623240391 | | otu19686 | -8.72222222222222e-05 | 0.000215038995410891 | | otu14801 | -8.80555555555555e-05 | 0.000289131621404914 | | otu7694 | -8.93650793650794e-05 | 0.000346508932256089 | | otu17980 | -8.98809523809524e-05 | 0.000155764990791631 | | otu20597 | -9.05555555555556e-05 | 0.000303819238113165 | | otu302 | -9.10714285714286e-05 | 0.000180254778839188 | | otu18532 | -9.13492063492064e-05 | 0.000109627567599247 | | otu5020 | -9.24603174603175e-05 | 0.000227949624536016 | | otu6829 | -9.27380952380952e-05 | 0.000288221172571461 | | otu13061 | -9.32539682539683e-05 | 0.000287225939352342 | | otu7155 | -9.32575757575757e-05 | 0.000270539850159514 | | otu17405 | -9.3452380952381e-05 | 0.000145344581051784 | | otu7943 | -9.44047619047619e-05 | 0.000138038268215968 | | otu11421 | -9.44444444444444e-05 | 0.000281137094392792 | | otu16462 | -9.47619047619048e-05 | 0.000150108067414121 | | otu20656 | -9.5515873015873e-05 | 0.0001694192008341 | | otu21009 | -9.70634920634921e-05 | 0.000349624579197288 | | otu11872 | -9.8015873015873e-05 | 0.000250802162023772 | | otu18513 | -9.86111111111111e-05 | 0.000244782718052434 | | otu19322 | -9.98015873015873e-05 | 0.000157463114281144 | | otu9588 | -1e-04 | 0.000140545673785261 | | otu22721 | -0.000101865079365079 | 0.000122251143249048 | | otu11212 | -0.00010297619047619 | 0.000196419552462501 | | otu9636 | -0.000103373015873016 | 0.000256368947891434 | | otu9678 | -0.00010452380952381 | 0.000151437431107499 | | otu42 | -0.00010468253968254 | 0.000212627475733109 | | otu12736 | -0.000104880952380952 | 0.000199236389908711 | | otu1859 | -0.00010531746031746 | 0.000313948621620546 | | otu19715 | -0.000106150793650794 | 0.000202552167607376 | | otu21703 | -0.000107142857142857 | 0.000194160790836906 | | otu23195 | -0.000107142857142857 | 0.000382841299127276 | | otu8653 | -0.000107738095238095 | 0.000101731745647867 | | otu634 | -0.000108174603174603 | 0.000242003216250002 | | otu20776 | -0.000110793650793651 | 0.000147762489334703 | | otu21466 | -0.000111443001443001 | 0.000231997667134741 | | otu22012 | -0.000111626984126984 | 0.000184426739558596 | | otu18750 | -0.000112698412698413 | 0.000108807050657743 | | otu12486 | -0.00011290404040404 | 0.000335041409014556 | | otu2019 | -0.000113452380952381 | 0.000165888674020237 | | otu18493 | -0.000116587301587302 | 0.000278364887229041 | | otu15854 | -0.000116829004329004 | 0.000174670327220431 | | otu1647 | -0.000119285714285714 | 0.00017657775968513 | | otu1506 | -0.000120238095238095 | 0.000248218083352568 | | otu19695 | -0.000120277777777778 | 0.000336158164136278 | | otu1922 | -0.000121230158730159 | 0.00013575595371973 | | otu12241 | -0.000121269841269841 | 0.000219604463728195 | | otu6630 | -0.00012202380952381 | 0.000279093460754549 | | otu23194 | -0.000122301587301587 | 0.000222567900074591 | | otu1482 | -0.000124563492063492 | 0.000171288323852798 | | otu3834 | -0.00012734126984127 | 0.000339242339193439 | | otu14987 | -0.00012797619047619 | 0.000214602389462732 | | otu21511 | -0.000128968253968254 | 0.000247990298505057 | | otu22218 | -0.00013031746031746 | 0.000224014503303769 | | otu930 | -0.000130912698412698 | 0.000287544199379295 | | otu20388 | -0.000131785714285714 | 0.000347104437064462 | | otu16106 | -0.000132770562770563 | 0.000191019518659794 | | otu7391 | -0.000133055555555556 | 0.000267746175328971 | | otu7057 | -0.000136428571428571 | 0.000289449291227786 | | otu15298 | -0.000136865079365079 | 0.000302048777284058 | | otu22491 | -0.000137261904761905 | 0.000184430382560981 | | otu14226 | -0.000138015873015873 | 0.000187025324614757 | | otu15913 | -0.000138221500721501 | 0.000268788797845102 | | otu9442 | -0.000138690476190476 | 0.000306276849132344 | | otu8908 | -0.000138888888888889 | 0.000224238537746525 | | otu23240 | -0.000138928571428571 | 0.000309767938929958 | | otu16721 | -0.000139642857142857 | 0.000252059450059551 | | otu23281 | -0.000140555555555556 | 0.000577432808570918 | | otu1293 | -0.000141666666666667 | 0.000254739033735786 | | otu14777 | -0.000141785714285714 | 0.000235363670287556 | | otu20205 | -0.000146230158730159 | 0.000187289746621518 | | otu13072 | -0.000146349206349206 | 0.000215948282863643 | | otu19054 | -0.000148174603174603 | 0.000280981786342673 | | otu13203 | -0.000152539682539683 | 0.000293603015799554 | | otu20014 | -0.000153019480519481 | 0.000348264068975585 | | otu439 | -0.00015515873015873 | 0.000238545136376677 | | otu4942 | -0.000160873015873016 | 0.000362435078976833 | | otu17194 | -0.000162088744588745 | 0.000311762726332965 | | otu15254 | -0.000162698412698413 | 0.00024412378186665 | | otu5583 | -0.000165555555555556 | 0.000152269511167673 | | otu5385 | -0.000165670995670996 | 0.000352049265661434 | | otu4146 | -0.000165714285714286 | 0.000199590788217404 | | otu12833 | -0.000166785714285714 | 0.000263332119413182 | | otu11161 | -0.000169365079365079 | 0.000241847548748121 | | otu13826 | -0.000171111111111111 | 0.000309410948084677 | | otu23133 | -0.000171518759018759 | 0.000272857518555043 | | otu18912 | -0.000171706349206349 | 0.00041257124999589 | | otu3301 | -0.000176587301587302 | 0.00022806657207946 | | otu3359 | -0.000189126984126984 | 0.000236808043205307 | | otu23204 | -0.000191619769119769 | 0.000385544483349161 | | otu332 | -0.000197619047619048 | 0.000211261566044404 | | otu5694 | -0.000205674603174603 | 0.000261633393831597 | | otu13888 | -0.000206746031746032 | 0.000232075579271054 | | otu23249 | -0.000213174603174603 | 0.00025300383187509 | | otu7327 | -0.000220396825396825 | 0.000379583132303565 | | otu10949 | -0.000224166666666667 | 0.000286004864607012 | | otu18323 | -0.000227857142857143 | 0.000236121211231516 | | otu23354 | -0.000229603174603175 | 0.000399962651527549 | | otu22215 | -0.000231511544011544 | 0.000370650227708134 | | otu11839 | -0.000250992063492063 | 0.000468226101837244 | | otu16138 | -0.000256150793650794 | 0.000372520039418289 | | otu365 | -0.000269642857142857 | 0.000252426721111471 | | otu14366 | -0.000293611111111111 | 0.00050343367785838 | | otu2777 | -0.000298928571428571 | 0.000427489532336818 | | otu9186 | -0.000306468253968254 | 0.000238011599801211 | | otu17052 | -0.000310396825396825 | 0.000276247852804829 | 注：表中第二列记录了每个OTU对模型预报准确率的贡献大小，即，如果移除该OTU，模型预报错误率将增加的幅度大小，以此确定它的重要度数值，并已根据其对于模型的重要程度进行排序。此数值越高，该OTU对模型预报准确率的贡献越大。第三列对应每个OTU重要度数值的标准偏差。我们主要根据第二列“Mean decrease in accuracy”中的数值筛选群落中与某种分组方式相关度最大的一组微生物类群。 结果目录: C05\_random\_forests RDA冗余分析 RDA分析是一种典型的约束排序方法，可以理解为“有约束条件”的PCA分析，通过多元线性回归（Multiple linear regression，MLR），将菌群结构数据与某种给定的影响因素互相拟合，并通过置换检验来判断该因素对于菌群结构的影响是否显著。 方法步骤 使用R软件对属水平的相对丰度矩阵进行RDA分析，通过1000次置换检验确定统计显著性，并生成包含“样本—分类单元—影响因素”三种元素排序图。 结果说明 **RDA约束排序图** 注：每个点代表一个样本，两点之间的距离越接近，说明两个样本的群落结构相似度越高。箭头分别代表不同的影响因素，影响因素之间的夹角为锐角时表示两个因素正相关，钝角时为负相关，射线越长，表明该因素的作用越大。样本在箭头上投影点的位置近似代表该因素在对应样本中的数值大小。 结果目录: D01\_rda 关联网络分析 优势物种互作Spearman关联网络分析 最近基于微生物成员之间相互关系的网络推断(Faust and Raes, 2012)分析也逐渐开始流行。这类分析的根本目的是考察不同群落成员之间的相互作用，通过关联分析的方法，找寻群落成员在不同生境下共同出现（Co-occurrence）或彼此排斥（Co-exclusion）的相互作用模式，从而推断不同微生物类群之间可能的相互“协作”或“竞争”关系。 根据OTU或各分类单元在不同样本中的丰度分布，可以寻找彼此之间呈现正相关或负相关的微生物类群，进而构建优势微生物类群的关联网络，探索它们彼此相关的生态学意义。 方法步骤 使用Mothur软件，计算丰度位于前50位的优势属之间的Spearman等级相关系数，对其中rho > 0.6且P值< 0.01的相关优势属构建关联网络，并导入Cytoscape（http://www.cytoscape.org/）(Shannon et al., 2003)软件进行可视化。 结果说明 **优势属的关联网络图** 注：节点代表各优势属，以不同的颜色标识，节点之间的连接表明两个属之间存在相关性，红线表明正相关，绿线表明负相关。通过某节点的连接越多，表明该属与菌群中其它成员的关联越多。 结果目录: C06\_network\relation\_network\_graph 菌群代谢功能预测 以上分析的关注重点是菌群的组成和结构。对于微生物生态学研究，我们最关注的无疑是菌群所具备的代谢功能。随着数据分析技术的发展，我们现在已能根据已知的微生物基因组数据，对菌群组成的测序数据（典型的如16S rRNA基因的测序结果）进行菌群代谢功能的预测，从而把物种的“身份”和它们的“功能”对应起来。根据菌群代谢功能预测结果，我们一方面能一窥菌群功能谱的概貌，发挥菌群多样性组成谱测序性价比高的优势；另一方面也能帮助指导后续宏基因组De novo鸟枪法测序的实验设计，更合理地筛选用于后续研究的样本。 PICRUSt功能预测分析 PICRUSt（Phylogenetic Investigation of Communities by Reconstruction of Unobserved States）是由美国哈佛大学的Curtis Huttenhower课题组开发的菌群代谢功能预测工具，通过将现有的16S rRNA基因测序数据与代谢功能已知的微生物参考基因组数据库相对比，从而实现对细菌和古菌代谢功能的预测；预测过程中还考虑了不同物种16S rRNA基因拷贝数的差异，并对原始数据中的物种丰度数据进行校正，使预测结果更准确可靠(Langille et al., 2013)。 方法步骤 PICRUSt（http://huttenhower.sph.harvard.edu/galaxy/tool\_runner?tool\_id=PICRUSt\_normalize）分析的总体思路如下： 1. 先根据已测微生物基因组的16S rRNA基因全长序列，推断它们的共同祖先的基因功能谱； 2. 对Greengenes 16S rRNA基因全长序列数据库中其它未测物种的基因功能谱进行推断，构建古菌和细菌域全谱系的基因功能预测谱； 3. 将测序得到的16S rRNA基因序列数据与Greengenes数据库比对，寻找每一条测序序列的“参考序列最近邻居”，并归为参考OTU； 4. 根据“参考序列最近邻居”的rRNA基因拷贝数，对获得的OTU丰度矩阵进行校正； 5. 最后，将菌群组成数据“映射”到已知的基因功能谱数据库中，实现对菌群代谢功能的预测。 结果说明 PICRUSt能将16S rRNA基因序列在3种功能谱数据库中进行预测，即KEGG、COG和Rfam。 其中，KEGG数据库的核心为生物代谢通路分析数据库（KEGG PATHWAY Database，http://www.genome.jp/kegg/pathway.html），其中将代谢通路归为6大类，包括代谢（Metabolism）、遗传信息处理（Genetic Information Processing）、环境信息处理（Environmental Information Processing）、细胞进程（Cellular Processes）、生物体系统（Organismal Systems）和人类疾病（Human Diseases），每一类代谢通路又被进一步划分为多个等级。目前，第二等级一共包括45种代谢通路子功能，第三等级即对应代谢通路图，而第四等级则对应代谢通路上各个KO（KEGG orthologous groups，KEGG直系同源基因簇）的具体注释信息。 COG（Clusters of Orthologous Groups，https://www.ncbi.nlm.nih.gov/COG/）数据库是由NCBI维护的直系同源基因数据库，是指不同个体中由于物种形成（Speciation）的进化历程而产生的的同源基因，这些基因来源于共同祖先；因此，在进化历程中，直系同源基因通常都保留了相同或相似的功能特性。 Rfam数据库（http://rfam.xfam.org）为RNA家族数据库，包含了生物体中各类RNA元件的注释信息，如非编码RNA（Non-coding RNA，ncRNA）等。 根据PICRUSt的预测结果，可以获得每样本对应于各功能谱数据库的注释信息，以及预测得到的功能类群的丰度矩阵（predicted\_metagenomes\_kegg.txt、predicted\_metagenomes\_cog.txt和predicted\_metagenomes\_rfam.txt）。 功能类群分布统计 方法步骤 根据预测得到的各功能类群在各样本中的丰度分布，绘制柱形图或小提琴图进行展示。 结果说明 **PICRUSt预测的KEGG第二等级分布图** 注：图中横坐标为KEGG第二等级功能类群，纵坐标为各功能类群在各样本（组）内的相对丰度。样本无分组情况下，将以柱形图的形式展示；样本有分组的情况下，将以小提琴图结合箱线图的形式展示：其中，小提琴图可以直观地显示数据的分布特征，“小提琴”的“胖瘦”反映了样本数据分布的密度高低（宽度越宽，表明该丰度下对应的样本越多）；箱线图边框代表上下四分位数间距（Interquartile range，IQR），横线代表中位值，上下触须分别代表上下四分位以外的1.5倍IQR范围，符号“•”表示超过范围的极端值。 共有功能类群的Venn图分析 方法步骤 根据预测得到的各功能类群在各样本中的丰度分布，使用R软件计算各样本（组）共有功能类群的数量，并通过Venn图（https://en.wikipedia.org/wiki/Venn\_diagram）直观地呈现各样本（组）所共有和独有的功能类群所占的比例。 结果说明 venn.illness-normal.png **共有功能类群的Venn图** 注：每个椭圆代表一个（组）样本，椭圆间的重叠区域表明样本（组）间的共有功能类群，每个区块的数字表明该区块所包含的样本（组）的共有或独有功能类群的数量。 结合聚类分析的功能类群热图分析 将预测得到的功能谱数据，根据功能类群的丰度分布或样本间的相似程度加以聚类，根据聚类结果对功能类群和样本分别排序，并通过热图加以呈现。通过聚类，可以将高丰度和低丰度的功能类群加以区分，并以颜色梯度反映样本之间的功能谱相似度。 方法步骤 使用R软件，对丰度前50位的功能类群进行聚类分析并绘制热图。 结果说明 **结合聚类分析的KEGG直系同源基因簇（KO）丰度热图** 注：样本先按照彼此之间功能类群丰度分布的相似度进行聚类，根据聚类结果横向依次排列。同理，功能类群也按照彼此在不同样本中分布的相似度进行聚类，根据聚类结果纵向依次排列。图中，红色代表在对应样本中丰度较高的功能类群，绿色代表丰度较低的功能类群。 结果目录: C07\_picrust 参考文献 [1] Anderson, M.J., and Willis, T.J. (2003). Canonical analysis of principal coordinates: a useful method of constrained ordination for ecology. Ecology 84, 511-525. [2] Asnicar, F., Weingart, G., Tickle, T.L., Huttenhower, C., and Segata, N. (2015). Compact graphical representation of phylogenetic data and metadata with GraPhlAn. Peerj 3. [3] Blaxter, M., Mann, J., Chapman, T., Thomas, F., Whitton, C., Floyd, R., and Abebe, E. (2005). Defining operational taxonomic units using DNA barcode data. Philos Trans R Soc B-Biol Sci 360, 1935-1943. [4] Bokulich, N.A., Subramanian, S., Faith, J.J., Gevers, D., Gordon, J.I., Knight, R., Mills, D.A., and Caporaso, J.G. (2013). Quality-filtering vastly improves diversity estimates from Illumina amplicon sequencing. Nat Methods 10, 57-U11. [5] Breiman, L. (2001). Random forests. Mach Learn 45, 5-32. [6] Caporaso, J.G., Kuczynski, J., Stombaugh, J., Bittinger, K., Bushman, F.D., Costello, E.K., Fierer, N., Pena, A.G., Goodrich, J.K., Gordon, J.I., et al. (2010). QIIME allows analysis of high-throughput community sequencing data. Nat Methods 7, 335-336. [7] Chao, A. (1984). Nonparametric Estimation of the Number of Classes in a Population. Scandinavian Journal of Statistics 11, 265-270. [8] Chao, A., and Shen, T.J. (2004). Nonparametric prediction in species sampling. J Agric Biol Environ Stat 9, 253-269. [9] Chao, A., and Yang, M.C.K. (1993). Stopping rules and estimation for recapture debugging with unequal failure rates. Biometrika 80, 193-201. [10] Clarke, K.R. (1993). Non-parametric multivariate analyses of changes in community structure. Australian Journal of Ecology 18, 117-143. [11] Cole, J.R., Wang, Q., Cardenas, E., Fish, J., Chai, B., Farris, R.J., Kulam-Syed-Mohideen, A.S., McGarrell, D.M., Marsh, T., Garrity, G.M., et al. (2009). The Ribosomal Database Project: improved alignments and new tools for rRNA analysis. Nucleic Acids Res 37, D141-D145. [12] DeSantis, T.Z., Hugenholtz, P., Larsen, N., Rojas, M., Brodie, E.L., Keller, K., Huber, T., Dalevi, D., Hu, P., and Andersen, G.L. (2006). Greengenes, a chimera-checked 16S rRNA gene database and workbench compatible with ARB. Appl Environ Microbiol 72, 5069-5072. [13] Edgar, R.C. (2010). Search and clustering orders of magnitude faster than BLAST. Bioinformatics 26, 2460-2461. [14] Edgar, R.C., Haas, B.J., Clemente, J.C., Quince, C., and Knight, R. (2011). UCHIME improves sensitivity and speed of chimera detection. Bioinformatics 27, 2194-2200. [15] Faust, K., and Raes, J. (2012). Microbial interactions: from networks to models. Nat Rev Microbiol 10, 538-550. [16] Hamilton, N. (2016). ggtern: An Extension to 'ggplot2', for the Creation of Ternary Diagrams. [17] Heck, K.L., van Belle, G., and Simberloff, D. (1975). Explicit Calculation of the Rarefaction Diversity Measurement and the Determination of Sufficient Sample Size. Ecology 56, 1459-1461. [18] Huson, D.H., Mitra, S., Ruscheweyh, H.-J., Weber, N., and Schuster, S.C. (2011). Integrative analysis of environmental sequences using MEGAN4. Genome Res 21, 1552-1560. [19] Kemp, P.F., and Aller, J.Y. (2004). Bacterial diversity in aquatic and other environments: what 16S rDNA libraries can tell us. FEMS Microbiol Ecol 47, 161-177. [20] Koljalg, U., Nilsson, R.H., Abarenkov, K., Tedersoo, L., Taylor, A.F.S., Bahram, M., Bates, S.T., Bruns, T.D., Bengtsson-Palme, J., Callaghan, T.M., et al. (2013). Towards a unified paradigm for sequence-based identification of fungi. Molecular Ecology 22, 5271-5277. [21] Langille, M.G.I., Zaneveld, J., Caporaso, J.G., McDonald, D., Knights, D., Reyes, J.A., Clemente, J.C., Burkepile, D.E., Thurber, R.L.V., Knight, R., et al. (2013). Predictive functional profiling of microbial communities using 16S rRNA marker gene sequences. Nature Biotechnology 31, 814-+. [22] Ley, R.E., Hamady, M., Lozupone, C., Turnbaugh, P.J., Ramey, R.R., Bircher, J.S., Schlegel, M.L., Tucker, T.A., Schrenzel, M.D., Knight, R., et al. (2008a). Evolution of mammals and their gut microbes. Science 320, 1647-1651. [23] Ley, R.E., Lozupone, C.A., Hamady, M., Knight, R., and Gordon, J.I. (2008b). Worlds within worlds: evolution of the vertebrate gut microbiota. Nat Rev Microbiol 6, 776-788. [24] Liaw, A., and Wiener, M. (2002). Classification and regression by randomForest. R News 2, 18-22. [25] C., and Knight, R. (2005). UniFrac: a new phylogenetic method for comparing microbial communities. Appl Environ Microbiol 71, 8228-8235. [26] Lozupone, C.A., Hamady, M., Kelley, S.T., and Knight, R. (2007). Quantitative and qualitative beta diversity measures lead to different insights into factors that structure microbial communities. Appl Environ Microbiol 73, 1576-1585. [27] Magoc, T., and Salzberg, S.L. (2011). FLASH: fast length adjustment of short reads to improve genome assemblies. Bioinformatics 27, 2957-2963. [28] McArdle, B.H., and Anderson, M.J. (2001). Fitting multivariate models to community data: a comment on distance-based redundancy analysis. Ecology 82, 290-297. [29] Muegge, B.D., Kuczynski, J., Knights, D., Clemente, J.C., Gonzalez, A., Fontana, L., Henrissat, B., Knight, R., and Gordon, J.I. (2011). Diet drives convergence in gut microbiome functions across mammalian phylogeny and within humans. Science 332, 970-974. [30] Ondov, B.D., Bergman, N.H., and Phillippy, A.M. (2011). Interactive metagenomic visualization in a Web browser. Bmc Bioinformatics 12. [31] Price, M.N., Dehal, P.S., and Arkin, A.P. (2009). FastTree: computing large minimum evolution trees with profiles instead of a distance matrix. Mol Biol Evol 26, 1641-1650. [32] Quast, C., Pruesse, E., Yilmaz, P., Gerken, J., Schweer, T., Yarza, P., Peplies, J., and Gloeckner, F.O. (2013). The SILVA ribosomal RNA gene database project: improved data processing and web-based tools. Nucleic Acids Res 41, D590-D596. [33] Ramette, A. (2007). Multivariate analyses in microbial ecology. FEMS Microbiol Ecol 62, 142-160 [34] Schloss, P.D., Westcott, S.L., Ryabin, T., Hall, J.R., Hartmann, M., Hollister, E.B., Lesniewski, R.A., Oakley, B.B., Parks, D.H., Robinson, C.J., et al. (2009). Introducing mothur: Open-Source, Platform-Independent, Community-Supported Software for Describing and Comparing Microbial Communities. Appl Environ Microbiol 75, 7537-7541. [35] Segata, N., Izard, J., Waldron, L., Gevers, D., Miropolsky, L., Garrett, W.S., and Huttenhower, C. (2011). Metagenomic biomarker discovery and explanation. Genome Biol 12. [36] Shannon, C.E. (1948a). A mathematical theory of communication. The Bell System Technical Journal 27, 379-423. [37] Shannon, C.E. (1948b). A mathematical theory of communication. The Bell System Technical Journal 27, 623-656. [38] Shannon, P., Markiel, A., Ozier, O., Baliga, N.S., Wang, J.T., Ramage, D., Amin, N., Schwikowski, B., and Ideker, T. (2003). Cytoscape: A Software Environment for Integrated Models of Biomolecular Interaction Networks. Genome Res 13, 2498-2504. [39] Simpson, E.H. (1949). Measurement of Diversity. Nature 163, 688. [40] Warton, D.I., Wright, S.T., and Wang, Y. (2012). Distance-based multivariate analyses confound location and dispersion effects. Methods in Ecology and Evolution 3, 89-101. [41] White, J.R., Nagarajan, N., and Pop, M. (2009). Statistical Methods for Detecting Differentially Abundant Features in Clinical Metagenomic Samples. PLoS Comput Biol 5. [42] Yatsunenko, T., Rey, F.E., Manary, M.J., Trehan, I., Dominguez-Bello, M.G., Contreras, M., Magris, M., Hidalgo, G., Baldassano, R.N., Anokhin, A.P., et al. (2012). Human gut microbiome viewed across age and geography. Nature 486, 222-227. --- 附录：文档结构 注意：原始数据文件虽然是文本格式，但是解压缩后一般体积较大，不适合在Windows 下直接打开。 | | | | --- | --- | | A01\_rawdata- \*.fq.gz | - 每样本各自包含的原始数据 | | A02\_sequences- seqs.fna - length.xls - Length\_\*.pdf | - FASTA格式的优质序列文件 - 优质序列的长度分布列表 - 优质序列的长度分布图 | | B01\_OTU- rep.fasta - seqs\_otus.txt - otu.xls - otu\_modified.xls - rep.tre - venn | - FASTA格式的OTU代表序列 - OTU与所包含序列的对应关系 - OTU丰度矩阵 - 精简后的OTU丰度矩阵 - OTU 代表序列的系统发育树 - 共有OTU的Venn图 | | B02\_arare | 稀释曲线分析结果 | | B03\_specaccum- specaccum.pdf | - 物种累积曲线分析结果图 | | B04\_rabund- rabund.xls - rabund.pdf | - 丰度等级曲线分析结果 - 丰度等级曲线图 | | B05\_index- index.txt | - 各样本的Alpha多样性指数表 | | B06\_taxa\*- otu\_table\_L\*.txt | - 各分类水平的物种序列量统计表 | | B07\_taxa\_summary\*- otu\_table\_L\*.txt - bar\_\*.pdf | - 各分类水平的物种相对丰度统计表 - 各分类水平的物种组成和丰度分布图 | | C01\_diff/metastats- summary.txt - diff\_group.png | - Metatstats差异分析的统计结果 - 差异最显著的分类单元的丰度分布图 | | C01\_diff/lefse- lefse\_results.xls - lefse\_effect\_size\_rank.png - lefse\_cladogram.png - lefse\_diff\_taxa/ | - LEfSe统计检验结果列表 - 组间具有显著差异的分类单元 - 基于分类等级树的组间差异分类单元展示图 - 具有显著差异的每个分类单元在不同分组中的相对丰度分布图 | | B08\_megan- \*.pdf | - 物种进化及丰度信息图 | | B09\_graphlan- \*.pdf | - 基于GraPhlAn的样本总体分类等级树图 | | B10\_krona- \*.html | - 基于Krona的分类学组成信息交互式网页 | | B11\_heatmap- otu\_table.html - \*.pdf | - OTU热图的交互式网页 - 结合聚类分析的菌群组成热图 | | B12\_pca- \*.pdf - 3D-PCA | - PCA二维排序图 - PCA三维排序图 | | B13\_bdiv- \*unifrac\_dm.txt - \*unifrac\_pc.txt - \*unifrac\_2d\* - \*unifrac\_3d\* - \*NMDS\*.pdf - \*NMDS\*.txt - hcluster.png - boxplot | - 样本间Unifrac距离矩阵 - Unifrac PCoA分析结果 - PCoA二维排序图 - PCoA三维排序图 - NMDS分析图 - NMDS分析结果 - UPGMA聚类分析图 - 基于UniFrac距离的多组比较箱线图 | | C02\_plsda- plsda.pdf - plsda.vip.xls | - PLS-DA判别分许图 - 各物种的VIP系数值 | | C03\_adonis- adonis\_results.txt | - Adonis/PERMANOVA分析结果 | | C04\_anosim- anosim\_results.txt | - ANOSIM分析结果 | | C05\_random\_forests- confusion\_matrix.txt - feature\_importance\_scores.xls - summary.txt | - 各分组中被错误分类的样本数列表 - 每个OTU的重要度贡献值列表 - 随机森林建模结果 | | D01\_rda- rda.png | - RDA排序图 | | C06\_network- relation\_network\_graph/\*.html - relation\_network\_graph/\*.sif - bipartite\_graph/\*.pdf | - Spearman关联网络的交互式网页 - Cytoscape网络分析的导入数据文件 - OTU—样本二分网络 | | C07\_picrust- predicted\_metagenomes\*.txt - KEGG\_L2\*.pdf - heatmap.pdf - venn/ | - PICRUSt预测的菌群功能谱丰度矩阵 - KEGG第二等级分布图 - 结合聚类分析的KO丰度热图 - 共有功能类群的Venn图 |
